# Comparative analyses and structural insights of the novel cytochrome P450 fusion protein family CYP5619 in Oomycetes

**DOI:** 10.1038/s41598-018-25044-0

**Published:** 2018-04-26

**Authors:** Hans Denis Bamal, Wanping Chen, Samson Sitheni Mashele, David R. Nelson, Abidemi Paul Kappo, Rebamang Anthony Mosa, Jae-Hyuk Yu, Jack A. Tuszynski, Khajamohiddin Syed

**Affiliations:** 1 Unit for Drug Discovery Research, Department of Health Sciences, Central University of Technology, Bloemfontein, 9300 Free State South Africa; 2 College of Food Science and Technology, Huazhong Agricultural University, Wuhan, Hubei Province China; 3 Department of Microbiology, Immunology and Biochemistry, University of Tennessee Health Science Center, Memphis, TN 38163 USA; 4 Department of Biochemistry and Microbiology, Faculty of Science and Agriculture, University of Zululand, KwaDlangezwa, 3886 South Africa; 5 Department of Bacteriology, University of Wisconsin-Madison, 3155 MSB, 1550 Linden Drive, Madison, WI 53706 USA; 6 Department of Physics, University of Alberta, Edmonton, AB T6G 2E1 Canada; 7 Cross Cancer Institute, Department of Oncology, University of Alberta, Edmonton, AB T6G 1Z2 Canada

## Abstract

Phylogenetic and structural analysis of P450 proteins fused to peroxidase/dioxygenase has not been reported yet. We present phylogenetic and *in silico* structural analysis of the novel P450 fusion family CYP5619 from the deadliest fish pathogenic oomycete, *Saprolegnia diclina*. Data-mining and annotation of CYP5619 members revealed their unique presence in oomycetes. CYP5619 members have the highest number of conserved amino acids among eukaryotic P450s. The highest number of conserved amino acids (78%) occurred in the peroxidase/dioxygenase domain compared to the P450 domain (22%). *In silico* structural analysis using a high-quality CYP5619A1 model revealed that CYP5619A1 has characteristic P450 structural motifs including EXXR and CXG. However, the heme-binding domain (CXG) in CYP5619 members was found to be highly degenerated. The *in silico* substrate binding pattern revealed that CYP5619A1 have a high affinity to medium chain fatty acids. Interestingly, the controlling agent of *S. diclina* malachite green was predicted to have the highest binding affinity, along with linoleic acid. However, unlike fatty acids, none of the active site amino acids formed hydrogen bonds with malachite green. The study’s results will pave the way for assessing CYP5619A1’s role in *S. diclina* physiology, including the nature of malachite green binding.

## Introduction

Cytochrome P450 monooxygenases (CYPs/P450s), heme-thiolate proteins, have been in the spotlight for the last five decades because of their critical role in organisms’ primary and secondary metabolism and their biotechnological applications^1^, including their role as drug targets against pathogens^2,3^. P450s are found in species belonging to different biological domains^4^, as well as in non-living entities such as viruses^5^. P450s are known to perform diverse catalytic reactions in a stereo- and regio-specific manner, apart from their primary mono-oxygenation reaction^6,7^.

P450s require two electrons to perform their enzymatic reactions: binding (first electron) and then reductive activation (second electron) of dioxygen^8^. These electrons are supplied by P450 redox proteins, which obtain electrons from co-factors such as NADPH or NADH^9^. Some P450s are found to be fused to their redox proteins and also to other proteins^10–12^. The first P450 fusion protein was reported from the bacterium *Bacillius megaterium* and named CYP102A1/BM-3^13,14^. Because of its fused nature, this P450 was found to be very efficient in catalytic activity^14–16^. CYP102A1 is one of the most extensively studied P450s for structural and catalytic aspects of P450s and has also been extensively engineered to perform different catalytic reactions^17^. Apart from CYP102A1, studies have revealed the presence of different varieties of P450 fusion proteins in species belonging to different biological kingdoms^10,11^. A detailed analysis of different types of P450 fusion proteins (fused to redox proteins or others) has been documented in the literature^2,10,11^.

A recent study reported the presence of a novel P450 fusion protein in the deadliest fish pathogenic oomycete, *Saprolegnia diclina*^12^. This novel P450 fusion protein has been assigned to the CYP5619 family^12^. Six members of CYP5619 found in *S. diclina* are fused to a heme peroxidase/dioxygenase protein. However, the combination of fusion is different compared to the fungal P450 families CYP6001-CYP6005^12^. In the CYP5619 family, the heme peroxidase/dioxygenase protein is fused at the C-terminal end to the P450, whereas in the CYP6001-6005 families the heme peroxidase/dioxygenase protein is fused at the N-terminal end to the P450^12^. Among the CYP6000 series family members, CYP6001A1 from *Aspergillus nidulans* has been shown to be a fatty acid hydroxylase^18^. CYP6001A1 was found to be a bifunctional P450 fusion protein performing oxidation and isomerization reactions by forming psi factors^18^.

To date, structural analysis of the CYP5619 family members or other similar fusion proteins, in terms of structural motifs, CYP6001-CYP6005 family members, has not been reported. Furthermore, it is not known if the CYP5619 family is present in any other organisms apart from oomycetes. In this study, we present phylogenetic and *in silico* structural analysis of the CYP5619 family, including the *in silico* structural and functional analysis of CYP5619A1 from *S. diclina*. Furthermore, we report on the CYP5619 family’s conserved nature and insights into its P450 motifs, EXXR and CXG. Results from this study will pave the way for functional characterization of this novel P450 family member and thus the role of this family in oomycetes’ physiology.

## Methods

### Data mining for CYP5619 homologs

To identify CYP5619 homologs in other organisms, protein blast (BLASTP) was performed at NCBI using six members of the CYP5619 family, namely CYP5619A1, CYP5619B1, CYP5619B2, CYP5619C1, CYP5619D1 and CYP5619D2^12^. The six CYP5619 family members’ protein sequences were retrieved form published data^12^ and used for BLASTP. For each CYP5619 used for BLASTP, a set of 100 hit proteins was downloaded. The hit proteins were subjected to NCBI Batch Web-search tool^19^ for classification into superfamilies based on conserved domains. The domains were searched against the database (CDD–50369 PSSM)^19^, at a cut-off E-value of 0.01, with a composition-corrected scoring. Hit proteins exhibiting the presence of both P450 and peroxidase/dioxygenase domains were retained for further analysis. Furthermore, the proteins that showed a different arrangement of P450 and peroxidase/dioxygenase motifs compared to CYP5619 family members were removed from the analysis. Detailed information on hit proteins and their screening using the NCBI Batch Web-search tool is presented in Supplementary Dataset 1 where CYP5619 P450s and their homologs are highlighted.

### Annotation of P450s

The above selected hit proteins were then subjected to P450 family and subfamily annotation as described elsewhere^20,21^. For assigning the family and subfamily names, the standard rule set by the International P450 Nomenclature Committee^22^ was followed, i.e. P450s within a family share more than 40% amino acid identity and members of subfamilies share more than 55% amino acid identity. P450s that are less than 40% identical to named P450s are assigned to new P450 families. Considering that the P450s are fused proteins, only the P450 motif was used for assigning P450 family and P450 subfamilies to the hit proteins.

### Phylogenetic analysis

The phylogenetic tree of CYP5619 P450s and their homologs was constructed as follows: first, the protein sequences were aligned by MUSCLE embedded in MEGA 7^23^; then, the best-fit substitution model for alignment was determined by the IQ-TREE web server (http://iqtree.cibiv.univie.ac.at/)^24^. Finally, the tree was constructed in MEGA 7 by the maximum likelihood method, along with the best-fit substitution model and 100 bootstrap replications^25^.

### Analysis of amino acid conservation

Analysis of amino acid conservation in CYP5619 family members was carried out as described elsewhere^26^. Briefly, the annotated CYP5619 family members were subjected to PROfile Multiple Alignment with Local Structures and 3D constraints (PROMALS3D)^27^ to identify the number of invariantly conserved amino acids^28^. The conservation index follows numbers above 4, where 9 is the invariantly conserved amino acid across the input sequences. The total number of conserved residues indicated by the number 9 was recorded. The conserved nature of the CYP5619 family was compared to other P450 families from different biological kingdoms as reported elsewhere^26^.

### Generation of EXXR and CXG sequence logos

P450 motifs EXXR and CXG sequence logos were generated as described elsewhere^12,29^. Briefly, CYP5619 family members were aligned using ClustalW multiple alignment using MEGA7^25^. After sequence alignment the EXXR and CXG region amino acids (4 and 16 amino acids respectively), were selected and entered in the WebLogo program (http://weblogo.berkeley.edu/logo.cgi). As a selection parameter, the image format was selected as PNG (bitmap) at 300 dpi resolution. The generated EXXR and CXG logos were used for analysis and compared to the different P450 family EXXR and CXG logos that have been published and are available to the public^12,29^.

### Homology modeling

The Molecular Operating Environment (MOE, Chemical Computing Group) was used to build a 3D model of the CYP5619A1’s P450 domain. Among all templates, CYP120A1 (PDB ID: 2VE3) showed the lowest E-value of the Hidden Markov Model profile and was therefore selected as the template to build the 3D model of CYP5619A1. Homology modeling of CYP5619A1 was performed using a restrained-based approach implemented in MOE. The amino acid sequence of CYP5619A1 was aligned with that of CYP120A1. A set of 10 models was constructed for the target enzyme. The coordinates of the heme in the model were obtained from the crystal structure of CYP120A1 and the homology model was constructed along with those coordinates. The resulting 3D models were optimized and a final model was obtained.

### Energy minimization and validation

The 3D model of CYP5619A1 was optimized using the *tleap* and *sander* programs of the AMBER suite^30^. Energy minimization was performed to minimize stearic collisions and strains without significantly altering the overall structure. Energy computations and minimization were carried out using the Amber14 force field. After optimization the quality of the 3D model of CYP5619A1 was verified using the Protein Structure Analysis (ProSA-Web)^31,32^, ERRAT^33^ and VERIFY 3D^34,35^ programs available from the Structural Analysis and Verification Server (SAVES) (http://nihserver.mbi.ucla.edu/SAVES).

### Molecular docking

The software MOE was used on the final model, to assess the binding sites. A set of sites was found to be likely to accommodate the substrates. Among the sites, the one with more residues, and which appeared to contain the heme group, was selected for docking studies. Three-dimensional structures of fatty acids of different lengths and saturation states alongside with the organic compound malachite green were obtained from PDBeChem: Ligand Dictionary (www.ebi.ac.uk/pdbe-srv/pdbechem/) and used in the docking of the target model. Ligands used in the study are listed in Table S1. The CYP5619A1 model was prepared for docking in MOE and AutoDockTools 1.5.6^36^. MOE was used to correct the protonation and to remove the solvent. The different ligands were all prepared for docking in AutoDockTools, following the same steps as the target protein: protonation, addition of charges, merging of non-polar H+ and assignment of atom types. Partial charges of ligands and protein were generated using the Gasteiger method with the aid of AutoDockTools. Non-polar hydrogens were merged and a AD4 atom types were assigned. A cubic grid having 60 × 60 × 60 grid points per side and spacing of 0.375 Å was set around the substrate recognition site of the target P450 model. The grid was positioned onto the substrate access channel extending into the binding pocket of the model. Affinity maps of the grid were calculated using the AutoGrid program. The AutoDock 4.0 program was used to dock 12 ligands into the active-site cavity of the target model using the Lamarckian genetic algorithm, consisting of 200 runs and 270 000 generations, with the maximum number of energy evaluations set to 2.5 × 106. The resulting docked conformations within 2.0 Å root mean square deviation (RMSD) tolerance were clustered and analysed using AutoDockTools. The best results were selected according to the outputted clustering histogram. Therefore conformations with the lowest binding energies of the biggest cluster and with the closest interaction to the heme iron were selected for each ligand. The representative conformation for each cluster was chosen as the best pose for each ligand and the receptor-ligand complex’s site view was rendered in MOE.

## Results and Discussion

### CYP5619 family is only present in oomycetes

**Figure 1:**
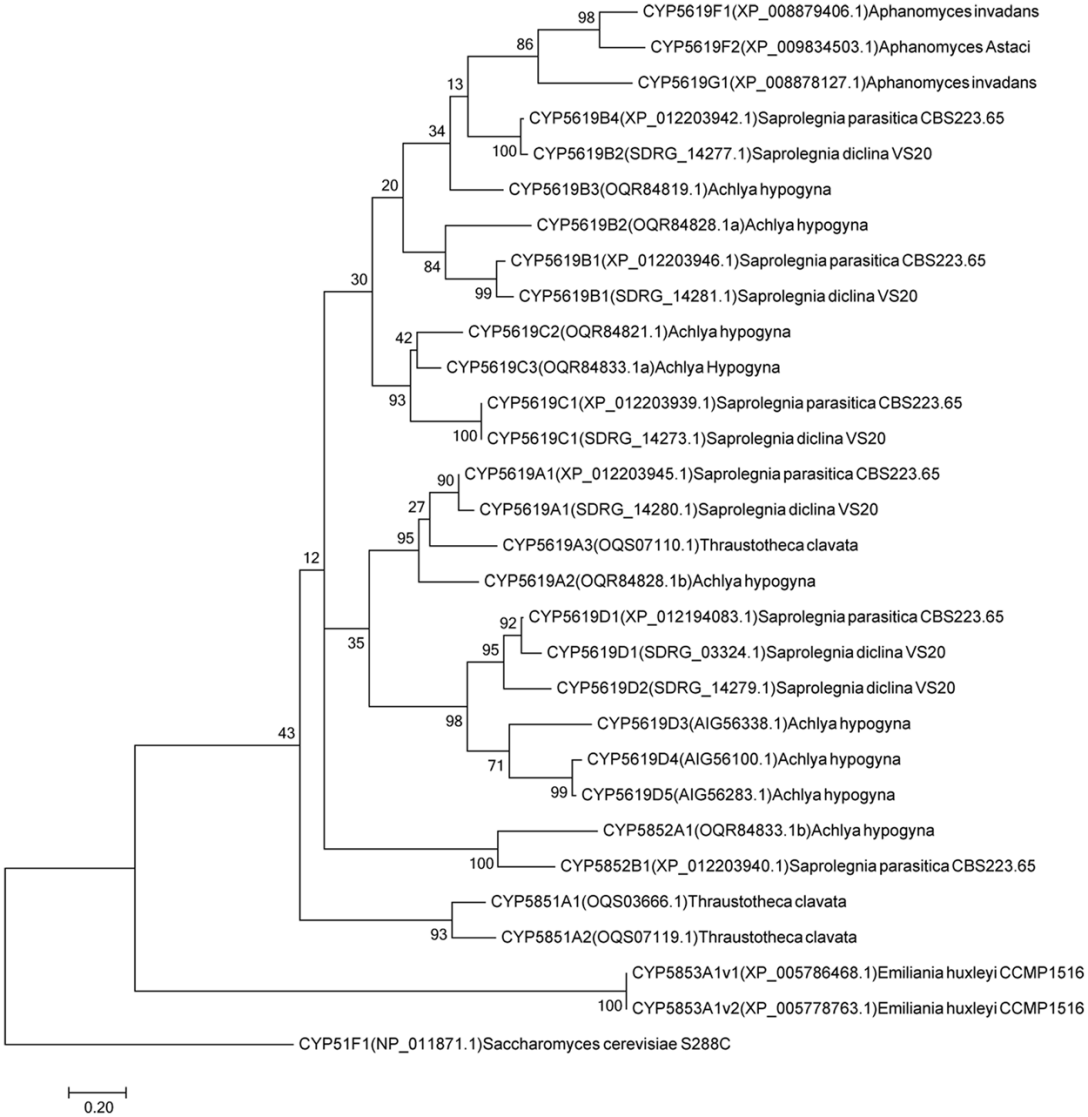
Evolutionary analysis of CYP5619 family members and their homolog P450s. Thirty P450s were used in the analysis. The P450 motif sequences used for phylogenetic analysis are presented in Table S3. The *S. cerevisiae* CYP51 P450 sequence was used as an out-group.

Data mining and annotation of CYP5619 homologs across biological kingdoms revealed the presence of CYP5619 family members only in oomycetes (as of 21^st^ November, 2017) (Fig. 1 and Table 1). The analysis revealed the presence of 17 CYP5619 P450s in five oomycetes, excluding the six CYP5619 family members from *S. diclina* as previously reported^12^ (Table 1). Among oomycetes, the highest number of CYP5619 members was found in *Achlya hypogyna* (8 P450s) followed by *S. diclina* (6 P450s), *S. parasitica* (5 P450s) and *Thraustotheca clavata* (2 P450s). *Aphanomyces invadans* and *Aphanomyces astaci* both have a single CYP5619 member in their genome (Table 1). The CYP5619 family members annotated in this study are listed in Table S2. Our analysis revealed the presence of six CYP5619 family homologs in *Oomycota* and *Prymnesiophyceae* (Fig. 1 and Table 1). Because of a low sequence identity to the CYP5619 family, these homolog P450 fused proteins were assigned to new P450 families, namely CYP5851-CYP5853. The CYP5851 and CYP5852 families were present in oomycetes and the CYP5853 family was found in a phytoplankton. Among oomycetes, *A. hypogyna* and *S. parasitica* have one CYP5619 homolog P450 each, namely CYP5852A1 and CYP5852B1 respectively, and *T. clavata* has two homologs annotated as CYP5851A1 and CYP5851A2. *Emiliania huxleyi* CCMP1516, a phytoplankton, has two CYP5619 homologs, namely CYP5853A1v1 and CYP5853A1v2 (Table 1 and Table S2). Alignment of P450 fused proteins with their counterparts in the phylogenetic tree (Fig. 1) indicates that our annotation of P450 fused proteins is correct. All these homologous P450s belonging to CYP5851-CYP5853 families have the same structural P450 motif and dioxygenase/peroxidase as CYP5619 family members, i.e N-terminal P450 motif and C-terminal dioxygenase/peroxidase.

**Table 1:** Comparative analysis of CYP5619 family members and their homolog P450s.

Species name	Taxonomic group	No. of CYP5619 P450s	CYP5619 subfamilies						Homolog P450 families
			A	B	C	D	F	G	
*Achlya hypogyna*	*Oomycota*	8	1	2	2	3			CYP5852A1
*Thraustotheca clavata*	*Oomycota*	1	1						CYP5851A1 and CYP5851A2
*Aphanomyces invadans*	*Oomycota*	2					1	1	
*Aphanomyces astaci*	*Oomycota*	1					1		
*Saprolegnia parasitica* CBS223.65	*Oomycota*	5	1	2	1	1			CYP5852B1
*Saprolegnia diclina* VS20	*Oomycota*	6	1	2	1	2			
*Emiliania huxleyi CCMP1516*	*Prymnesiophyceae*	0							CYP5853A1v1 and CYP5853A1v2

### CYP5619 subfamily distribution in Oomycetes

P450s subfamily-level comparison revealed the presence of six CYP5619 subfamilies, namely A–D, F and G, in oomycetes (Table 1). Among the CYP5619 subfamilies, subfamilies B and D had the highest number of members (six), followed by subfamilies A and C, which had the same number of members (four), and subfamilies F and G, which had only one member (Fig. 1 and Table 1). The CYP5619 subfamily distribution revealed that *A. hypogyna*, *S. parasitica* and *S. diclina* had four subfamilies, namely A–D, in their genomes and that *T. clavata* had only one CYP5619 belonging to subfamily A. Subfamily F was present only in *A. invadans* and *A. astaci* (Table 1). Subfamily G was only present in *A. invadans*. Future functional analysis may reveal the significance of CYP5619 subfamily distribution patterns, if any, in oomycete physiology.

### CYP5619 family ranked sixth among P450 families

In a recent study, Parvez and coworkers^26^ analyzed P450 families from different biological kingdoms and identified the highly conserved P450 families based on a number of conserved residues in a P450 family. The analysis revealed that the top 10 conserved P450 families belonged to the kingdom Bacteria^26^. As the CYP5619 family is newly discovered and more family members have been identified in different oomycetes, in this study, we also assessed the CYP5619 family placement in terms of amino acid conservation. In order to identify the conservation rank, CYP5619 family members were subjected to PROMALS3D analysis (Fig. S1). PROMALS3D analysis revealed the presence of 200 amino acids invariantly conserved in CYP5619 family members (Table 2). Comparative analysis with other P450 families from different biological kingdoms showed that the CYP5619 family occupies the sixth rank in terms of amino acid conservation among P450 families. This is quite a high number of conserved amino acids for a eukaryotic P450 family and CYP5619 is the first eukaryotic P450 family that forms part of the top 10 conserved families (Table 2). Furthermore, the CYP5619 family also shows the highest number of amino acids at position 7 compared to the top 10 ranked P450 families (Table 2). This suggests that CYP5619 family members have been subjected to fewer mutations during evolution, thus possibly indicating these family members’ key role in oomycetes’ physiology. One interesting observation is that most of the conserved amino acids are present in the C-terminal part, i.e. dioxygenase/peroxidase motif. The analysis of conserved amino acids in different motifs revealed the presence of 44 conserved amino acids in the P450 motif and 155 conserved amino acids in the dioxygenase/peroxidase motif, indicating that the P450 motif is highly prone to amino acid substitutions resulting in the generation of new CYP5619 subfamilies, thus contributing to the lowest P450 family diversity in oomycetes, as described previously^12^.

**Table 2:** Comparative amino acid conservation analysis of CYP5619 family with top 10 ranked P450 families^12^.

P450 family	Number of member P450s	Kingdom	PROMALS3D conservation index	Rank (highest to lowest conservation)				
			5	6	7	8	9	
CYP141	29	Bacteria	0	0	0	0	389	1
CYP51	50	Bacteria	11	102	0	0	264	2
CYP137	38	Bacteria	145	0	0	0	251	3
CYP121	34	Bacteria	0	0	0	0	233	4
CYP132	39	Bacteria	175	0	0	0	217	5
**CYP5619**	**23**	**Stramenopila (oomycetes)**	**118**	**38**	**170**	**0**	**199**	**6**
CYP124	71	Bacteria	52	35	59	0	170	7 (formerly 6)
CYP188	67	Bacteria	62	0	100	0	141	8 (formerly 7)
CYP123	74	Bacteria	62	0	82	0	137	9 (formerly 8)
CYP108	67	Bacteria	52	12	92	0	134	10 (formerly 9)
CYP126	78	Bacteria	65	16	98	0	132	11 (formerly 10)

The conservation index score is obtained as described in the section on methods, following the procedure documented in the literature^28^. The conservation score (5–9) obtained *via* PROMALS3D is shown in the table where the number “9” indicates conserved amino acids in P450 members. P450 families were arranged in order of the highest to the lowest number of amino acids conserved.

### CYP5619 family has a highly degenerated heme-binding motif

**Figure 2:**
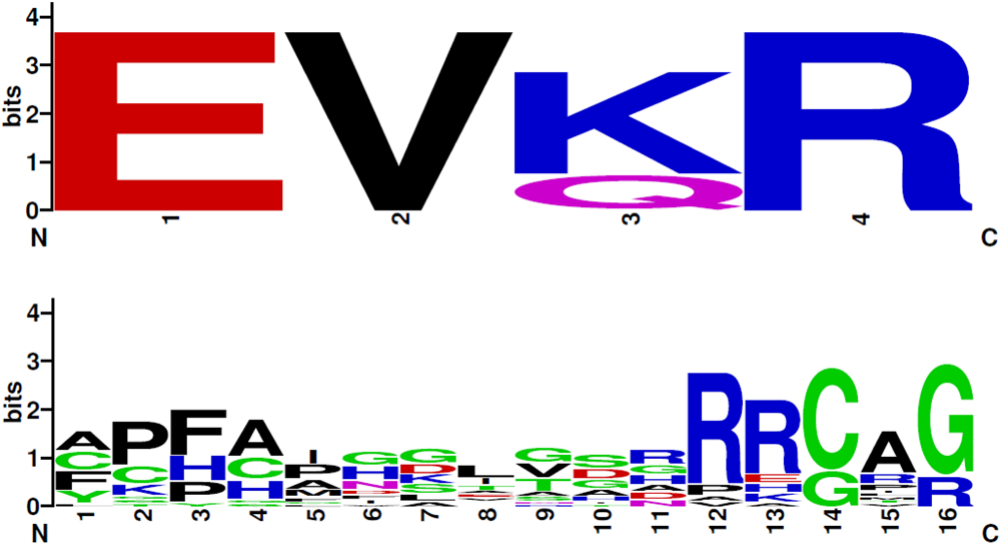
Analysis of amino acid patterns at EXXR and CXG motif in CYP5619 P450 family. Twenty-three CYP5619 P450 sequences were used to generate WebLogos. The EXXR and CXG sequences used to generate WebLogos are presented in Table S4.

Comprehensive comparative study on P450 motifs EXXR and CXG revealed that each P450 family has a characteristic signature of amino acid patterns at these motifs^12,29^. The use of EXXR and CXG amino acid patterns for further verification of P450 family assignment is gaining momentum^37–42^. The fact that the CYP5619 family was recently discovered and that more members have been identified (in this study) gives us an opportunity to assess CYP5619 family EXXR and CXG motifs-amino acid patterns. The analysis of these two P450 signature motifs revealed the presence of an E-V-K/Q-R amino acid pattern at the EXXR motif in the CYP5619 family (Fig. 2). The comparison with other P450 families revealed that the EXXR motif amino acid patterns of CYP5619 family is to some extent matched with the CYP2 P450 family where the CYP2 family has an E-V/I-Q-R combination as described elsewhere^29^. In contrast to amino acid patterns at the EXXR motif, the CYP5619 family has a highly degenerated amino acid pattern at the CXG motif (Fig. 2). It is well established that most P450s have a canonical sequence of FXXGXRXCXG at the heme-binding motif^43,44^, with some exceptions^29,45,46^. As shown in Fig. 2 and Table S4, all CYP5619 family members have a degenerate amino acid pattern at this motif. Among the CYP5619 family members, six members (CYP5619D1 from *S. parasitica* and *S. diclina*; CYP5619D2 from *S. diclina*; CYP5619D3-D5 from *A. hypogyna*) even lack the conserved cysteine in the CXG motif (Fig. 2 and Table S4). The presence of the degenerated amino acid pattern at the CXG motif is not a new phenomenon and P450 families, including the CYP6000 series, have been reported to have degenerated heme-binding motifs^29,45,46^. Considering the structural similarity between CYP5619 and CYP6000-series P450 families based on the presence of the same motifs, it can be assumed that some CYP5619 family members may have the same degenerated amino acid patterns at the CXG motif the as observed in the CYP6000-series P450s^47^. P450s with a degenerated heme-binding motif are possibly involved in performing non-traditional P450 reactions. As CYP2 and CYP5619 families have similar amino acid patterns at the EXXR motif, it would be interesting to see their phylogenetic relation and also assess their phylogenetic grouping with the 113 P450 families from different biological kingdoms that have been subjected to clade analysis, as described elsewhere^26^.

### CYP5619A1 contains characteristic P450 structural elements

Considering the interesting aspects of the CYP5619 family as described above, it seemed interesting to look at CYP5619 members’ structure and function. For this reason CYP5619A1 from fish pathogen oomycete *S. diclina* was selected for further study.

**Figure 3:**
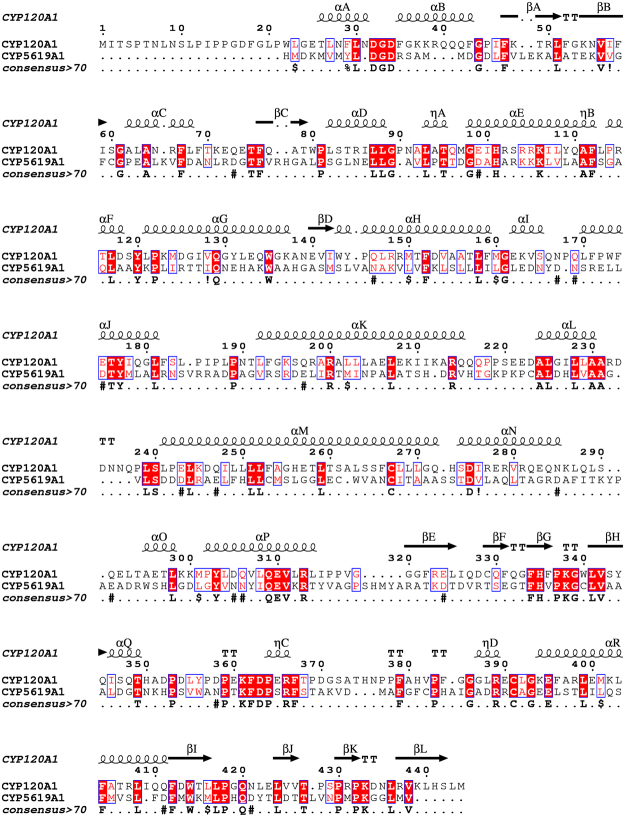
Sequence alignment of CYP5619A1 with template CYP120A1 (PDB ID: 2VE3). Helices are represented by coils and β-sheets are shown as arrows. The P450 consensus motifs EXXR and CXG are highlighted in yellow. Columns with residues that are more than 70% similar according to physico-chemical properties (threshold set to 0.7) are framed in red. The figure was rendered by ESPript 3.0^57^.

**Figure 4:**
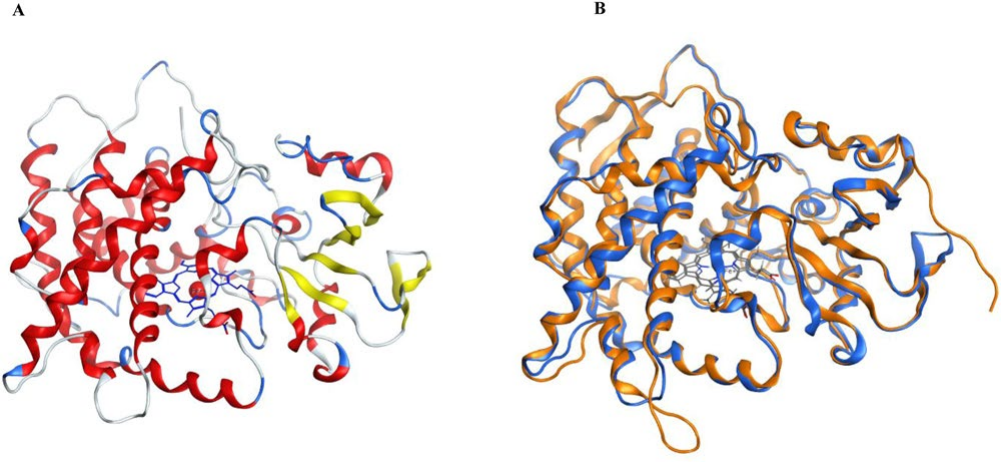
*In silico* structural analysis of CYP5619A1. (**A**) 3D model of CYP5619A1 with heme cofactor. Secondary structures are displayed in red (helices), yellow (sheets) and blue (coils and turns). (**B**) Comparative structural analysis of CYP5619A1 model with CYP120A1. Superimposed image of CYP5619A1 model (blue) with CYP102A1 crystal structure (orange) is shown in the Figure. The CYP5619A1 structure is shown in blue and the template CYP120A1 structure is shown in orange.

CYP5619A1 P450’s 3D model was built using the template CYP120A1 from *Synechocystis* sp. PCC 6803^48^. CYP120A1 was the first cyanobacterial P450 to be crystallized and the structures were solved as substrate free and all-trans-retinoic acid-bound forms, at 2.4 and 2.1 Å resolutions, respectively^48^. CYP120A1 was the best hit; it has 28% sequence identity to CYP5619A1. The low sequence identity is due to the fact that CYP5619A1 belongs to a novel P450 family and P450s belonging to this family or P450s with the same structural motifs do not have available solved crystal structures. Sequence alignment between CYP5619A1 and CYP120A1 showed the presence of characteristic P450 motifs including the highly conserved motifs EXXR and CXG in CYP5619A1 (Fig. 3). Based on the CYP120A1 template, a 3D model of CYP5619A1 was constructed along with its heme cofactor (Fig. 4A). The 3D model of CYP5619A1 is a monomer, folded into α/β domains characteristic of a P450 (Figs. 3 and 4). The β-sheets tend to form the hydrophobic substrate channel. The residues Glu287-Arg290 appeared to form the EXXR motif. This motif is involved in stabilizing the core structure of the protein and is on the proximal side of the heme as described elsewhere^49^. Furthermore, the heme (displayed in sticks in Fig. 4A) is bound to the absolutely conserved cysteine at position 371, which is the fifth ligand of the heme iron and responsible for the typical 450 nm Soret absorbance found in CO-bound P450s^50–52^. Structural comparison showed that CYP5619A1 and CYP120A1 have the same structural organization, except minor differences that were found in the loop and N-terminal regions (Fig. 4B). It is a well-known fact that all P450s differ at N-terminal regions and thus it is not a new phenomenon^44^.

Homology modeling usually results in the production of protein models with quite unfavorable bond lengths, bond angles, torsion angles and contacts. In that case, it is essential to minimize the energy in order to regularize local bond and angle geometry, and to relax close contacts in the geometric chain. Thus, in this study, the 3D model of CYP5619A1 was subjected to optimization and validation as described in the methodology. The CYP5619A1 3D model was optimized using the *tleap* and *sander* programs of the AMBER suite. Energy computations and minimization were carried out using the Amber14 force field. The optimized 3D model was subjected to different validation programs.

The optimized 3D model of CYP5619A1 from *S. diclina* has a z-score of −7.61, indicating good overall model quality (Fig. S2). ERRAT has been termed an “overall quality factor” for non-bonded atomic interactions, with higher scores indicating higher quality. The generally accepted range is >95 for a high-quality model. For the optimized 3D model of CYP5619A1, the overall quality factor predicted by the ERRAT server was 96.226 (Fig. S3). The Verify 3D server predicted that 86.36% of the residues in the CYP5619A1 model would have an average 3D-1D score >0.2 (Fig. S4), thereby confirming the good quality of the model, since the minimum percentage for good quality is 80.

For more assurance on the quality of the model, the CYP5619A1 3D model and CYP120A1 structure were superimposed and compared based on the distance between their Cα backbones (Fig. S5). The superimposition showed a high match between CYP5619A1 and CYP120A1, with some minor mismatches around loops and also in the N- and C-terminal regions (Fig. S5). It is a well-known fact that P450s differ in the N- and C-terminal regions and also in the loop regions, as these regions are highly variable in the primary sequence^44^. The overall RMSD value between the CYP5619A1 model and its template CYP120A1 was calculated to be 0.951 Å, which is a highly acceptable range and thus indicates the good quality of the generated model.

### CYP5619 active site is highly hydrophobic

**Figure 5:**
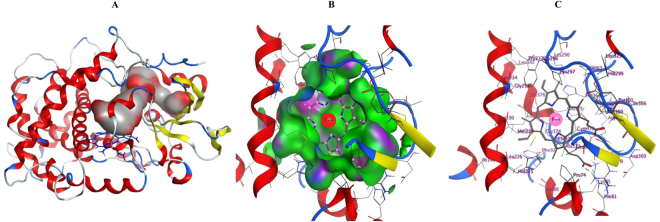
Active site analysis of CYP5619A1. (**A**) Active site cavity of CYP5619A1. The active site cavity is shown with the substrate access channel in grey (hydrophobic site) and red (hydrophilic site) surface. (**B** & **C**) Active site view of the binding pocket of CYP5619A1. (**B**) The pocket is displayed with MOE ActiveLP color coding (Blue: Mild polar; Green: Hydrophobic; Pink: H-Bonding) and shows a pattern of high hydrophobicity. (**C**) Residues forming the pocket are labelled. The amino acids lining the active site cavity are shown in Table S5. Secondary structures are displayed in red (helices), yellow (sheets) and blue (coils and turns). The heme prosthetic group appearing at the center of the active site is shown along with iron atom in a ball shape.

After constructing the high quality CYP5619A1 3D model, different potential binding sites of CYP5619A1 were searched using MOE to find the active site. When the search was complete, the largest site was automatically displayed on the structure, as shown in Fig. 5A. Furthermore, the binding pocket was viewed and displayed (Fig. 5B,C). As shown in Fig. 5, the heme is in the core of the pocket, which appears to be highly hydrophobic, suggesting a very high affinity with the docked fatty acids, as shown in the docking results in the following section. The amino acids that are part of the CYP5619A1 active site are listed in Table S5. Analysis of active site cavity amino acids revealed that the CYP5619 active site contains 40% of hydrophobic amino acids, 34% neutral, 10% basic and 10% acidic. This clearly suggests that the CYP5619 active site is indeed hydrophobic in nature.

### CYP5619A1 showed highest binding affinity to medium chain length fatty acids

As the CYP5619 family was recently discovered in oomycetes^12^ and no functional data is available, in this study, *in silico* functional analysis was carried out using different fatty acids and malachite green as possible substrates. The rationale for using fatty acids as possible substrates is that the CYP5619A1 motifs (P450 and dioxygenase/peroxidase) match CYP6001A1 P450^18^, except for a difference in the motifs’ arrangement^12^. CYP6001A1 from *A. nidulans* was shown to be a fatty acid hydroxylase^18^. Furthermore, *S. diclina* is a well-known fish killer and possibly uses the host’s fatty acids, as fish contain abundant fatty acids in their bodies^53^. In addition to this, based on the template CYP120A1 substrate, i.e. retinoic acid, fatty acids were selected for binding analysis. Malachite green has been widely used to treat oomycete infections^54^ and studies have shown that P450 enzymes perform reduction and demethylation of this dye^55,56^. It would be interesting to assess malachite green binding affinity to CYP5619A1, as quite a number of CYP5619 family members are present in this fish killer^12^.

**Figure 6:**
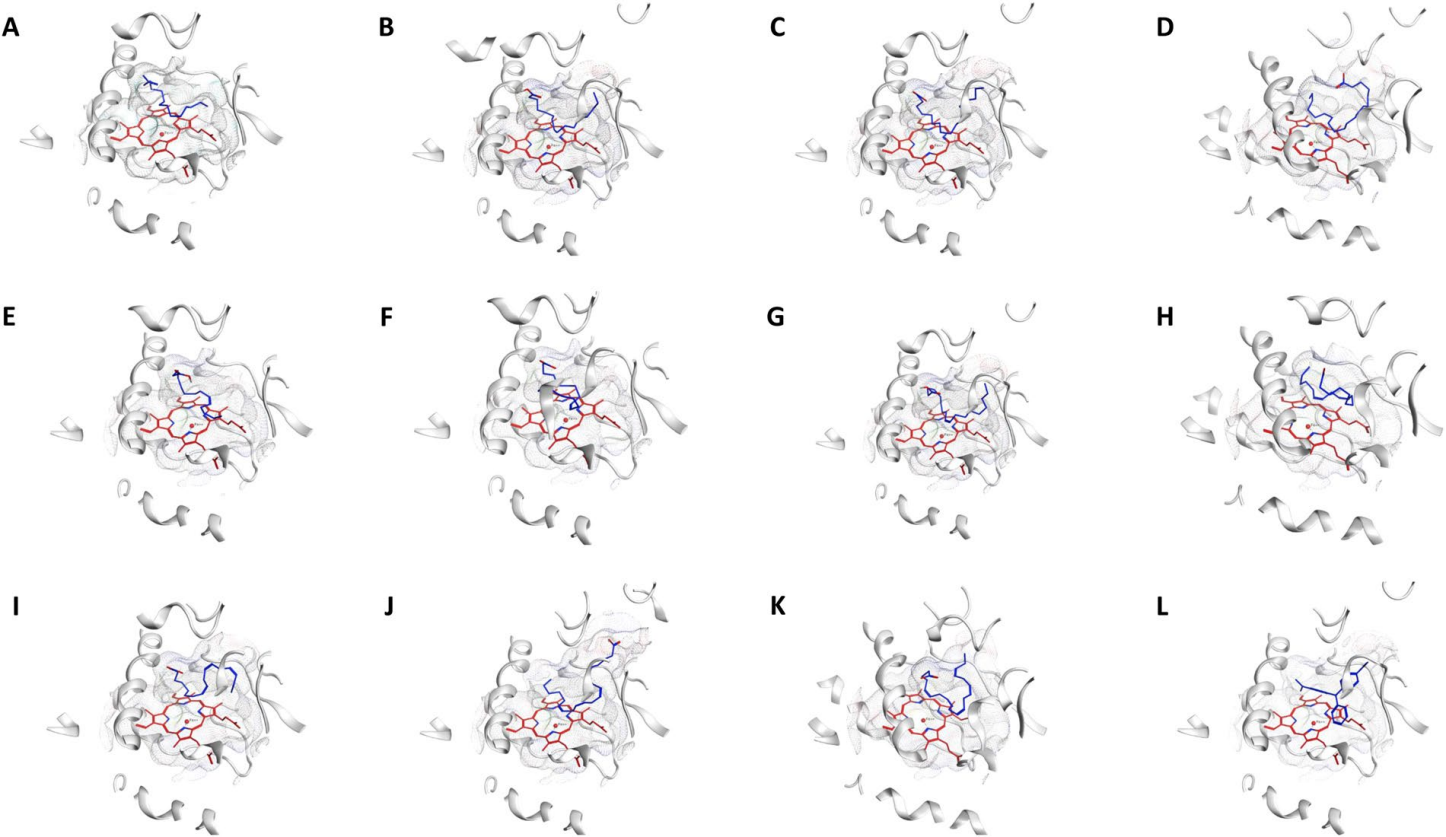
Analysis of fatty acids (**A**–**K**) and malachite green (**L**) binding with CYP5619A1 from *S. diclina*. Fatty acids used in this study are (**A**) myristic acid, (**B**) palmitic acid, (**C**) stearic acid, (**D**) icosanoic acid, (**E**) myristoleic acid, (**F**) palmitoleic acid (**G**) oleic acid, (**H**) linoleic acid (**I**) alpha-linolenic acid, (**J**) arachidonic acid and (**K**) eicosapentaenoic acid. The heme prosthetic group is displayed in red at the center of the active site. The ligands are displayed in blue sticks. Secondary structures surrounding the active site are shown in white and the receptor’s surface is displayed as a white mesh.

**Figure 7:**
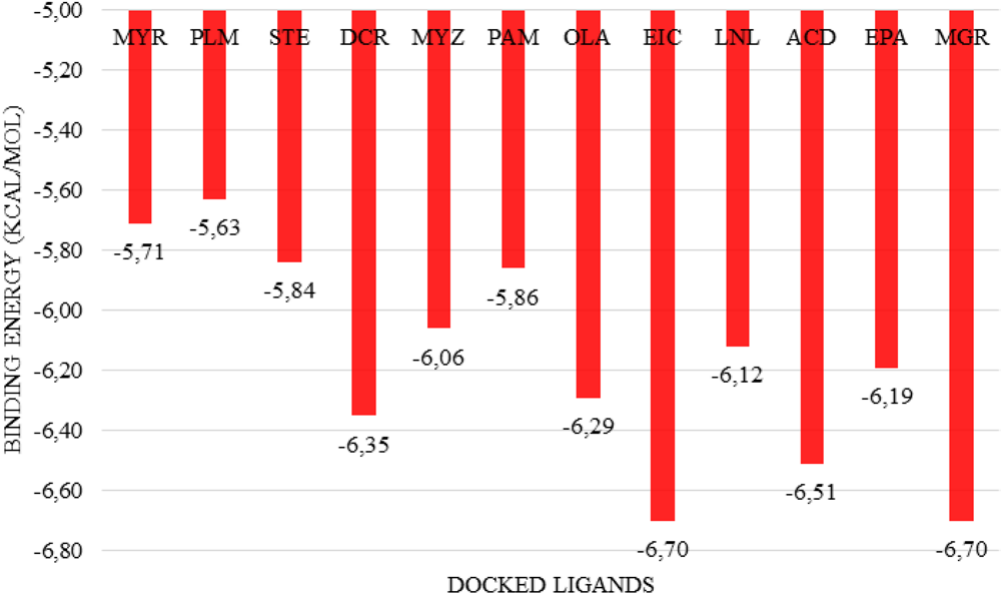
Graphic representation of the free binding energies of the docked possible substrates and malachite green. Abbreviations: MYR: myristic acid; PLM: palmitic acid; STE: stearic acid; DCR: icosanoic acid; MYZ: myristoleic acid; PAM: palmitoleic acid; OLA: oleic acid; EIC: linoleic acid; LNL: alpha-linolenic acid; ACD: arachidonic acid; EPA: eicosapentaenoic acid; MGR: malachite green.

The possible fatty acid substrates, myristic acid, palmitic acid, stearic acid, icosanoic acid, myristoleic acid, palmitoleic acid, oleic acid, linoleic acid, alpha-linolenic acid, arachidonic acid and eicosapentaenoic acid, were selected for *in silico* structure-based interaction analysis with CYP5619A1 (Figs 6 and 7). The molecular docking studies showed that linoleic acid is more tightly bound, compared to all other fatty acids (Fig. 7). The order of binding is as follows: linoleic acid >arachidonic acid >icosanoic acid >oleic acid >eicosapentaenoic acid >alpha-linolenic acid >myristoleic acid >palmitoleic acid >stearic acid >myristic acid >palmitic acid (Fig. 7). The binding pattern revealed that CYP5619A1 prefers medium chain fatty acids compared to short chain and bulky chain fatty acids. Furthermore, CYP5619A1 showed a higher affinity to short chain unsaturated fatty acids compared to their saturated counterparts.

An interesting result was that malachite green showed the highest binding affinity, together with linoleic acid (Fig. 7). In order to understand the binding affinity preference of CYP5619A1 with malachite green and linoleic acid better, the binding energies of 10 conformations for both ligands were analyzed (Fig. S6). As shown in Fig. S6, the remaining conformation of malachite green bound to the protein with a lower free binding energy compared to that of linoleic acid. However, the free binding energies of the best conformation for both ligands were the same (Fig. 7). This suggests that either malachite green can be a substrate for CYP5619A1 or it can be a good inhibitor. Experimental analysis with pure CYP5619A1 is needed to confirm the nature of binding of malachite green and other fatty acids to this P450.

### Arg14 and Arg162 forming hydrogen bonds with fatty acids

After successful completion of ligand binding affinity analysis, further work was carried out to assess the amino acids binding to these ligands (Fig. 6 and Table 3). Comprehensive comparative analysis of amino acids binding to different ligands was carried out (Fig. 6 and Table 3). Among the amino acids, Arg162 was found to form hydrogen bonds with 10 fatty acids and Arg14 was found to form a hydrogen bond with the remaining fatty acid: arachidonic acid (Table 3). Interestingly, none of the interacting amino acids formed a hydrogen bond with malachite green, suggesting that the compound may inhibit CYP5619A1 (Table 3). The analysis of conservation among the interacting amino acids revealed that a total of 21 amino acids were found to interact with 12 ligands (11 fatty acids and malachite green) (Table S6). Of the amino acids, Met229 and Pro297 were both interacting with all 12 ligands, followed by Arg162, Gly232 and Gly233, which showed interaction with 10 of the ligands (Table S6). A detailed analysis of each of the amino acids interacting with different ligands is presented in Table S6. Furthermore, all 21 amino acids interacting with the ligands were found to be part of the active site cavity identified above (Table 3 and S5). The high conservation of amino acids interacting with ligands and the presence of these amino acids in the active site cavity suggest that our binding analysis is correct and all ligands were properly docked in the active site cavity.

**Table 3:** Amino acid residues interacting with the different ligands.

Ligand code	Interacting residues
MYR	Arg162 (2HB), Cys228, Met229, Gly232, Pro297, His299, Met300, HEM413
PLM	Leu61, Leu65, Met158, Arg162 (2HB), Cys228, His225, Met229, Pro297, His299, Tyr301, HEM413
STE	Leu61, Leu65, Arg162 (2HB), Cys228, Met229, Gly232, Gly233,Trp237, Pro297, Tyr301, HEM413
DCR	Leu61, Arg162 (1HB), Met229, Gly232, Gly233, Trp237, Pro297, His299, Thr407, HEM413
MYZ	Pro74, Arg162 (2HB), His225, Cys228, Met229, Gly232, Gly233, Pro297, HEM413
PAM	Leu69, Pro74, Arg162 (2HB), His225, Cys228, Met229, Gly232, Gly233, Pro297, HEM413
OLA	Leu61, Leu65, Arg162 (2HB), His225, Cys228, Met229, Gly232, Gly233, Pro297, His299, Met300, Tyr301, HEM413
EIC	Leu69, Leu161, Arg162 (2HB), His225, Cys228, Met229, Gly232, Gly233, Trp237, Pro297, Leu408, HEM413
LNL	Leu61, Arg162 (2HB), Cys228, Met229, Gly232, Gly233, Trp237, Pro297, His299, Met300, Tyr301, HEM413
ACD	Tyr9, Arg14 (1HB), Leu61, Met229, Gly233, Trp237, Pro297, His299, HEM413
EPA	Leu61, Leu65, Pro74, Arg162 (1HB), His225, Cys228, Met229, Gly232, Gly233, Pro297, Thr407, HEM413
MGR	Leu61, Leu65, Pro74, Met229, Gly232, Gly233, Trp237, Pro297, His299, Tyr301, HEM413

Abbreviations: MYR: myristic acid; PLM: palmitic acid; STE: stearic acid; DCR: icosanoic acid; MYZ: myristoleic acid; PAM: palmitoleic acid; OLE: oleic acid; EIC: linoleic acid; LNL: alpha-linolenic acid; ACD: arachidonic acid; EPA: eicosapentaenoic acid; MGR: malachite green; 1HB, 1-hydrogen bond; 2HB, 2-hydrogen bonds. The number of hydrogen bonds an amino acid forms with a particular ligand is shown in parenthesis.

To our knowledge this study is the first report on *in silico* structural and phylogenetic analysis of the CYP5619 family. This study shed light on the novel CYP5619 P450 family distribution and its conservation in terms of primary structure. *In silico* structural and binding studies showed that CYP5619A1 binds tightly to medium chain fatty acids. However, unravelling the nature of malachite green, the controlling agent of *S. diclina*, binding to CYP5619A1 will be very interesting, considering that no active site amino acid formed hydrogen bonds with malachite green, suggesting that it is an inhibitor or substrate for CYP5619A1.

## Electronic supplementary material

Supplementary Information

Dataset 1
