# Supplementary material for: Comparative analyses and structural insights of the novel cytochrome P450 fusion protein family CYP5619 in Oomycetes
Source: Sci Rep. 2018 Apr 26;8:6597. doi: 10.1038/s41598-018-25044-0 (PMC5919972; doi:10.1038/s41598-018-25044-0)
Supplement: Supplementary file 1 — Supplementary Information [file 41598_2018_25044_MOESM1_ESM.docx]

**Comparative analyses and structural insights of the novel cytochrome P450 fusion protein family CYP5619 in Oomycetes**

Hans Denis Bamal^1^, Wanping Chen^2^, Samson Sitheni Mashele^1^, David R Nelson^3^, Abidemi Paul Kappo^4^, Rebamang Anthony Mosa^4^, Jae-Hyuk Yu^5^, Jack A Tuszynski^6,7*^, Khajamohiddin Syed^4*^

^1^Unit for Drug Discovery Research, Department of Health Sciences, Central University of Technology, Bloemfontein 9300, Free State, South Africa

^2^College of Food Science and Technology, Huazhong Agricultural University, Wuhan, Hubei Province, China

^3^Department of Microbiology, Immunology and Biochemistry, University of Tennessee Health Science Center, Memphis, TN, 38163, USA.

^4^Department of Biochemistry and Microbiology, Faculty of Science and Agriculture, University of Zululand, KwaDlangezwa 3886, South Africa

^5^Department of Bacteriology, University of Wisconsin-Madison, 3155 MSB, 1550 Linden Drive, Madison WI 53706, USA

^6^Department of Physics, University of Alberta, Edmonton, AB T6G 2E1, Canada

^7^Cross Cancer Institute, Department of Oncology, University of Alberta, Edmonton, AB T6G 1Z2, Canada

* Corresponding authors’ email: jackt@ualberta.ca & [khajamohiddinsyed@gmail.com](mailto:khajamohiddinsyed@gmail.com)

**Table S1. Ligands (fatty acids and malachite green) used in the study**.

| **Saturated fatty acids** | **Unsaturated fatty acids** |
| --- | --- |
| **C14:0 Myristic acid**  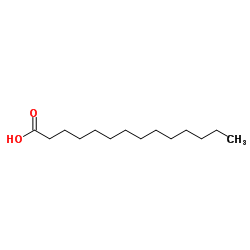 | **C14:1 Δ9Z Myristoleic acid**  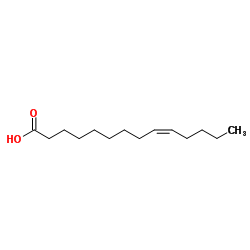 |
| **C16:0 Palmitic acid**  **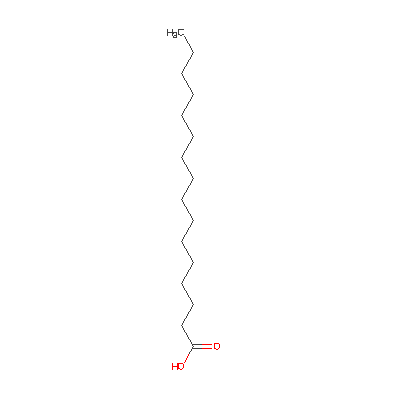** | **C16:1 Δ9Z Palmitoleic acid**  **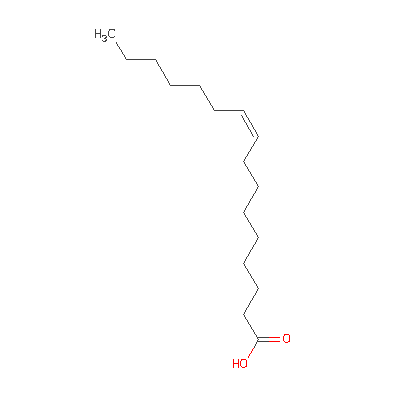** |
| **C18:0 Stearic acid**  **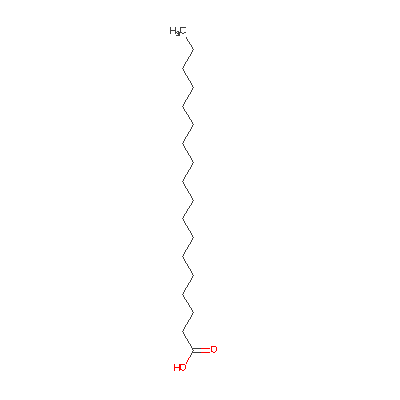** | **C18:1 Δ9Z Oleic acid**  **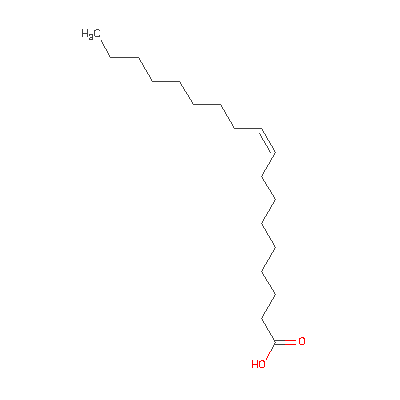** |
| **C20:0 Icosanoic acid**  **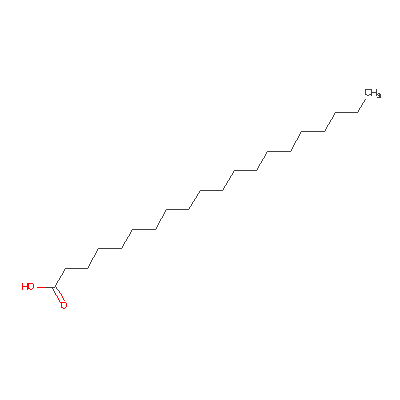** | **C18:2 Δ9Z,12Z Linoleic acid**  **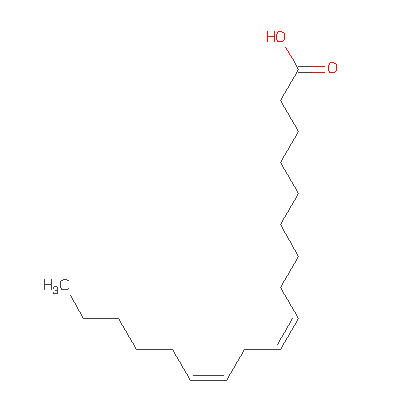** |
| **ORGANIC COMPOUND** | **C18:3 Δ9Z,12Z,15Z alpha-linolenic acid**  **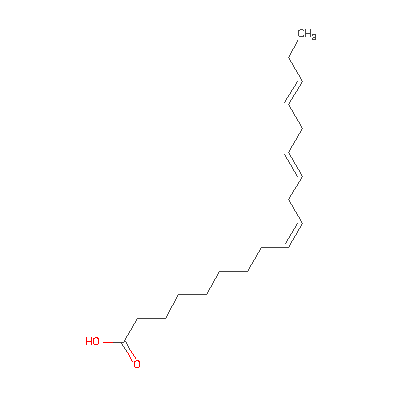** |
| **Malachite green**  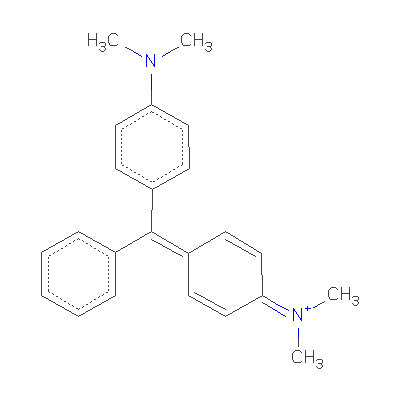 |  |
|  | **C20:4 Δ5Z,8Z,11Z,14Z Arachidonic acid**  **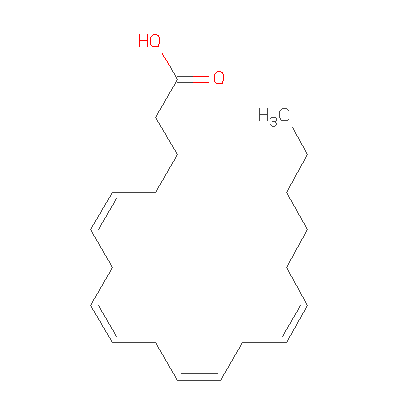** |
|  | **C20:5 Δ5Z,8Z,11Z,14Z,17Z**  **Eicosapentaenoic acid**  **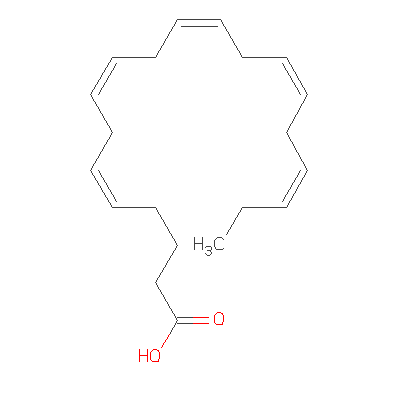** |

**Table S2. CYP5619 family members and their homologous protein sequences.** Each protein is presented with its assigned P450 family and subfamily names along with protein ID in parenthesis and respective species name.

>CYP5619A1(SDRG_14280.1)Saprolegnia diclina VS20

MGNLTSTGATHGDVHMDKMVMYLDGDRSAMMDGDLFVLEKALATEKVVGFCGPEALKVFDANLRDGTFVRHGALPSGLNE

LLGAVLPTTDGDAHARKKKLVLAAFSGAQLAAYKPLIRTTIQNEHAKWAAHGASMSLVANAKVLVFKLSLLLILGLEDNY

DNSRELLDTYMLALRNSVRRADPAGVRSRDELIRTMINPALATSHDRVHTGKPKPCALDHLVAAGVLSDDDLRAELFHLL

CMSLGGLECWVANCITAAASSTDVLAQLTAGRDAFITKYPAEADRWSHLGDLGYVNNYIQEVKRTYVAGPSHMYARATKD

TDVRTSEGTFHVPKGCLVAAALDGTNKHPSVWANPTKFDPSRFSTAKVDMAFGFCPHAIGADRRCAGEELSTLILQSFMV

SLFDFMWKMLPHQDYTLDTTLVNPMPKGGLMVVGFHRRTDLSASMVEVAGSEEDWKFLSLPEAKVYRDDKEALHDMFADE

RLDLWTHLMLKLLAKKQSMWNKPFANQAITAPKYQKTLPKITLYGLKIQIPTEDEDWPSDPWNEVATVKFLRDSCPLGDD

FEHTWLPGEDMERYVMSKVGSMWPRVNVHWNDRYSDRALELLVFNGLGQHLVTKLRTAHDDGSYYGICLDFMQALDVRPG

YAKYGADAYFNAKGKVTKIVRLGKTVHPGDEDWEYAKLCFRGSLQTKVTALDHLLGIHITVANGLVTSTREQLPPTHPLR

RLLKPFTFRSVIINYNASYALFWPKGMLHRAFSLSVEGMQQTWELGLANFKYETFPEHKARQNIDTTTLPYHEDGMDFWL

IVRGFVGSYIDLYYPCDESLTQDTAVQAFWSYLKTTLPPNSIRPLSKDNIKDFVAHAIFLVSSMHNHLGTIAEYVSDPAF

CPSAWVEGELAGRPGPCVRGALIMAATGFVQPSIKEDFSHIMLDDAAKAVCRKFTADVCAYAAVVEGRNTKRQHPYQAFN

PNTMEMAVSI

>CYP5619A1(XP_012203945.1)Saprolegnia parasitica CBS223.65

MGNLSSTGAMHGDVHMDKMVMYLDSDRSAMMDGDLFVLEKALATEKVVGFCGPEALKVFDANLRNGTFVRHGALPSGLNE

LLGPVLPTTDGDAHARKKKLVLAAFSGAQLAAYSPLIRTTIQKEHAKWAAHGASMSLVANAKVLTYKLSLLLILGLEDNY

DNSRELLDTYMLALRNSVRRADPTGVRSRDELIRTMINPALATSHDRVHAGTPKPCALDYLVAAGCLSDDDLRTELFHLL

CMSLGGLECWVANCITAAASSADVLAQLTAGRDAFMTKYPAEADRWSHLSDLGYVNSYIQEVKRTYVAGPSHMYARATKE

TDVHTSEGTFRVPKGCLVAAALDGTNKHPSVWANPTKFDPSRFSSTKVDMAFGFCPHAIGAVADRRCAGEELSTLILQSF

MVSLFDFMWKMLPHQDYTLDTTLVNPMPKGGLMVVGFHRRTDLSASMVEVAGSEEDWKFLSLPDAKVYRDDKETLHDMFA

DERLDLWTHLMLKLLGKKQSMWNRPFANQAITVPKYQKTLPKITLYGLKIQIPTEDEDWPADPWNEVAMVKFLRDSCPLG

DDFEHTWLPGEDMERYVMSKVGSMWPRVNVHWNDRYSDRALELLVFNGLGQHLVTKLPTAHDDGSYYGICLDFMQALDVR

PGYAKYGADAYFNAKGKVTKIIRLGKTVHPGDDDWEYAKLCFRGSLQTKVTALDHLLGIHITVANGLVTSTREQLPPTHP

LRRLLKPFTFRSVIINYNASYALFWPKGMLHRAFSLSVEGMQQTWELGLANFKYETFPEHKARQNIDTTTLPYHEDGMDF

WLIVRGFVGSYIDLYYPCDESLTQDTAVQAFWSYLKATLPPNSIRPLSKDNIKDFVAHAIFLVSSMHNHLGTIAEYVSDP

AFCPSAWVEGELAGRPGPCVRGALIMAATGFTQPSIKEDFSHIMLDENAKAVCRKFTADVCAYAAVVEARNSKRQHPYQA

FNPNTMEMAVSI

>CYP5619B2(OQR84828.1a)Achlya hypogyna

MGSAASSSSTGEVPCLRRAVSLKKMIMFMKDPRTAMMDCRDHYGDFFLVESWLTDEKVMGFCGPEALRAFDAKVAEGLIV

RGGSFAPGVLELLGDILPTIDGEAHATRRASIDTAFASEKIGMYKPKIREIVQREHASWAAHGGSISLAQNSRRMVFDVF

LAVLFGIEGSFDEHRDLLDTFVSAIRKSARKADAAGLEARRRIVEDLVRPAIRDARGRASVGEAKPVAIDALIADGKLGD

NELELELFHALFAGFSGVACLVVNSITAAIEWPEARARVFEARDAFFAKYPTEDDRWSAVELGYIDMYLLEVKRFYVAGP

TQIYGRAKEEVALTTAEGTFTIPKGCLATAGLETTNKHPGVWADPHVFNPDRFAADKTAVAKDGTIDVTADEMAYKFCPH

SIGSARRCLGEGLTSLVLQCAFVSLLDFVWQMVPDQSYALDEKSATPTPTGQLMAVGFRRRSPGTEHSVAGSEADWKFLR

CPEAEALVGGATGDLFGDARLDLWTRLMIKIIGKKQATWNCPPVNSLLTVPKHQTTLPKITLIQTEIEIPTEDEDWPHQS

WFEVQQSNFLRDHAPFIDDFVHKWLPGEDMERYVLSKVGHMWPRVNVHWNDRYSDRALELLAFNGLGQHLLQKLPEAHSD

GSYYGIELDFMQVLEVRPGYAKYGASAYFNQKGKVTKIVRGGSTFVPGDAGWEYAKLCFRGSLQTKVTAVDHLLGIHVTV

ANYMVTSAREQLAPAHPLRRLLKPPRAFRPKGMLQRAYALTTDGMKQTWEYGLSHFKYETFPEHRARQNIDTTTLPFHED

GMDYWNIVRTFVNDYLDLYFKTDTDVTGDVHVNKFWSFLNDKLPFD

>CYP5619A2(OQR84828.1b)Achlya hypogyna

MRPLTLENLKDFVAHGIFLVSSMHNHLGTIAEYV

SDPAFCPSAWVEGELAGRPGTAVRLALIMTATGFTQPAITEDFSHIMLDDAAKAVCHSFTKAVTDQIAVVDARNASRVQP

FQSFNPKTMEMAVTSYGDAHMDRMVMYLDADRGAMMDGDLFVLEKALATEKVVGFCGPEALKEFDAKVRDGSFVRQGALP

PGLLELLGPVLPTLDGTAHARKKAAVVAALSSSQLNKYKPLIRSVVQDEHARWAAHGASMSLVAYTKQLVFKLALLVLLG

LEDNYDHQREQLDTYMTALRNSTRRADPAGVTARAQLIAGLLNPALATAHDRVAAHAPKACVLDFLVGQGQLSDDDLRVE

LFHMLCMSLGGLECWATNCITAAASNPAVLKQLTAARDAFMTKHPTEDARWAHFQDLGYVNRYISEVKRVYVAGPSHLYA

RAAKTTDVHTSEGAFTVHAGVLVAAALDGTDKHPSVWPDPTKFNPDRFGAKVDMSYAFCPHAVGAVANRRCPGEELSTLV

LQSFLVSLFDFMWKMVPTQDYTLDTTLVNPMPKGGLMVVGFHRRTDLSASMVEVAGSEADWHFLSLPEASVYRNSSETLH

DVFADERLDVWTHLMLKLVAKKQSKWNRPFANSSITIPKYQKELPKITLFGLKIQVPTEDEDWPADPWVEVAMVKFLRDS

CPFVDNFTDTWLPGEDMERYVMSKVGHMWPRVNVHWNDRYSDRAFELLGFHGLGQHMLTKLPAAHADGSYYTIGLDFMQV

LEVRPGLAKYGADAFFDRNGKVTKIVRHGTTSRPGDDNWEYFKLCFRGSLQTKVTALDHLLGIHITVANQLVTSTREQLP

PTHPLRRILKPFTFRSVIINYNASYALFWPKGMLHRAYSLNEKGMQQTWDFGLANFKYETFPEHKARQNIDTLTLPYHED

GMDYWTIVRKFVSNYLDLYYKCDESLTQDTAVQAFWSYLKSTLPTGAVRPLNRENLKDFVAHAIFLVSSMHNHLGTIAEY

VSDPAFCPSSWVEGELAGRPGTGVRLALIMTATGFTQPAITEDFSHVMLDDAAKKIAKQFTADVTDFIAVVDKRNASRPQ

AYQSFNPKTMEMAVSI

>CYP5619A3(OQS07110.1)Thraustotheca clavata

MGNLTSTRPLGHDCANHKMLLDLGPEALKEFDAKLQNGSFVRQGAFPQGLLDLMGPILPTLDGAAHHAKKAAILEALNGV

QVEKYKPVIRSMVQKAHARWSAQGGAMSLVANCKQLAFKLMLVVLLGLENDYDDHRDDHRELLDTYILSLRDSTHRADPD

GVRSRQHLLDDMINPALQTSHERLNQNSLKPCVLDFLVSQKKLSDADLRIELFHLLTMGVGGLECWLANCITAAASSPDV

LKQLTVARDTYLKKYSNEEDRWRRFDDLGYVNWYIQEVKRVYIAGPSHVYARSTAQVDIVTSDGTFRVPKGALVAAALDT

TNKHPKVWTNPGEFNPNRFNKWDETKGMYTFCPHSVGSDRRCPGEQLSTVVLQSFMVSLFDFMWKMIPKQDYSLDTKAVN

PMPRGGLMVVGFHRRTAASDDMVQVAGSEADWKFLSLPEAKVYADSKETMYEMFSDERLDVWTHLMIQLLSKKQERWNRP

YANISIKVPQKQVKLKKVTLDGTKVEIPTEDEDWPSDPWFEVKTVEFLRDSCPMDDDFKYQWVPGEDKERYVMSKVGHMW

PRVLVHWNDRYSDRALELLAFNGMGQHLVQKLEKAHDDGSYYSITLEFMQGIEVRPGYATYGADAFFNSKGKVTKIIRKG

VTYRPKDDGWEYAKLCFRGSLNTRVTAVDHLLGIHLTVANYLVTSSREQLPPNHPLRRLIKPFTFRSVIVNFAASWGLIW

PRAMLQRAFAVSEKGIDTLWKTGLASFKYEPFPEHMERQKVDTISMPFHEDGLDYWYICHTFVSDYLNLYYANDEALTQD

TAVRAFWNFLNEKLPTGVRPLSLANLKDFITHAIVLVSAMHNHLGTLAEYVPDPAFCPSSWVEGEMAGRPGTSVRAALLM

AATGFTQPAITEDISGIMLDDKAKAVCKRFSEALTKQIDVVNERNKHRVQIYQSMNPAVMEMAVSI

>CYP5619B1(XP_012203946.1)Saprolegnia parasitica CBS223.65

MGSQTSTPAGAAPSLRRAASLKKMIMFMKDPRTAMMDCRDHYGDVFLMESSLVNEKIMGFCGPEALLAYDTQVQAGKIVR

AGAFPTGVLELLGSVIPTLDGDAHAKRKAALHVAFTPETLDTYKVKIREIIQHEHAAWAARGGSLSLALSCKKLVFHVFS

ATLLGLENVDDEYRELIETFVSSIRKSARKPDATGMDARTQVVEELIRPAIREAKARVAAEKPLPTVVDVLVADGRLSDE

ELGLELFHALFAGLGGVTCLAINSITVCIELPAIREKVSAAREAYLTKYPNEDDRWRHFADLGYMQHFLLEVKRFYVAGP

TQLYGRATDDLEISTADGSFKVPKGCLATAGLEATSKHPDVWSDPHTFNPDRFAPQDASAQPSGSVDPDELKDGARDVTA

PGLMYKFCPHSIGTARRCTGEGLTTLVLQCFVVSLFDFIWQMVPGQNYQLEEKSSTPTPVGQLMAVGFHRRALDRVVTFG

TAGSDEDWHFLSLPQARELVGCGAADLYDDARMDLWTRLMIKLIGKKQAAWDRPFVDSCLKIPKHQKVLPKLTLIQTSIE

IPTEDEDWPKQPWIEIKQSNFLRDHAPFIDDFTHTWLPGEDMERYVMSKLGHMWPRVNVHWNDRYSDRALELLAFNGFGQ

HLLMKLPEAHDDGSYYGICLDFMSVLEVRPGYAKYGADAYFNAKGKATKIVRGGVTSRPGEDGWEYAKLCFRGSLQTKVT

AVDHLLGIHATVANYMVTSIREQLPPAHPVRRLLKPFTFRSVAINFGAGRSLFWPKGMLQRAYALTDKGMKQTWEYGLAN

FKYETFPERKARQNIDTLTLPFHEDGIEYWQICRTFANDYINLYYKSEDAISADADLKRFWTFLDEKLPFAMRPLNLENL

KDFLAHGIFLVSSMHNHLGTIAEYVSDPAFCPSAWVEGELAGRPGTGVRLALIMTATGFTQPAITEDFSHIMLDDDAEAV

CQAFTAAVTAQIAVVDARNATRVQPFQSFNPKTMEMAVSI

>CYP5619B3(OQR84819.1)Achlya hypogyna

MGNDASVHAEGHAQALPSSNRATSLLKMIAFSKDPRAGMLDARDHYGDLFLLESKVVSEKIAGFCGPELLEAFDSKLAAG

EIVREGAFPAGILALLGPILSSLDGAAHTSRKAAVLEALSQAKLETYKPSIRAIVQTEHAAWAARGGAISLALLTRNLVF

RIFLQVLYGVEMLDDRHRVALDEFIASIRRSSKAPDPHGVSCRTRILEELIRPAIVKARARIAADAPAPCVLDNLITAAK

LDADALEVEAFHFLFAGFGGVACLATNVLTACATHPGVLPKLLEARAEFVTRYPTEDARFAHLDDLGYVNDFLLEVKRYY

VAGPTTVFGRAAVDLEVKTSNGVYHLPKGCLAAAGLEATNKHPDVWANPHDFNPDRFKDLDMASHAHRFCPHAFGEASHR

RCAGETLTTVILQTIVVSLFDFVWQMVPGQNYALQEGVATPTPVDQLMAVGFHRRTDDAVEFGVAGSQGDWKFLNLPEAK

ALVGGASDLYDDARLDLWTRLMIKLIGKKQAAWDRPYADQILSIPKFQKVLPKITLIQTNIEIATEDEDWPNQPWIEIQQ

SNFLRDHAPFVDNFNAKWVPGEDMERYVLSKVGHMWPRVNVHWNDRYSDRALELLAFNGLGQHLLQKLPEAHSDGSYYGI

ELDFMQVLEVRPGYAKYGASAYFNQKGKVTKIIRAGVTSHPGDKDWEYFKLAFRGSLQTKVTAVDHLLGIHATVANIMVI

ANREQLPPTHPLRRLIKPFTFRSVAINYGAGRALFWPKGMLQRAYALTSNGMKQTWEYGLSHFKYETFPERRARQNIDTT

TLPFHEDGMDYWNIVRTFVNDYLDLYFKTDANVGGDANVVQFWGFLRSKLPADAMRELTLENLKDFVAHFIFLVSSMHNH

LGTIAEYVSDPAFCPSAWVEGELAGRPGTAVRLALIMTATGFTQPAITEDFSHVMLDDAAKKVAKQFTKAVTEQIAVVDA

RNASRVQPFQSFNPKTMEMAVSI

>CYP5619C2(OQR84821.1)Achlya hypogyna

MGNSHSVQTSDAPPLPASKRSNSIFSLLNFAKNPNAAMAQGRDTLGDLFLLESAVLSEKIIGFCGPDMLAQYDGQVEAGG

IVRAGALPSGIVELLGPILPVLDGNVHAIRKKFVMAAFTEDQLTAYAPTIFSIVQNEHAAWAAHGGSISLGLLSKKLVFK

VFLAVLFGLTNIPPIEYDTKYDQYRDEVDGFIAGISKSATAPDAHAVACKQRLITELIGPAIVASQARVKAGAPRPCVLD

ALVAGDGLSDAQLRLEGLHMLFAGLGGVQCLVVNSLTVMAKFPDISEKLQEARAAFVTRCPTPADRWRHFDQLGYANQFL

LEVKRFYTAGPTQLFGRTATELTFQTPDGTYSVPKGALAVAGLNATNKHPEVWADPSVFNPDRFANFDTTTDLYTLCPHS

IGKMVGGRRCAGQDLATAVMQASLVSLFDFKWTFAPGQDFTLETGKSTPMPVGNIMVTAFQHRHEVGEDGCDVANWHLLN

MPEAKALAGVAAEVSEDEDDARLDLWTRLMIKLIGKKQSRWNKPVANEVLTIPKSQVTLPKITLIQTDIQVATEDEDWPN

QPWLEIQQSNFLRDYAPFVDNFEHTWLPGEDMERYVMSKVGKMWPRVNVHWNDRYSDRAVELIAFNGFGQHLLTKLPEAH

DDGSYYGIELNFMRTLEVRPGFAKYGANAYFNKKGKVTKIVRGGVTSRPGDATWEYAKLCFRGSLQTKITAVDHLLGIHA

TVANIMVIANREQLPPTHPLRRLIKPFTFRSVAINYGAGRALFWPKGMLQRAYALSTLGMKQTWDYGLSHFKYETFPERR

VRQNIDTVTLPFHEDGMDYWNIVRTFVSNYVDLYYKADSAIANDEHVRKFWSFLDDKLPFDMRPLTLENLKDFVAHGIFL

VSSMHNHLGTIAEYVSDPAFCPSAWVEGELAGRPGNAVRLALIMTATGFAQPAITEDFSQIMLDDAAKAVCKKFTADVTA

FIDVVDTRNLSRPQAYQSFNPKTMEMAVSI

>CYP5619C1(XP_012203939.1)Saprolegnia parasiticaCBS223.65

MGNQPSTEAGAAPLPDSKRANSIFSLLAFAKDPKAAMAESRDTLGNLFLIESAVVSEKIAGFCGPEMLSQYDAHVAAGHI

VRENALPAGIVELLGPILATLDGEVHDSRKEAIMGAFSKDMLASYAPIVFGIVQKEHAAWAAHGGKISLALSCKKTVFKV

FLAILYGITDMTPAEYDATYDPFRDLLDGFIRAIPKSSRGADAEGLVCKQRLLDELVAPALAASQARVAAKTPVPCFLDY

MLGQTELTPDVVHLEAFHALFAGLGGTQCLVVNTITALAQYPTVAEKVHASRAKFVTKYHEDRWRHFDNLGYCNRFLLEV

KRFYNAGPAQLFGRTTQELTFTTPDGEFAIPKGVLAVAGLDATNRHPDVWTDPSVFNPDRFDNGFNEATDLYKLCPHAIG

KTTGGRKCAGRDLATLVLQASLVSLFDFKWTLVPNQDLSLEEGKSTPMPKGLLMASAFTHRHSADETECDVSDWHLLNLP

EAKALVGIAGTVSDDEDDARLDLWTRLMIKLIAKKQARWNKPAANEVLTVPRFQRELPKMTLIQTNIQVATEDEDWPNQP

WLEIQQSNFLRDYAPLVDDFEHTWLPGEDMERYVMSKVGHMWPRVNVHWNDRYSDRALELLAFNGFGQHLLMKLPEAHDD

GSYYGICLGFMKGLEVRPGYAKYGADAYFNAEGKVTKIVRGDITARPGDDSWAYAKLCFRGSLQTKITAVDHLLGVHATV

ANIMVIANREQLPPTHPLRRLIKPFTFRSIAINYGAGRALFWPKGMLQRAYALTDKGMKQTWDIGLANFKYETFPEQIAR

QNIDTATLPFHEDGMDYWHICRSFVSNYVDLYFKSEDALQSDTDVHAFWTFLSTKLPVPMRTLTLENLKDFVAHFIFLVS

SMHNHLGTIAEYVSDPAFCPSAWVEGELAGRPGTGVRLALIMTATGFAQPAITEDFSHIMLDDDAKAVCQAFTAAVTAQI

AVVDARNATRVQPFQSFNPKTMEMAVSM

>CYP5851A1(OQS03666.1)Thraustotheca clavata

MWNCFSGSGDEAFPSGKVPYITDEQVQHMHGVIYLLEYAVGEENVAVLKGSNLIQQFDTHRQNGNLSRQDALPIGLVDLA

GKTLSTLDNATFAKRQSALLDAFSIEQVAKYQSKIDAIVQSRHSAWAARGGSFSIAVETKKLVFHIFVGVILGLEDQYDA

VFNLVNQYRELLPKSLRRPHAKAITLRQEILSKLITPAVTSSRTRVANKQTNDSVVDYLIRKGQLSDADITIELFQALID

GTDGISSLVINCVNAWVNQPGLSDKLASVRDAPDTFVNQFIDEVERVYTAGPSHEYARVVKNTTFTTPKGSFTLPKGQLV

VAFTESINEDASVWPNPTLFDPSRFENDTPDPYKFTAFSLLQLVNRVQNVREAFTKAVLRSNMSSLLNCMWQMVPLQSYE

LTEHTVTNPTPVGQLTVVNFHKRHEQSANSVATAGTPEDWKFLEQPEAKQYADCSESLDELFADKRLDVWTNLTLKLLER

KQAKWNRPFANSAITVPKYQAELPKIQLYGTNINVPTEDEDWPSNPWIEVKTVEFLRDSCPVNDNFDDMWLPGEDMESYV

MSKVGKIWPRVNVHWNDRYSDRALELLAFNGLGQHMLEKLPDAHDDSSYYGIFMNYMDGLDVRPGYAKYGADAYFDRDGI

ITKIVRQGVTYQPKDAGWEYAKLCFRGSLITKVTAVDHLLGIHVTVANSLVTSSREQLPPNHPLRRLIKPFTFRTVIINH

AASYALFWPKGMLHRAFALSLDGMQQTWEFGLANFKYETFPEHKARQNIDTATLPYHEDGIDYWNIVHNFVSEYLDLYYK

SDDSLLQDDSVVAFWEYLKSTLPKDSIRPLTLVNLKDFIAHSIFLVSSMHNHLGTIAEYVPDPAFCPSAWVEGELAGRPG

TSVRASLIMGATGFAQPSIKEDFSHIMLDDEAKAICKKFTAEVSAFSAVVIDRNTRRKQPYQSFNPDTMEMAVSI

>CYP5619C3(OQR84833.1a)Achlya Hypogyna

MGNQPSTETGAPPLPDTKRANSIFSMLAFAKNPREAMAESRDTLGNLFLIESAIVSEKIVGFCGPEMLAQYDAQVEAGGI

VRDGAFPAGIAELLGPILPALDGEIHAARKAAVMTAFSKEQLALYVPLIFGITQKEHAAWAAHGGAISLALLSKKLVFKV

FLAVLYGIETDTPAEYEAKYDHFRDVVDGYIHAIPKSAKAPDADGLRYKARAIDELIAPALAASQARIEAGTPRPCVLDY

WVQHSGMQPDDICLEAFHALFAGLGGVQCLVVNTITAMATNPGAAEKLHHTRAEYVLKYHSAEDRSSHFDQLGYANQFLL

EVKRFYMAGPSQLFGRTTAELTFQTPDGTYSVPKGALAVAGLNATNKHPEVWADPRVFNPDRFADFDADADLYKLCPHAI

GKTNGGRRCAGQDLATAVMQASLVSLFDFKWTFVPGQDFTLETGKSTPMPVGNIMVTAFQHRHEVGEDGCDVANWHLLNM

PEAKALAGVAAQVSDDEDDARLDLWTRLMIKLIGKKQSRWNKPVANEVLTIPKSQVTLPKITLIQTDIQVATEDEDWPNQ

PWLEIQQSNFLRDYAPFVDNFEHTWLPGEDMERYVMSKLGHMWPRVNVHWNDRYSDRALELLAFHGFGQHLLQKLPESHD

DGSYYGIELDFMRTLEVRPGFAKYGADAYFNENGKVTKIVRGGVTSRPGDATWEYAKLCFRGSLQTKITAVDHLLGVHAT

VANIMVMANREQLPPTHPLRRLIKPFTFRSIAINYGAGRALFWPKGMLQRAYALTDKGMKQTWDFGLSHFKYETFPEHIA

RQNIDTTTLPFHEDGMDYWTIVRTFVSNYVDLYYKAEADVENDADLHAFWSYIGSMLPVPMRKLTLENLKDFVAHFIFLV

SSMHNHLGTIAEYVSDPAFCPSSWVEGELAGRPGTAVRLALIMTATGFAQPAITEDFSHIMLDDAAKAVCLAFTKAVTDQ

IAVVDARNASRVQPFQSFNPN

>CYP5852A1(OQR84833.1b)Achlya hypogyna

MTSRNKVMALPDNASVSLRDLVADATAMAELRLLLSNQKLMGAYGARLLAVLEEQAYIA

QQPAVQRRPPPATEWTGGLHKLQTFAKDPCGTSLALHAKYGDIFFLDSVWSSTMIAGVAGPSLLMAFDDHWNAGRLGSAV

PSGVLPLLGPVLPTLDGSKHRARKAALLAGVVPATHAVVIANMVADELEAWAAAECTFSFVVRAQSLALKLLLRILLGIT

GASPLLLGNCQHWIDTLVAAVPASTVAPVPQGLLAKERLLADLCRPAVAASRTRFASKAAVTCVLDALVERNELPDEVLA

LELLHCLCTGVAPLGSLLANTVTASHKFPAVWSKLQRSASAYTQSKIDGALWEYGSHFAMEVQRFYSAGSSLRYGRAKTD

LIFTAGEVVYTLPKDSLVVAGVRATHVRAASWAVPHHFNPDRFAAGVEKGAWQPLRLGGFCDALSTRIVEAWALALTNYS

WHLVPGQDFAVDRAVPSSVPAGKLVASHFRRIRPAVAASPAQCLALPSASEYTQLIAVAGDVVSKHDPRLDFWTREMYKL

VVLKLSRWSRPEASKALTIPATHGPIDKITLVQTSIQVPLEDEDWPNQPWIEIKFANGLRDYAPFVDNFAADWLPGEDKE

RYVMQRFGHIWPRVQVHWDDRYSDRALELIAFNGLGQHMITKLPEAHTDGSYYSVATNFMYGLEVRPGFAKYGADAYFDS

NGKVTKIVRGSLTFRPDDPDWEYAKLCFRGSLQIKVTALDHLLLVHSTVANHVTVLHREQLPPAHPLRRLIKPFTFRSAA

INFSAGRALFAPKGMLQRTVALTTAGMKQAWDYGLASFAYEPFPAMIARQNIDTVSLPFHEDGMDYWCIVEQFVDAYIAL

YFHTDVDVTGDDAIVGFWAALNATMPYDLPALSLAALKEFITYFVFTVSSMHNHIGAIAEYVSDPAFCPAAWVEGELAGR

PGTAIRLALLMIVTGFDQPQITEDFSHVMLDDDAKAVARSFTRAVTEQIAVVDARNQKRVQPFQSFNPSTMEMAVGI

>CYP5619B4(XP_012203942.1)Saprolegnia parasitica CBS223.65

MGNEASTVHADGAATDLPASQRAMNILKMIEFSKDPRAGMLESRDQYGDLFLLESHLVSEKIAGFCGPELLAAFDDKLRD

GSIVREGAFPPGIVALLGAIMPTIDGEEHHARKAAALEAFTPARLDLYAPLVREIVQAEHASWAARGGAISLACLTREMV

FRIFLKVLYGVERHDANKFRKRPDPHGVSCRTQILDELIRPAIADARARAATKTPAPSVIDCLVTNGKMASDVLETEAFH

FLFAGFGGVACLATNILTAVATHPSARKDLLDARAEYVTKYDGEARWAHFHDLGYVNLFILEVKRFYVAGPTAVFGRAKT

DLEIPTKNGVYKLPKGCLAAAGLEATNRHPDVWTDPNLFNPNRFRDLGHVRTTKPHAFCPHAFGALSHRRCAGEDLTTLI

LQSTIVSLFDFVWQMVPNQDYKLAVGVSTPTPVGQLMAVGFHRRTDDAAEIIGTVGSNADWKFLNLPESKELALWDRPYA

NQILSIPQHQKTLPKITLIQTHIEIATEDEDWPSQPWIEIQQSNFLRDYAPFVDNFELTWLPGEDMERYVMSKVGHMWPR

VNVHWNDRYSDRALELLAFNGFGQHLLMKLPEAHDDGSYYGICLGFVKGLEVRPGYAKYGADVYFTAKGNVTKIVRGDIT

SRPGDAGWEYAKLCFRGSLQTKVTAVDHLLGIHATVANIMVIANREKLPPTHPLRRLIKPFTFRSVAINYGAGRALFWPK

GMLQRAYALTDKGMKQTWDFGLANFKYETFPEHKARQNIDTTTLPFHEDGMDYWQICRSFVSNYVDLYFKSEDALQNDTD

NLKDFVAHFIFLVSSMHNHLGTIAEYVSDPAFCPSAWVEGELAGRPGTGVRLALIMTATGFAQPAITEDFSHIMLDDAGK

AVCQAFTAAVTAQIAVVDARNATRVQPYQSFNPKTMEMAVTVAVDATGSSKRR

>CYP5619F1(XP_008879406.1)Aphanomyces invadans

MGASASSFVAKESTSLSTLIAFSKDPRTALLSARDHFGDIFLVESAFVTTRIAGLCGPEALQQFEEKMLEGALVREGAFP

PSILALLGPILVTMDGEVHRTKKNALLRAVSPVQLDVYKPIIRRIIQAEHSKWAAHGGAISFALNTKILVFKILLAVLYG

MEGEFDSFRTYIEDYVAAIKQSAKTTSAHGVTCRATFIAEILEPAIAAAKARQQVNATSSGPLESVLDVLVASGELNDDD

LKNEGFHIMFAGFGGLSAAATNLITAAVVFPEIRAQVFAARDKYLSKFGDDRWGHLDDLGYLNKYILEVKRFFLAGPTQV

YAKAARDVDLVTSKGVFHLSKGSLVMAGLEATNHDPDVWAAPNTFDPSRFNDADIDAAHGVKNTRKYSFCPHGFGDVRNR

RCAGEELSTIVMQSLLVSYFDFTWKMVPGQNYTLQPHSVTAVPIGLLMAMGFQRQQDDGSGNLDYGVVGSHADWKFLRRP

DVQELTGHNAAEYFDDSRLDLWTRLMIKLISKKQSVWNRPYAQSALSLPMEQVVLDKITLIQTQIEIPTVDEDWPSQPWL

EIQQSNLLRDHAPFVDDFDHPWLPAEDGERYVMSKVGHMWPRVNVHWNDRYSDRALELLVFHGLGSHLVQKLPQEHDDGS

YYGLLLNVMQGLEVRPGFAKYGADAFFDKHGHVVKIKRGDQTYTKTDAAWEYVKMCFRGSLQTKVTAVDHLLGVHATAAN

YLVTSSREKLPVNHPLRRLIKPFVFRSVAINYSAGRALFWPNGMLQRAYALTTAGMKSTWEFGLSQFEYATFPDRIARQQ

IDTLTIPFHEDGLDYWNIMIKFVSSYVDLYYPDDASIQHDDDVVAFWSNLTAVSPAPLPDLNKSNLKDFLAEGFFLVSSM

HNHLGTIAEYVSDPAFCPSAWVEGELSARPGNAVRLALIMTATGFTQPSITEDFSHVMLDDAAKAIVRTFTTDVKAQIKV

VDARNATRVQPFQSFNPKTMEMAVSI

>CYP5619G1(XP_008878127.1)Aphanomyces invadans

MGNVTGHVQREREYVKTVIGFMKDPRTFMSASRNTYGDVFLFQSSLVNQKIAGLSGPEALQAFEARLADGSLVKTGALPS

GVSDLLGPIMSVLDGEDHHRKKAGIMTAFTPQQLAKYLLVVRRIIQTEHARWAARGGVISITASSKELVFKLLLAVLYGI

EGDFDEYRPLVDEFVASIRKSAVKASPEGKAARDTIMNDLVIPAIEAAKVRVAGGTPSPSALDHLVGLNQLADDDLGVEM

FHVLFAGFGGLSCLATNLVTPLVTMPDVREKILDARDQFLSKYTGDTKWDHLEDLGYINQYILEVKRFFVAGPTQSFAKA

AVAFDVVTSKGTFHIPKGCLVAAGLETTAFDAEVWPNPDNFDPSRFDNNDDLSALQFKLCPHGIGSTSNRRCAGETLTTL

VCQALVVSLFDFTWNMVPGQDYELDENTSIPTPRGGLKAVGFRRRDAVTSYGVAGTDDDWTFLKLPEAKAIVSVHGGWGD

SDGLFADPRLDLWTELMIKLIGKKQAKWNRPYADTALMLPKNKQPLVKLTLAQTSIQVPTEDEDWPTQSWVEVKQANFLR

DHAPFKDDFVHKFLPGEDGERYVMSKVGHMWPRVNVHWNDRYSDRALELLVFNGLGSHLVQKLPTEDPTDGSYYGVLLNF

MQVLDVRPGFAKYGADAFFDKQGKLIKIIRGDKTYTKTDVEWEYVKMCFRGSLQTKVTAVDHLLGIHVTVANYLVTASRE

QLAVNHPLRRLFKPFTFRTVSINFSAGRALFWPNGMLQRAYALTNSGMKQTWEYGLSHFVYAPFPDRVKAQQIDTFTLPF

HQDGLDYWAIVFSFVSKYIDLYFADDAAIAGDTDVVNFWTYVTSVSPVPLPPVSKASLKDFIAQGIFLVSSMHNHLGTIA

EYVSDPAFCPSAWVEGDHAAPPGNAVRLALIMTATGFTQPAITEDFSHVMLDNAAKDLVRTFTADLFKLIDVIDARNTTR

VQPFQSFNPKTMEMAVSI

>CYP5619F2(XP_009834503.1)Aphanomyces Astaci

MGAAASNYVYNEATSLSTLIGFSKDPRTALLNARDHYGDIFLVESAFVSTKIAGLCGPEALKEFEAKLQDGSLVKQGAFP

PSILALLGPILVTLDGDVHHAKKAALLKALSPAQLDVYKPIIRRIVQTEHSKWAAHGGAISFAVNTKILVFKVLLAVLYG

VEGEFDTYRRYVDDYVTAIKQSAKVTDEHGVTCRAKFIAEIIAPAIAAAKANQTKRQQQPLNSVLDVLVATGDLTDDDDL

QNEMFHFMFAGFGGVSAAATNLITAVCVFPDIRAKVLRARDDFLRQYDGRDESPWNHLDEMGYLNLFVLEVKRYFVAGPT

QVYAKAARDLDLVTSTGVFRIPEGALVMAGLEATNRDPDTWPSPDSFDPTRFTQADVDGMHMTRPFSFCPHGFGSHRRCA

GEQLTTVIMQSVLVSLFDFTWKMIPGQEYALQPHSVTAVPIGQLMGVNFHRRLNEDDPSTPEVETYGIVGTQDDWKFLRR

PDVQELTGVNAAEYFDDSRLDLWTRLMIQLISKKQTLWNRPYATTALSVPQHQQVLDKITLIQTNIQIPIVDEDWPCQPW

LEIQQTNLLRDHAPFVDDFSHLWLPAEDGERYVMSKVGHMWPRVNVHWNDRYSDRALELLVFHGLGSHLVQKLPQAHADG

SYYGVLLNVMQGLEVRPGFAKYGADAFFNKHGKLVKIQRGDKTYTNTHDDWAYIKMTFRGTLMTKVTAVDHLLGVHVTAA

NYLVTASREKLPVRHPLRRLLKPFTFRSVSINYGAGRALFWPNGMLQRAFALTTAGMKQTWEFGLTQFEYATFPETMAKQ

EIDTLTLPFHQDGLDYWHIVYKFVANYVDLYYPSDDDVAMDVDVGKFWRYMGELSPAPLPDLTKSHLKDFMSQGIFLVSS

MHNHLGTIAEYVSDPAFCPSAWVEGELSARPGNAVRLALIMSATGFTQPSITEDFSHIMLDDKAKALVKTFTADLYAQIK

VVDARNANRVQPFQSFNPKAMEMAVSI

>CYP5619D1(XP_012194083.1)Saprolegnia parasitica CBS223.65

MVSIPLLLITIVGQAAGAPQGLGSVLQGVINDVKHSVAGVRYAFESLVDAEPVTGFCSPEALRAFDDALISGALERRTAY

PAGILELSGPTLSTIDGPAFSKRQDAFLNALSGPALAAYQPRIQRRIQEDHAMWAARGSTFSLALHAKTSVFKVFLDVVY

GVSDPEKYTGYRAQLDEYLFYVSKTSRRAPADATKIRERLLAAIVRPAIASSVARVRSGASTTCVLDAVVAQGSVSEADL

VVESFQLLAMGLPGLEGLVVHTITAMVSHDDVRGQMATARDAYTAQYPNGAHWSHLEDLDAVNQYVNEVQRVYGASPRHT

FARATKDLTVPDGSGAMVAVPKNRLTVALLDCINHDPKRWPSPEQFQPARFATANTSAYGFAPFAIDDLVHRVEGRREGL

SRLILQSHVVSLLDFVAVMAPLQSFALGDGVNPLPIDLLTTVSFRYAPGVVQQDVDAWRRLHHPNAKLYNGSLENPLLAA

SDKRLDFWTHSMIQLFNVRFETWVTPTAAASIKVPTTQKTLPKRTLYGTSIQIPTEDEDVAIPKVVLESAKLLQDSAPFV

DNFDAKWAPGEDMEGYVLSKVGRMWPRVRVHWDDRYSDRALELLVFHGLGQHMVQKLATAHDDGSYYTVATNFLASIEVR

AGYAITGADAFFDAKGKVTKIVRLGKTIRPTDAAWEYAKMCFRSSLVSKITAVDHLMGLHVTVGNYMTTASREQLPPAHP

LRRLIKPFTFRAVAINYDASIALFAPKGMLHRAFPFTEKGLKDTWAMALKSLTLEPFPVHLARQQVDTITLPYHEDGADY

WKIVRTFVSEYLDLYYKSDDDVTRDASIQALWAFLNKQLPTPLGVLSLENLKDVVAHSIFLVTAMHNHLGGIAEYVSDPA

FCPVSWVEGELSGRPGNAVRAALIMSGTGFPQPNILEDFSHVLLDDAAKAVAHRFTASLQAFVQVVEARNAQRILPYQAF

NPLVMDMAIGI

>CYP5619D3(AIG56338.1)Achlya hypogyna

MVAVSWLWCFGPTLVAAEPQGLGSFFQGIISDIKHAVNDLTFRFEHLVDAEPVTGFCGPDALRAFDNYLATGALVRHDAY

PKGVLDLVGSTLATLDGSAFATRQAAFLNALSPAAVQRYKSTVHNIVQADHATWAARGGTFSLANAAKVTTFKVVLAVVL

GLDNPEAYTGYRSQIDEYLALLAQTEWRAPADAVTIRSRLLAALIRPAVVAAHARATPKSCVVDALVEAGTVSDEDLATE

LFQLLVHGIPGLEGLVVHSLTAIASVDGVRAHLASARDVYMAKYYGAARWDHFDDLGYGNQFLLEVQRTYTASPRQEYAR

ATVDLKVLTPTGTTIVPKNRLKVGVLECLNKDAKRWPNPTSFDPTRFASANTSAYAFAPYAMNLLADRRRGVGEALSQLV

LQTHLVSLWDFAWTMAPRQSYALADSPNPSPVDALTTDGFFVAPGAVVDTEAWRRLHQPDVQLYNASIENPLLAAGDKRL

DFFTHSAIQLFNTRYNLWVKPSASAITVPKVQKVLPKRKLYGTAIQIPTEDEDVDIPKALLEAAKLIQDTAPFVDNFDAK

WLPGEDMEDYVLSKVGHMWPRVRVHWDDRYSDRALELLVFHGLGQHLVTKLPHAHDDGSYYTVALDFLGALEVRSGFAKL

GADGFFTKDGKVTKIVRQGVTYLPGAAKWEYAKLCFRGSLNAKITAVDHLIGLHVTVGNYMTTATREQLPPKHPVRRLLK

PFTFRAVAINYEASNVLFAPKGLLQRAFPLTEKGMAQTWVTALKDLKLETFPQHIARQQVDTMTLPFHHDGTDYWNIVRR

FTSNYLDLYYKDDTAVTSDASLQSFWRTLSAQLPMPLPPLGLAVLKDTTAIGIFLVTAMHNHLGGIAEYVSDPAFCPTAW

VEGEIAGRPGSCVCAAVLMAGTGYLQPNVMEDFSHVLLDDAAKAVARNFTTSLQAFTDIVRSRNAQRLLAYRAFDATIMD

MAIGI

>CYP5619D4(AIG56100.1)Achlya hypogyna

MVSITRLLHLSLAAATVAGAPQGFFQDLISDIRHGIAETLFGLEQLVAAEPVVGFCSPEAIRAFDELIAAGALQRQSAYP

KGVQNLVGSTTLTLDGPAFAARQAALLAALSPMAVQTYAPTIRAIVQADHATWAARGGLFSLDDAARTMTFKVFVAVVLG

LESPERYTGYRAQLDDYLSYLRVTASVAPPEAVAIRKRLLDTLVRPAITAARARSTPKPSVVDILVAMGSVADADLADEI

FALLANGLPGLEGLVVHTLSTMASVEGVVANLATARDVYLAKYPGAARWQHLDELGYANQFLLEVQRTYGAKPSHVFARA

TKELSVAGVKVPKNRLTAVLLECLNQDPGRWPEPARFDPSRFAVANTSAYEFAPFAMNLLTDRPHGIREALTTTVLQTHV

VSLFDFVWSMAPHQNYTVEAGVNAGPVDGLMTVSFRAAPGAVVDTEAWRRLTRPYPEAFNSSLDNPLAADPRLDFLTHSL

IQLGNTRFTLWVKPSAATAITIPTTQGVLPKRTLYGTTIQIPTVDEDVKIPKELLEAVKLLQDTAPFVDNFDATWRPGED

MEAYVLSKVGRMWPQVRVHWDDRYSDRALELLVFHGIGQHMVTKLPQPHADGSYYTVALNFMDALEVRAGYAKAGADAFF

TSKGKVTKIVRQGVTYVPGDAGWEYAKLCFRGSVIIKITAVDHLIGLHVTAGNYLTTASREQLPPAHPLRRLLKPFTFRA

AAINYDASSALFAPKGILHRAFALSEKGMAQTWAAAQTMIRLETFPQHIARQGVDSLSLPFHEDGLAYWDIVHSFASDYL

GLYFPSDAAVTGDASVVAFWKALAAVTPLPALSRTALVDATATAIFLVTAMHNHLGGIAEYVSDPAFCPAAWVEGELAGR

PGTSVRSAIIMSGTGYLQPNVMEDFTHVLLDDKAKAVARRFTAALRGLVGVVQSRNAKRVLPYRGFDPEIIDMAIGI

>CYP5619D5(AIG56283.1)Achlya hypogyna

MVSITRLLRLSLAAATVAGAPQGFFQDLISDIRHGIAETLFGLEQLVAAEPVVGFCSPEAIRAFDELIAAGALQRQSAYR

KGVQNLVGSTTLTLDGPAFAARQAALLAALSPAAVQTYAPTIRAIVQADHATWAARGGLFSLADAARTMTFKVFVAVVLG

LESPERYTGYRAQLDDYLSYLRVTASVAPPEAVAIRKRLLDTLVRPAITAARARSTPKPSVVDSLVAMGSVAEADLADEI

FALLANGLPGLEGLVVHTLTTMASVEGVVANLATARDVYLAKYPGAARWQHLDELGYANQFLLEVQRTYGAKPSHAFARA

TKELSVAGAKVPKNRLTAVLLECLNQDPGRWPEPGRFDPSRFAIANTSAYAFAPFAMNLLTDRPHGMREALTTTVLQTHV

VSLFDFVWSMAPHQNYTVEAGVNAGPVDGLMTVGFRAAPGAVVDTEAWRRLTRPYPEAFNSSLDNPLAADPRLDFLTHSL

IQLINTRFNLWVKPSAATAITIPTTQGVLPKRTLYGTTIEIPTVDEDVTIPKALLEAGKLLQDTAPFVDNFDAKWRPGED

MEAYVLSKVGHMWPQVRVHWDDRYSDRALELLVFQGLGQHMVTKLPQPHADGSYYTVALNFMDALEVRAGYAKAGADAFF

TSKGKVTKIVRQGVTYVPGDAGWEYAKLCFRGSVNIKITAVDHLIGLHVTAGNYLTTASREQLPPAHPLRRLLKPFTFRA

AAINYEASNSLFAPKSVLHRAFAFSEKGMAQAWAAAQSMIRLETFPQHIARQGVDSLSLPFHEDGLAYWDIVHSFASDYL

GLYFPSDAAVTGDASVVAFWKALAAVTPLPALSRTALVDATATAIFLVTAMHNHLGGIAEYASDPAFCPTAWVEGELAGR

PGTSVRSAIIMAGTGYLQPNVMEDFTHVLLDDKAKAVARRFTAALRSLVGVVQSRNAKRVLPYRGFDPEIIDMAIGI

>CYP5852B1(XP_012203940.1)Saprolegnia parasitica CBS223.65

MGNQPSGRTKVMALPPPDAKLHDLATDPVIMADLRKLLSNRSIAGAYGPLLLAAIEEHVGATPQPVAMVQRRPAPSTEWS

GGLSKLRAFAAAPVASFEALHATYGDLFYIESVWTSDKIAGVAGPTLVAAFEDHMDACRLARSVPSGVTHLLGPVLATLN

GPSYKARWTNLASAFAPGHQFEPVVQRLFRDELAAAHAAGRTFSFTVLAQHLVLKLLLSLLLGVTASSQLELANVQHWID

TMVAALPRSTVAPHNDALQAKEQLLATLLQPALLASRRRVDAKAPVACVLDNLVLKNDLSDDVILLELLHALYTGAGPLA

ALLANTISASHAYPAVWAKLVADTRAHKQQSPGAWKFGRAFAKEIQRFYRVGSGLRFARATSDITFAVNDVVYTVPKHTV

VVAGIDATHKHAASWSAPADFIPNRFLDDAESTKNALHLFQLGGVSDALPTMVLESWLLAVADYTWFLTPGQETSLDKAS

VTSPLPVGKLIASHMERRVGVSAIPDASALVRLPTTDEYAALIAVANEQLLTRDPRLDFWTHQMYKLVLIKLSRWTRPEA

AKALTIPATMGPVDKMTLAQTNIQVPLDDEDWPNQPWIEIKFANAIRDYAPFIDNFDENWLPGEDKERYVMRFYAHIWPR

IQVHWNDRYSDRALELMAFNGLGQHMLQKLPTTHSDGSYYTIATNFMQALDVRKGYAKYGADVFFDDKCKVTKIVRGNIT

YRPDDAEWEYVKMCFRGSLQTKVTAIDHLLLIHSTIANHVTVVHREQLPPTHPLRRLLKPFTFRSAAINYGAGRALFWPQ

GMLQRAIALTTRGMKQAWDIGLGSFGYETFPALVERQQIDTTTLPLHEDGIDYWHIVSRFVSSYLDLYYAADAEVTADAS

VVAFWTMLDATLPFALPPLSLKSLHEFVTYFIFMVSSMHNHVGAIAEYVSDPAFCPSAWVEGELAGRPGTSVRLALIMII

TGFDQPQITEDFSHVMLDDAAKCVARAFTTDVKAQIPVVNRRNATRVQSFQSFNPSTMEMAVGI

>CYP5851A2(OQS07119.1)Thraustotheca clavata

KDCVVDYLVQQAQITDADITIELFQALIDGTDGISSLIINCVTAWVKQPGMSDKLASIRDSPDAFVDQFINEVERVYTAG

PNHEYARVLTKTTFTTPKSSFSLTKGQLVVVFTESINEDVTVWSNPTSFNPSRFENGTPEPYKFTSFNLLQLVNRAQGVR

EEFTKAVLRSNMLSLLTCMWQMVPLQSYELSEHTVSNPTPVGQLMAVSYHKRHGLSANSVTTAGNPQDWKFLEQPEAKEY

RQSVESLGEAFEDCRLDFWTHAMIQAVKNRSLVWRQPTARAEITLPKYQKVLEKVTLSGTNIEVPVEDEDTGNDLNFAQA

HTFNLLRDLAPLIDNMDATWLPGEDMEGYVMGKVGMMWPRVNVHWNDRYSDRALELIAFNGVGQHLLTKLPEAHEDGSYY

TIALEFMYGLAVREGFANYGGDAFFTQEGKVVKIVYGGEEYLSDNEQWEHIKMAFRGSLLARVTALDHLLGTHVTVANYL

TTASREQLPPDHPLRRLIKPFTFRSVAINYSAASVLFWPKGMVDRAFAFTHESLENVWAYGLKHFSYEPFPEFVANQKID

TVELPFHQDGMDYWTICHAFVSKYVDLYFSSEEQLIRDAAVASFWQFLVEKVPVPKFPTLSLDNLKNFLAHGIFLVSAIH

NHVGSIAEYVSDPAFCPSAWVKGELAGRPPTCVRAALIMAAAGLPQPSILEDFSHVMLDDDAKSICQDFTAALVKHQNVV

DERNAKRVQPFQSFNPKMMEIAVSI

>CYP5619B1(SDRG_14281.1)Saprolegnia diclina VS20

MGSQASTPAGAAPSLRRAASLKKMIMFMKDPRTAMMDCRDHYGDVFLMESSLVNEKIMGFCGPEALLAYDTQVKEGKIVR

ASAFPTGILELLGAVVSTLDDDAHAKRKAALLVAFTPEKLDAYKPKIREIIQHDHAAWAARGGSLSLALSCKKMVFHVFM

ATLLGLENVDDEYRELVEAFVSSIRKSARKPDTTGMDARTQVVEELIRPAVREAKARVAAQKPLPTVVEVLVADGRLSDE

ELNLELFHALFAGLGGVTCLVINAVTACIELPAIREKVSAAREAFLAKYPNEDDRWSHFADLGYMHHFILEVKRFYVAGP

TQLYGRATDDLEISTANGSFKVPKGCLATAGLEVTSKHPDVWSDPHTFNPDRFAPQDASTTPVDPDAFKDGARDVTAPDM

MYKFCPHSIGIARRCAGEGLTTLVLQCFVVSLFDFIWQMVPGQNYQLEEKSSTPTPIGQLMAVGFHRRTLDDVVTFGTAG

SDEDWHFLSLPQAKELVGSGTADLYDDARMDLWTRLMIKLIGKKQATWDRPFVESCLTIPKHQKVLPKLTLIQTSIEIPT

EDEDWPKQPWLEIKQSNFLRDHAPFIDDFKHTWLPGEDMERYVMSKLGHMWPRVNVHWNDRYSDRALELLAFNGLGQHLL

MKLPEAHDDGSYYGICLDFMNVLEVRPGYAKYGADAYFTAKGKVTKIIRGGVTSRPGEDGWEYAKLCFRGSLQTKVTAVD

HLLGIHATVANYMVTSIREQLPPAHPVRRLLKPFTFRSVAINFGAGRSLFWPKGMLQRAYALTDKGMKQTWEYGLANFKY

ETFPERKARQSIDTVTLPFHEDGIEYWQICRTFANDYVDLYYKSEDATSADADLKRFWTFLDEKLPFTMRPLNLENLKDF

LAHGIFLVSSMHNHLGTIAEYVSDPAFCPSAWVEGELAGRPGTGVRLALIMTATGFTQPDITEDFSHLMLDDAAKAVCKA

FTAAVIAQIAVVDARNATRVQPFQSFNPKTMEMAVSI

>CYP5619B2(SDRG_14277.1)Saprolegnia diclina VS20

MGNEASTVHADGAATDLPASHRAMNILKMIEFSKDPRAGMLESRDQFGDLFLLESHLVSEKIAGFCGPELLAAFDDKLRD

GSIVREGAFPPGVLALLGPIMSTIDGEEHDARKAAALEALTPARLDLYAPIIREIVEAEHASWAARGGAISLACLTRDMV

FRIFLKVLYGVERHDGNKFRVLLDDFIVSIRRSSKHADPHGVRCRTQILDELIRPAIANAQARASNKTPVPSVIDCLVAN

GKMTPDVLETEAFHFLFAGFGGVACLATNILTAVATHPSARKDLLDARAEYVTKYDGDARWAHFHDLGYVNLFILEVKRF

YVAGPTAVFGRTKTDLEIPTKNGVYKLPKGCLAAAGLEATNRHPDVWTDPNLFNPNRFRDLGHVRTTKPHAFCPHAFGES

SHRRCAGEDLTTLILQSTVVSLYDFVWQMVPNQDYKLAVGSSTPTPVGQLMAVGFHRRTDDAVEIIGTVGSKADWKFLNL

PEAKELVGTAMDLYDDARLDLWTRLMIKLIGKKQAVWDRPYANQILRIPQHQKPLPKITLIQTNIDIATEDEDWPNQPWL

EIQQSNFLRDHAPFVDNFEHTWLPGEDMERYVMSKVGSMWPRVNVHWNDRYSDRALELLAFNGFGQHLLTKLPEAHDDGS

YYGICLNFMKSLEVRPGYAKYGADAFFTSKGKVTKIIRGDIASRPGDSGWEYAKLCFRGSLQTKVTAVDHLLGIHATVAN

IMVVANREQLPPTHPLRRLIKPFTFRSVAINYGAGRALFWPKGMLQRAYALTDKGMKQTTQDAPAHRHNDAAVP

>CYP5619C1(SDRG_14273.1)Saprolegnia diclina VS20

MGNQPSTEAGVAPLPDSKRANSIFSLLAFAKDPKAAMAESRDTLGNLFLIESAVVSEKIAGFCGPEMLSQYDAHVAAGHI

VRENALPAGIVELLGPILATLDGDVHDSRKEAIMGAFSKEMLASYAPIVFEIVQKEHAAWAAHGGEISLALSCKKTVFKV

FLAILYGITNLTPAEYDAKFDPFRDLLDSFIRAIPKSSKGADAEGLVCKQRLLDELVAPALAASQARVEAKAPVPCFLDY

MLGQTELTPDVVHLEAFHALFAGLGGTQCLVVNTITALAQYPTVAEKVHASRAKFVIKYHDDRWRHFDNLGYCNRFLLEV

KRFYSAGPAQLFGRTTQELTFTTPDGEFAIPKGVLAVAGLDATNRHPDVWTDPSVFNPDRFDNGFSEASDLYKLCPHAIG

KTTGGRKCAGRDLATLVLQASLVSLFDFKWTLVPNQDLSLEEGKSTPMPKGLLMASSFTHRHSESETECDVADWHLLNLP

EAKALVGIAGTVSDDEDDARLDLWTRLMIKLIAKKQARWNKPVANEVLTVPQFQKELPKMTLIQTNIQVATEDEDWPNQP

WLEIQQSNFLRDYAPFVDNFEHTWLPGEDMERYVMSKVGSMWPRVNVHWNDRYSDRALELLAFNGFGQHLLTKLPEAHDD

GSYYGICLNFLKGLEVRPGYAKYGADAFFSAEGKVTKIVRGDVTVRPGDDNWAYAKLCFRGSLQTKITAVDHLLGVHATV

ANIMVIANREQLPPTHPLRRLIKPFTFRSIAINYGAGRALFWPKGMLQRAYALTDKGMKQTWDIGLANFKYETFPEHIAR

QNIDTTTLPFHEDGMDYWHICRSFVSNYVDLYYKSEDALQNDTDVHAFWTFLSTKLPVPMRTLTLENLKDFVAHFIFLVS

SMHNHLGTIAEYVSDPAFCPSAWVEGELAGRPSTGVRLALIMTATGFAQPAITEDFSHIMLDDAAKAVCQAFTAAVTAQI

AVVDARNATRVQPFQSFNPKTMEMAVSI

>CYP5619D1(SDRG_03324.1)Saprolegnia diclina VS20

MVSLPLLVIVIVGQVAGAPQGLGSVLQGVINDVKHSVAGVRYVFESLVDAEPVTGFCSPEALRAFDDALASGALERRTAY

PTGILELTGPTLSTIDGPAFLKRQDAFLNALSGAALSTYQPRIQRRIQEDHATWAARGSTFSLALYAKTSTFKVFLDVVY

GIDDPEKYTGHRAQLDEYLFYLSKTSSRAPSDAAKIREHLLAAIVRPAIASSLARVRSGAPLTCVLDTVVAQGTVSEADL

ALESFQLLAMGLPGLEGLVVHTITAMVSLDDVRGQMATARDAYTAKYPGGAFWSHLDDLDAVNQYVNEVQRVCGASPRHT

FARATKDFSVPSGSGATVAVPKNRLTVVLLDCINNDPKRWPSPEQFQPARFAAANTSAYGFAPFAIDDLVHRAEGRREGL

SRLILQSHVVSLLDFVAVMAPLQSFALGDGVNPLPIDLLTTVSFRYVPGVVQGDIDAWRRLHHPSAKLYNGSLENPLLAA

SDKRLDFWTHSMIQLFNVRFETWVTPTAAASIKVPTTQKNLPKRTLYGTSIQIPTEDEDVAIPKVILESAKLLQDTAPFV

DNFDAKWAPGEDMEGCVLSKVGRMWPRVRVHWDDRYSDRALELLVFNGLGQHMVQKLATAHDDGSYYTVATNYLASIEVR

TGYAITGADAFFDKNGKVTKIVRLGKTIRPIDASWEYVKMCFRSSLVSKITAVDHLIGLHVTVGNYMTTGSREQLPPTHP

LRRLIKPFTFRAVAINYDASIALFAPKGMLHRAFPYTEKGLKDTWAMALKSLTLEPFPVHLARQQVDTITLPYHEDGADY

WEIVRTFVSEYLDLYYTSNDDVTHDVSIQALWTFLNKQLPTPLGVLSLDNLKDVVAHSIFLVTAMHNHLGGIAEYVSDPA

FCPVSWVEGELSGRPGNAVRAALIMSGTGFPQPNILEDFSHVLLDDAAKAVAHRFTASLQAFVHVVEARNAQRIHPYQAF

NPAVMDMAIGI

>CYP5619D2(SDRG_14279.1)Saprolegnia diclina VS20

MVALVPLLLTAVGVVGTQENTLKGFFQGVISDIKHAVTDVRYTFESLVNAEPVTGFCSPDALRAFDDALSSGALSRHAAY

PAGLLDLSGPTLSTLDGQAFAIRQESLLNALSGPSLAAYQPRIQQLIQDDHATWAARGGTFSLALQAKTTTFKVFLAVVY

GVTQPDEYVGYRAQLDEYLEYAKKTLSRAPSDAIKIRDRLLATLVRPAIAASHARVRAGAAPTCVLDALVAQNTMSDSDL

ATEGFQLMAMGLLGLEGLVVHTITAMVSVDGVRGQLGSARDAYVSKYPNGAHWRHLDDLSVVNAYVNEVQRVYNASPRHT

FARATKDFVVTNSSSVPKHSLTAALLDCLNYNAARWPSPAQFQVARFAGANPSAYEFAPFALNDLVDRRAGRREGLSRLI

LQTHVVSLLDFAAVMAPLQSYALDDGLNPLPVDLLTTVGFHYAPGVAHSSNAYDDAWRRLRQPSAKLYNSSIESPLSSDK

RLDFLTHSMIQLLNVRFATWVTPTAAASITVPKSQKPLAKQTLHGTSIQIPVDDEDVSIPKVLLDGAKLLQDTAPFVDNF

DDSWVPGEDMEGYVLSKVGRMWPRVRVHWDDRYSDRALELFVFNGLGQHMVTKLSAAHSDGSYYTATTSFLETLDVRPGY

AVTGADAYFDKNGKVTKIVRLGKTFRPADAQWEYVKMCFRSSVANKVTAVDHLIGLHVTVGNYMTTASREQLPPTHPLRR

LIKPFTFRAVAINYEASKLLFAPKGILHRAHPYSEKGLKDTWAMALQSLKLEPFPVHMARQNIDTLKLPFHEDGMDFWTI

VRGFTGEYLNLYYESDEDVTRDASTQAFWAFLDKQLPTPLGALSLESLKDVVAHGIFLVTAMHNHLGGIAEYVSDPAFCP

VSWVEGELAGRPGAAVRTALIMSGTGYPQPSILEDFSHVLLDDAAKAVAHRFTTSLQSFVMVVEARNAQRVLPYQGFNPA

VMDMAIGI

>CYP5853A1v1(XP_005786468.1)Emiliania huxleyi CCMP1516

MELGLEQSCLVSKNKLRWQTAIARLIGQRDICSTRICSTSAAHAVFARLDLALRSSLVDTAGADLEAGLPTAARLALLAAFEVPVAVATGMDPAAHLDVYVRASGRQPFLFGKGIAVPGYDDVSTLVSSPQQERRAMVLAHPVLIADPVPPACMGGGTLIYLSTGAKHTALRRAIGRAVTGFALKRGRGPPLLFPRGAAPAEWDSRAVRETAPLLGAALDSQANATLETRLALKAAVLASPLGGRLRKANRADKLDADELAQQVADGLLFAGGYGTTHLTLAALERISSDPALYADPDAFLVESARLDPPVTSVSAIAPKGGQQLQGPGGGQLRVAQGVPMQLLLSHANRDPAVFERPYAFDPSRRNLDKVLSWNGVDAAGSCDYSASDRPSEDLELRNEHDDEAMYAYYGAGLVNIVLGVLALIVLGWFCLSQRLAFGALYVKGDAGIIRVQSWLGLLHYLGQAIAAFSWLRLHAVVTHADDARARAESASALRLCFGVMAIFVGAFAVFGTAAACVLFVPAAAKSWIAIAMFWWRHGGVLVLAACAFIAFWVGQVIAEQRGMSLIDYEAGSGIGVTLLARGLLGAEAAPHAWKYEVFARGFLAPSVYFAAAAHIDRVQGRGLGRAYPSELFMRRALRFKHSAPVLLALALSAIHSFAIPRSFGDITGCGEGGGEAAGPECAVDPTGGLDQYTKVYFSIIHLLDDGSQPGPSFVQAPNRTVQPLPKEQVVPGLVLPSYDEDEGLVTSRALANQAFSGYVKDGRLYPLEDLDLPWPEKDGAIEALMGRLSGSLAPAELYDYDANIGGDSLIGDAGNPDFPPPASRRWFRTQFPRDADFTYLAPLTVRPGFERYGAKATFDAAARPVSIWWSHGEKEVRPDDAAWTHAKFAFRSSLLTGVTLKDHLAATHLTIAATLVSASRDHLPATHPLRRLLKPFTYRTIALSLLHHTVALDAAGLEAGFAFAFNRTRDTFDPANPARFLEYPLEYPLSSAAECAATPPSSQAACADEADELAAFAADGTRYRAAVREYVGRYVGIYYADDAAVGSDVVLASFWAALVAHFPRIPPLSSREALVEVLTGFVFHVTAGHRHVGAAYSAVKDPRYAGAKIRPGRDMSDVQAAVQVLAIALVTGFKQPMLLGDYSHVFLRDGHRNATRALWSDFQAKLLQVSAEVDERNRGPRRFKVRAFDPREMATSVSI

>CYP5853A1v2(XP_005778763.1)Emiliania huxleyi CCMP1516

MRATRHLLDAHLLDGTLAVSRARQRRRSHMPSCCFSALVISGTSAILLAGALSSLDGALKFARLDLALRSSLVDTAGADLEAGLPTAARLALLAAFEVPVAVATGMDPAAHLDVYVRASGRQPFLFGKGIAVPGYDDVSTLVSSPQQERRAMVLAHPVLIADPVPPACMGGGTLIYLSTGAKHTALRRAIGRAVTGFALKRGRGPPLLFPRGAAPAEWDSRAVRETAPLLGAALDSQANATLETRLALKAAVLASPLGGRLRKANRADKLDADELAQQVADGLLFAGGYGTTHLTLAALERISSDPALYADPDAFLVESARLDPPVTSVSAIAPKGGQQLQGPGGGQLRVAQGVPMQLLLSHANRDPAVFERPYAFDPSRRNLDKVLSWNGVDAAGSCDYSASDRPSEDLELRNEHDDEAMYAYYGAGLVNIVLGVLALIVLGWFCLSQRLAFGALYVKGDAGIIRVQSWLGLLHYLGQAIAAFSWLRLHAVVTHADDARARAESASALRLCFGVMAIFVGAFAVFGTAAACVLFVPAAAKSWIAIAMFWWRHGGVLVLAACAFIAFWVGQVIAEQRGMSLIDYEAGSGIGVTLLARGLLGAEAAPHAWKYEVFARGFLAPSVYFAAAAHIDRVQGRGLGRAYPSELFMRRALRFKHSAPVLLALALAAIHSFAIPRSFGDITGCGEGGGEAAGPECAVDPTGGLDQYTKVYFSIIHLLDDGSQPGPSFVQAPNRTVQPLPKEQVVPGLVLPSYDEDEGLVTSRALANQAFSGYVKDGRLYPLEDLDLPWPEKDGAIEALMGRLSGSLAPAELYDYDANIGGDSLIGDAGNPDFPPPASRRWFRTHSLEMCARHADFTYLAPLTVRPGFERYGAKATFDAAARPVSIWWSHGEKEVRPDDAAWTHAKFAFRSSLLTGVTLKDHLAATHLTIAATLVSASRDHLPATHPLRRLLKPFTYRTIALSLLHHTVALDAAGLEAGFAFAFNRTRDTFDPANPARFLEYPLEYPLSSAAECAATPPSSQAACADEADELAAFAADGTRYRAAVREYVGRYVGIYYADDAAVGSDVVLASFWAALVAHFPRIPPLSSREALVEVLTGFVFHVTAGHRHVGAAYSAVKDPRYAGAKIRPGRDMSDVQAAVQVLAIALVTGFKQPMLLGDYSHVFLRDGHRNATRALWSDFQAKLLQVSAEVDERNRGPRRFKVRAFDPREMATSVSI

**Table S3. P450 motif sequences used for phylogenetic analysis.**

>CYP5619A1(XP_012203945.1)Saprolegnia_parasitica_CBS223.65

MGNLSSTGAMHGDVHMDKMVMYLDSDRSAMMDGDLFVLEKALATEKVVGFCGPEALKVFDANLRNGTFVRHGALPSGLNELLGPVLPTTDGDAHARKKKLVLAAFSGAQLAAYSPLIRTTIQKEHAKWAAHGASMSLVANAKVLTYKLSLLLILGLEDNYDNSRELLDTYMLALRNSVRRADPTGVRSRDELIRTMINPALATSHDRVHAGTPKPCALDYLVAAGCLSDDDLRTELFHLLCMSLGGLECWVANCITAAASSADVLAQLTAGRDAFMTKYPAEADRWSHLSDLGYVNSYIQEVKRTYVAGPSHMYARATKETDVHTSEGTFRVPKGCLVAAALDGTNKHPSVWANPTKFDPSRFSSTKVDMAFGFCPHAIGAVADRRCAGEELSTLILQSFMVSLFDFMWKMLPHQDYTLDTTLVNPMPKGGLMVVGFHRRTDLSASMVEVAGSEEDWKFLSLPDAK

>CYP5619B2(OQR84828.1a)Achlya_hypogyna

MGSAASSSSTGEVPCLRRAVSLKKMIMFMKDPRTAMMDCRDHYGDFFLVESWLTDEKVMGFCGPEALRAFDAKVAEGLIVRGGSFAPGVLELLGDILPTIDGEAHATRRASIDTAFASEKIGMYKPKIREIVQREHASWAAHGGSISLAQNSRRMVFDVFLAVLFGIEGSFDEHRDLLDTFVSAIRKSARKADAAGLEARRRIVEDLVRPAIRDARGRASVGEAKPVAIDALIADGKLGDNELELELFHALFAGFSGVACLVVNSITAAIEWPEARARVFEARDAFFAKYPTEDDRWSAVELGYIDMYLLEVKRFYVAGPTQIYGRAKEEVALTTAEGTFTIPKGCLATAGLETTNKHPGVWADPHVFNPDRFAADKTAVAKDGTIDVTADEMAYKFCPHSIGSARRCLGEGLTSLVLQCAFVSLLDFVWQMVPDQSYALDEKSATPTPTGQLMAVGFRRRSPGTEHSVAGSEA

>CYP5619A2(OQR84828.1b)Achlya_hypogyna

MRPLTLENLKDFVAHGIFLVSSMHNHLGTIAEYVSDPAFCPSAWVEGELAGRPGTAVRLALIMTATGFTQPAITEDFSHIMLDDAAKAVCHSFTKAVTDQIAVVDARNASRVQPFQSFNPKTMEMAVTSYGDAHMDRMVMYLDADRGAMMDGDLFVLEKALATEKVVGFCGPEALKEFDAKVRDGSFVRQGALPPGLLELLGPVLPTLDGTAHARKKAAVVAALSSSQLNKYKPLIRSVVQDEHARWAAHGASMSLVAYTKQLVFKLALLVLLGLEDNYDHQREQLDTYMTALRNSTRRADPAGVTARAQLIAGLLNPALATAHDRVAAHAPKACVLDFLVGQGQLSDDDLRVELFHMLCMSLGGLECWATNCITAAASNPAVLKQLTAARDAFMTKHPTEDARWAHFQDLGYVNRYISEVKRVYVAGPSHLYARAAKTTDVHTSEGAFTVHAGVLVAAALDGTDKHPSVWPDPTKFNPDRFGAKVDMSYAFCPHAVGAVANRRCPGEELSTLVLQSFLVSLFDFMWKMVPTQDYTLDTTLVNPMPKGGLMVVGFHRRT

>CYP5619A3(OQS07110.1)Thraustotheca_clavata

MGNLTSTRPLGHDCANHKMLLDLGPEALKEFDAKLQNGSFVRQGAFPQGLLDLMGPILPTLDGAAHHAKKAAILEALNGVQVEKYKPVIRSMVQKAHARWSAQGGAMSLVANCKQLAFKLMLVVLLGLENDYDDHRDDHRELLDTYILSLRDSTHRADPDGVRSRQHLLDDMINPALQTSHERLNQNSLKPCVLDFLVSQKKLSDADLRIELFHLLTMGVGGLECWLANCITAAASSPDVLKQLTVARDTYLKKYSNEEDRWRRFDDLGYVNWYIQEVKRVYIAGPSHVYARSTAQVDIVTSDGTFRVPKGALVAAALDTTNKHPKVWTNPGEFNPNRFNKWDETKGMYTFCPHSVGSDRRCPGEQLSTVVLQSFMVSLFDFMWKMIPKQDYSLDTKAVNPMPRGGLMVVGFHRRT

>CYP5619B1(XP_012203946.1)Saprolegnia_parasitica_CBS223.65

MGSQTSTPAGAAPSLRRAASLKKMIMFMKDPRTAMMDCRDHYGDVFLMESSLVNEKIMGFCGPEALLAYDTQVQAGKIVRAGAFPTGVLELLGSVIPTLDGDAHAKRKAALHVAFTPETLDTYKVKIREIIQHEHAAWAARGGSLSLALSCKKLVFHVFSATLLGLENVDDEYRELIETFVSSIRKSARKPDATGMDARTQVVEELIRPAIREAKARVAAEKPLPTVVDVLVADGRLSDEELGLELFHALFAGLGGVTCLAINSITVCIELPAIREKVSAAREAYLTKYPNEDDRWRHFADLGYMQHFLLEVKRFYVAGPTQLYGRATDDLEISTADGSFKVPKGCLATAGLEATSKHPDVWSDPHTFNPDRFAPQDASAQPSGSVDPDELKDGARDVTAPGLMYKFCPHSIGTARRCTGEGLTTLVLQCFVVSLFDFIWQMVPGQNYQLEEKSSTPTPVGQLMAVGFHRRALDRVVTFGTA

>CYP5619B3(OQR84819.1)Achlya_hypogyna

MGNDASVHAEGHAQALPSSNRATSLLKMIAFSKDPRAGMLDARDHYGDLFLLESKVVSEKIAGFCGPELLEAFDSKLAAGEIVREGAFPAGILALLGPILSSLDGAAHTSRKAAVLEALSQAKLETYKPSIRAIVQTEHAAWAARGGAISLALLTRNLVFRIFLQVLYGVEMLDDRHRVALDEFIASIRRSSKAPDPHGVSCRTRILEELIRPAIVKARARIAADAPAPCVLDNLITAAKLDADALEVEAFHFLFAGFGGVACLATNVLTACATHPGVLPKLLEARAEFVTRYPTEDARFAHLDDLGYVNDFLLEVKRYYVAGPTTVFGRAAVDLEVKTSNGVYHLPKGCLAAAGLEATNKHPDVWANPHDFNPDRFKDLDMASHAHRFCPHAFGEASHRRCAGETLTTVILQTIVVSLFDFVWQMVPGQNYALQEGVATPTPVDQLMAVGFHRRTDDAVEFGVA

>CYP5619C2(OQR84821.1)Achlya_hypogyna

MGNSHSVQTSDAPPLPASKRSNSIFSLLNFAKNPNAAMAQGRDTLGDLFLLESAVLSEKIIGFCGPDMLAQYDGQVEAGGIVRAGALPSGIVELLGPILPVLDGNVHAIRKKFVMAAFTEDQLTAYAPTIFSIVQNEHAAWAAHGGSISLGLLSKKLVFKVFLAVLFGLTNIPPIEYDTKYDQYRDEVDGFIAGISKSATAPDAHAVACKQRLITELIGPAIVASQARVKAGAPRPCVLDALVAGDGLSDAQLRLEGLHMLFAGLGGVQCLVVNSLTVMAKFPDISEKLQEARAAFVTRCPTPADRWRHFDQLGYANQFLLEVKRFYTAGPTQLFGRTATELTFQTPDGTYSVPKGALAVAGLNATNKHPEVWADPSVFNPDRFANFDTTTDLYTLCPHSIGKMVGGRRCAGQDLATAVMQASLVSLFDFKWTFAPGQDFTLETGKSTPMPVGNIMVTAFQHRHEVGEDGCDVA

>CYP5619C1(XP_012203939.1)Saprolegnia_parasiticaCBS223.65

MGNQPSTEAGAAPLPDSKRANSIFSLLAFAKDPKAAMAESRDTLGNLFLIESAVVSEKIAGFCGPEMLSQYDAHVAAGHIVRENALPAGIVELLGPILATLDGEVHDSRKEAIMGAFSKDMLASYAPIVFGIVQKEHAAWAAHGGKISLALSCKKTVFKVFLAILYGITDMTPAEYDATYDPFRDLLDGFIRAIPKSSRGADAEGLVCKQRLLDELVAPALAASQARVAAKTPVPCFLDYMLGQTELTPDVVHLEAFHALFAGLGGTQCLVVNTITALAQYPTVAEKVHASRAKFVTKYHEDRWRHFDNLGYCNRFLLEVKRFYNAGPAQLFGRTTQELTFTTPDGEFAIPKGVLAVAGLDATNRHPDVWTDPSVFNPDRFDNGFNEATDLYKLCPHAIGKTTGGRKCAGRDLATLVLQASLVSLFDFKWTLVPNQDLSLEEGKSTPMPKGLLMASAFTHRH

>CYP5619C3(OQR84833.1a)Achlya_Hypogyna

MGNQPSTETGAPPLPDTKRANSIFSMLAFAKNPREAMAESRDTLGNLFLIESAIVSEKIVGFCGPEMLAQYDAQVEAGGIVRDGAFPAGIAELLGPILPALDGEIHAARKAAVMTAFSKEQLALYVPLIFGITQKEHAAWAAHGGAISLALLSKKLVFKVFLAVLYGIETDTPAEYEAKYDHFRDVVDGYIHAIPKSAKAPDADGLRYKARAIDELIAPALAASQARIEAGTPRPCVLDYWVQHSGMQPDDICLEAFHALFAGLGGVQCLVVNTITAMATNPGAAEKLHHTRAEYVLKYHSAEDRSSHFDQLGYANQFLLEVKRFYMAGPSQLFGRTTAELTFQTPDGTYSVPKGALAVAGLNATNKHPEVWADPRVFNPDRFADFDADADLYKLCPHAIGKTNGGRRCAGQDLATAVMQASLVSLFDFKWTFVPGQDFTLETGKSTPMPVGNIMVTAFQHRHEVGEDGCDVA

>CYP5619B4(XP_012203942.1)Saprolegnia_parasitica_CBS223.65

MGNEASTVHADGAATDLPASQRAMNILKMIEFSKDPRAGMLESRDQYGDLFLLESHLVSEKIAGFCGPELLAAFDDKLRDGSIVREGAFPPGIVALLGAIMPTIDGEEHHARKAAALEAFTPARLDLYAPLVREIVQAEHASWAARGGAISLACLTREMVFRIFLKVLYGVERHDANKFRKRPDPHGVSCRTQILDELIRPAIADARARAATKTPAPSVIDCLVTNGKMASDVLETEAFHFLFAGFGGVACLATNILTAVATHPSARKDLLDARAEYVTKYDGEARWAHFHDLGYVNLFILEVKRFYVAGPTAVFGRAKTDLEIPTKNGVYKLPKGCLAAAGLEATNRHPDVWTDPNLFNPNRFRDLGHVRTTKPHAFCPHAFGALSHRRCAGEDLTTLILQSTIVSLFDFVWQMVPNQDYKLAVGVSTPTPVGQLMAVGFHRRTDD

>CYP5619F1(XP_008879406.1)Aphanomyces_invadans

MGASASSFVAKESTSLSTLIAFSKDPRTALLSARDHFGDIFLVESAFVTTRIAGLCGPEALQQFEEKMLEGALVREGAFPPSILALLGPILVTMDGEVHRTKKNALLRAVSPVQLDVYKPIIRRIIQAEHSKWAAHGGAISFALNTKILVFKILLAVLYGMEGEFDSFRTYIEDYVAAIKQSAKTTSAHGVTCRATFIAEILEPAIAAAKARQQVNATSSGPLESVLDVLVASGELNDDDLKNEGFHIMFAGFGGLSAAATNLITAAVVFPEIRAQVFAARDKYLSKFGDDRWGHLDDLGYLNKYILEVKRFFLAGPTQVYAKAARDVDLVTSKGVFHLSKGSLVMAGLEATNHDPDVWAAPNTFDPSRFNDADIDAAHGVKNTRKYSFCPHGFGDVRNRRCAGEELSTIVMQSLLVSYFDFTWKMVPGQNYTLQPHSVTAVPIGLLMAMGFQRQQDDG

>CYP5619G1(XP_008878127.1)Aphanomyces_invadans

MGNVTGHVQREREYVKTVIGFMKDPRTFMSASRNTYGDVFLFQSSLVNQKIAGLSGPEALQAFEARLADGSLVKTGALPSGVSDLLGPIMSVLDGEDHHRKKAGIMTAFTPQQLAKYLLVVRRIIQTEHARWAARGGVISITASSKELVFKLLLAVLYGIEGDFDEYRPLVDEFVASIRKSAVKASPEGKAARDTIMNDLVIPAIEAAKVRVAGGTPSPSALDHLVGLNQLADDDLGVEMFHVLFAGFGGLSCLATNLVTPLVTMPDVREKILDARDQFLSKYTGDTKWDHLEDLGYINQYILEVKRFFVAGPTQSFAKAAVAFDVVTSKGTFHIPKGCLVAAGLETTAFDAEVWPNPDNFDPSRFDNNDDLSALQFKLCPHGIGSTSNRRCAGETLTTLVCQALVVSLFDFTWNMVPGQDYELDENTSIPTPRGGLKAVGFRRRDAVTSYGVAGTD

>CYP5619F2(XP_009834503.1)Aphanomyces_Astaci

MGAAASNYVYNEATSLSTLIGFSKDPRTALLNARDHYGDIFLVESAFVSTKIAGLCGPEALKEFEAKLQDGSLVKQGAFPPSILALLGPILVTLDGDVHHAKKAALLKALSPAQLDVYKPIIRRIVQTEHSKWAAHGGAISFAVNTKILVFKVLLAVLYGVEGEFDTYRRYVDDYVTAIKQSAKVTDEHGVTCRAKFIAEIIAPAIAAAKANQTKRQQQPLNSVLDVLVATGDLTDDDDLQNEMFHFMFAGFGGVSAAATNLITAVCVFPDIRAKVLRARDDFLRQYDGRDESPWNHLDEMGYLNLFVLEVKRYFVAGPTQVYAKAARDLDLVTSTGVFRIPEGALVMAGLEATNRDPDTWPSPDSFDPTRFTQADVDGMHMTRPFSFCPHGFGSHRRCAGEQLTTVIMQSVLVSLFDFTWKMIPGQEYALQPHSVTAVPIGQLMGVNFHRRLNEDD

>CYP5619D1(XP_012194083.1)Saprolegnia_parasitica_CBS223.65

MVSIPLLLITIVGQAAGAPQGLGSVLQGVINDVKHSVAGVRYAFESLVDAEPVTGFCSPEALRAFDDALISGALERRTAYPAGILELSGPTLSTIDGPAFSKRQDAFLNALSGPALAAYQPRIQRRIQEDHAMWAARGSTFSLALHAKTSVFKVFLDVVYGVSDPEKYTGYRAQLDEYLFYVSKTSRRAPADATKIRERLLAAIVRPAIASSVARVRSGASTTCVLDAVVAQGSVSEADLVVESFQLLAMGLPGLEGLVVHTITAMVSHDDVRGQMATARDAYTAQYPNGAHWSHLEDLDAVNQYVNEVQRVYGASPRHTFARATKDLTVPDGSGAMVAVPKNRLTVALLDCINHDPKRWPSPEQFQPARFATANTSAYGFAPFAIDDLVHRVEGRREGLSRLILQSHVVSLLDFVAVMAPLQSFALGDGVNPLP

>CYP5619D3(AIG56338.1)Achlya_hypogyna

MVAVSWLWCFGPTLVAAEPQGLGSFFQGIISDIKHAVNDLTFRFEHLVDAEPVTGFCGPDALRAFDNYLATGALVRHDAYPKGVLDLVGSTLATLDGSAFATRQAAFLNALSPAAVQRYKSTVHNIVQADHATWAARGGTFSLANAAKVTTFKVVLAVVLGLDNPEAYTGYRSQIDEYLALLAQTEWRAPADAVTIRSRLLAALIRPAVVAAHARATPKSCVVDALVEAGTVSDEDLATELFQLLVHGIPGLEGLVVHSLTAIASVDGVRAHLASARDVYMAKYYGAARWDHFDDLGYGNQFLLEVQRTYTASPRQEYARATVDLKVLTPTGTTIVPKNRLKVGVLECLNKDAKRWPNPTSFDPTRFASANTSAYAFAPYAMNLLADRRRGVGEALSQLVLQTHLVSLWDFAWTMAPRQSYALADSPNPSPVD

>CYP5619D4(AIG56100.1)Achlya_hypogyna

MVSITRLLHLSLAAATVAGAPQGFFQDLISDIRHGIAETLFGLEQLVAAEPVVGFCSPEAIRAFDELIAAGALQRQSAYPKGVQNLVGSTTLTLDGPAFAARQAALLAALSPMAVQTYAPTIRAIVQADHATWAARGGLFSLDDAARTMTFKVFVAVVLGLESPERYTGYRAQLDDYLSYLRVTASVAPPEAVAIRKRLLDTLVRPAITAARARSTPKPSVVDILVAMGSVADADLADEIFALLANGLPGLEGLVVHTLSTMASVEGVVANLATARDVYLAKYPGAARWQHLDELGYANQFLLEVQRTYGAKPSHVFARATKELSVAGVKVPKNRLTAVLLECLNQDPGRWPEPARFDPSRFAVANTSAYEFAPFAMNLLTDRPHGIREALTTTVLQTHVVSLFDFVWSMAPHQNYTVEAGVNAGPVDGLMTVSF

>CYP5619D5(AIG56283.1)Achlya_hypogyna

MVSITRLLRLSLAAATVAGAPQGFFQDLISDIRHGIAETLFGLEQLVAAEPVVGFCSPEAIRAFDELIAAGALQRQSAYRKGVQNLVGSTTLTLDGPAFAARQAALLAALSPAAVQTYAPTIRAIVQADHATWAARGGLFSLADAARTMTFKVFVAVVLGLESPERYTGYRAQLDDYLSYLRVTASVAPPEAVAIRKRLLDTLVRPAITAARARSTPKPSVVDSLVAMGSVAEADLADEIFALLANGLPGLEGLVVHTLTTMASVEGVVANLATARDVYLAKYPGAARWQHLDELGYANQFLLEVQRTYGAKPSHAFARATKELSVAGAKVPKNRLTAVLLECLNQDPGRWPEPGRFDPSRFAIANTSAYAFAPFAMNLLTDRPHGMREALTTTVLQTHVVSLFDFVWSMAPHQNYTVEAGVNAGPVDGLMTVGF

>CYP5619A1(SDRG_14280.1)Saprolegnia diclina VS20

HMDKMVMYLDGDRSAMMDGDLFVLEKALATEKVVGFCGPEALKVFDANLRDGTFVRHGALPSGLNELLGAVLPTTDGDAHARKKKLVLAAFSGAQLAAYKPLIRTTIQNEHAKWAAHGASMSLVANAKVLVFKLSLLLILGLEDNYDNSRELLDTYMLALRNSVRRADPAGVRSRDELIRTMINPALATSHDRVHTGKPKPCALDHLVAAGVLSDDDLRAELFHLLCMSLGGLECWVANCITAAASSTDVLAQLTAGRDAFITKYPAEADRWSHLGDLGYVNNYIQEVKRTYVAGPSHMYARATKDTDVRTSEGTFHVPKGCLVAAALDGTNKHPSVWANPTKFDPSRFSTAKVDMAFGFCPHAIGADRRCAGEELSTLILQSFMVSLFDFMWKMLPHQDYTLDTTLVNPMPKGGLMV

>CYP5619B1(SDRG_14281.1)Saprolegnia diclina VS20

PRTAMMDCRDHYGDVFLMESSLVNEKIMGFCGPEALLAYDTQVKEGKIVRASAFPTGILELLGAVVSTLDDDAHAKRKAALLVAFTPEKLDAYKPKIREIIQHDHAAWAARGGSLSLALSCKKMVFHVFMATLLGLENVDDEYRELVEAFVSSIRKSARKPDTTGMDARTQVVEELIRPAVREAKARVAAQKPLPTVVEVLVADGRLSDEELNLELFHALFAGLGGVTCLVINAVTACIELPAIREKVSAAREAFLAKYPNEDDRWSHFADLGYMHHFILEVKRFYVAGPTQLYGRATDDLEISTANGSFKVPKGCLATAGLEVTSKHPDVWSDPHTFNPDRFAPQDASTTPVDPDAFKDGARDVTAPDMMYKFCPHSIGIARRCAGEGLTTLVLQCFVVSLFDFIWQMVPGQNYQLEEKSST

>CYP5619B2(SDRG_14277.1)Saprolegnia diclina VS20

PRAGMLESRDQFGDLFLLESHLVSEKIAGFCGPELLAAFDDKLRDGSIVREGAFPPGVLALLGPIMSTIDGEEHDARKAAALEALTPARLDLYAPIIREIVEAEHASWAARGGAISLACLTRDMVFRIFLKVLYGVERHDGNKFRVLLDDFIVSIRRSSKHADPHGVRCRTQILDELIRPAIANAQARASNKTPVPSVIDCLVANGKMTPDVLETEAFHFLFAGFGGVACLATNILTAVATHPSARKDLLDARAEYVTKYDGDARWAHFHDLGYVNLFILEVKRFYVAGPTAVFGRTKTDLEIPTKNGVYKLPKGCLAAAGLEATNRHPDVWTDPNLFNPNRFRDLGHVRTTKPHAFCPHAFGESSHRRCAGEDLTTLILQSTVVSLYDFVWQMVPNQDYKLAVGSSTPTPVGQLMAVGFHR

>CYP5619C1(SDRG_14273.1)Saprolegnia diclina VS20

AGIVELLGPILATLDGDVHDSRKEAIMGAFSKEMLASYAPIVFEIVQKEHAAWAAHGGEISLALSCKKTVFKVFLAILYGITNLTPAEYDAKFDPFRDLLDSFIRAIPKSSKGADAEGLVCKQRLLDELVAPALAASQARVEAKAPVPCFLDYMLGQTELTPDVVHLEAFHALFAGLGGTQCLVVNTITALAQYPTVAEKVHASRAKFVIKYHDDRWRHFDNLGYCNRFLLEVKRFYSAGPAQLFGRTTQELTFTTPDGEFAIPKGVLAVAGLDATNRHPDVWTDPSVFNPDRFDNGFSEASDLYKLCPHAIGKTTGGRKCAGRDLATLVLQASLVSLFDFKWTLVPN

>CYP5619D1(SDRG_03324.1)Saprolegnia diclina VS20

ESLVDAEPVTGFCSPEALRAFDDALASGALERRTAYPTGILELTGPTLSTIDGPAFLKRQDAFLNALSGAALSTYQPRIQRRIQEDHATWAARGSTFSLALYAKTSTFKVFLDVVYGIDDPEKYTGHRAQLDEYLFYLSKTSSRAPSDAAKIREHLLAAIVRPAIASSLARVRSGAPLTCVLDTVVAQGTVSEADLALESFQLLAMGLPGLEGLVVHTITAMVSLDDVRGQMATARDAYTAKYPGGAFWSHLDDLDAVNQYVNEVQRVCGASPRHTFARATKDFSVPSGSGATVAVPKNRLTVVLLDCINNDPKRWPSPEQFQPARFAAANTSAYGFAPFAIDDLVHRAEGRREGLSRLILQSHVVSLLDFVAVMAPLQSFALGDGVNPLPIDLLTTVSFRYVPGVV

>CYP5619D2(SDRG_14279.1)Saprolegnia diclina VS20

IKHAVTDVRYTFESLVNAEPVTGFCSPDALRAFDDALSSGALSRHAAYPAGLLDLSGPTLSTLDGQAFAIRQESLLNALSGPSLAAYQPRIQQLIQDDHATWAARGGTFSLALQAKTTTFKVFLAVVYGVTQPDEYVGYRAQLDEYLEYAKKTLSRAPSDAIKIRDRLLATLVRPAIAASHARVRAGAAPTCVLDALVAQNTMSDSDLATEGFQLMAMGLLGLEGLVVHTITAMVSVDGVRGQLGSARDAYVSKYPNGAHWRHLDDLSVVNAYVNEVQRVYNASPRHTFARATKDFVVTNSSSVPKHSLTAALLDCLNYNAARWPSPAQFQVARFAGA

>CYP5853A1v1(XP_005786468.1)Emiliania huxleyi CCMP1516

QPFLFGKGIAVPGYDDVSTLVSSPQQERRAMVLAHPVLIADPVPPACMGGGTLIYLSTGAKHTALRRAIGRAVTGFALKRGRGPPLLFPRGAAPAEWDSRAVRETAPLLGAALDSQANATLETRLALKAAVLASPLGGRLRKANRADKLDADELAQQVADGLLFAGGYGTTHLTLAALERISSDPALYADPDAFLVESARLDPPVTSVSAIAPKGGQQLQGPGGGQLRVAQGVPMQLLLSHANRDPAVFERPYAFDPSRR

>CYP5853A1v2(XP_005778763.1)Emiliania huxleyi CCMP1516

RQPFLFGKGIAVPGYDDVSTLVSSPQQERRAMVLAHPVLIADPVPPACMGGGTLIYLSTGAKHTALRRAIGRAVTGFALKRGRGPPLLFPRGAAPAEWDSRAVRETAPLLGAALDSQANATLETRLALKAAVLASPLGGRLRKANRADKLDADELAQQVADGLLFAGGYGTTHLTLAALERISSDPALYADPDAFLVESARLDPPVTSVSAIAPKGGQQLQGPGGGQLRVAQGVPMQLLLSHANRDPAVFERPYAFDPSRRN

>CYP5851A1(OQS03666.1)Thraustotheca_clavata

MWNCFSGSGDEAFPSGKVPYITDEQVQHMHGVIYLLEYAVGEENVAVLKGSNLIQQFDTHRQNGNLSRQDALPIGLVDLAGKTLSTLDNATFAKRQSALLDAFSIEQVAKYQSKIDAIVQSRHSAWAARGGSFSIAVETKKLVFHIFVGVILGLEDQYDAVFNLVNQYRELLPKSLRRPHAKAITLRQEILSKLITPAVTSSRTRVANKQTNDSVVDYLIRKGQLSDADITIELFQALIDGTDGISSLVINCVNAWVNQPGLSDKLASVRDAPDTFVNQFIDEVERVYTAGPSHEYARVVKNTTFTTPKGSFTLPKGQLVVAFTESINEDASVWPNPTLFDPSRFENDTPDPYKFTAFSLLQLVNRVQNVREAFTKAVLRSNMSSLLNCMWQMVPLQSYELTEHTVTNPTPVGQLTVVNFHKRH

>CYP5852A1(OQR84833.1b)Achlya_hypogyna

MTSRNKVMALPDNASVSLRDLVADATAMAELRLLLSNQKLMGAYGARLLAVLEEQAYIAQQPAVQRRPPPATEWTGGLHKLQTFAKDPCGTSLALHAKYGDIFFLDSVWSSTMIAGVAGPSLLMAFDDHWNAGRLGSAVPSGVLPLLGPVLPTLDGSKHRARKAALLAGVVPATHAVVIANMVADELEAWAAAECTFSFVVRAQSLALKLLLRILLGITGASPLLLGNCQHWIDTLVAAVPASTVAPVPQGLLAKERLLADLCRPAVAASRTRFASKAAVTCVLDALVERNELPDEVLALELLHCLCTGVAPLGSLLANTVTASHKFPAVWSKLQRSASAYTQSKIDGALWEYGSHFAMEVQRFYSAGSSLRYGRAKTDLIFTAGEVVYTLPKDSLVVAGVRATHVRAASWAVPHHFNPDRFAAGVEKGAWQPLRLGGFCDALSTRIVEAWALALTNYSWHLVPGQDFAVDRAVPSSVPAGKLVASHFRR

>CYP5852B1(XP_012203940.1)Saprolegnia_parasitica_CBS223.65

MGNQPSGRTKVMALPPPDAKLHDLATDPVIMADLRKLLSNRSIAGAYGPLLLAAIEEHVGATPQPVAMVQRRPAPSTEWSGGLSKLRAFAAAPVASFEALHATYGDLFYIESVWTSDKIAGVAGPTLVAAFEDHMDACRLARSVPSGVTHLLGPVLATLNGPSYKARWTNLASAFAPGHQFEPVVQRLFRDELAAAHAAGRTFSFTVLAQHLVLKLLLSLLLGVTASSQLELANVQHWIDTMVAALPRSTVAPHNDALQAKEQLLATLLQPALLASRRRVDAKAPVACVLDNLVLKNDLSDDVILLELLHALYTGAGPLAALLANTISASHAYPAVWAKLVADTRAHKQQSPGAWKFGRAFAKEIQRFYRVGSGLRFARATSDITFAVNDVVYTVPKHTVVVAGIDATHKHAASWSAPADFIPNRFLDDAESTKNALHLFQLGGVSDALPTMVLESWLLAVADYTWFLTPGQETSLDKASVTSPLPVGKLIASHMERR

>CYP5851A2(OQS07119.1)Thraustotheca_clavata

KDCVVDYLVQQAQITDADITIELFQALIDGTDGISSLIINCVTAWVKQPGMSDKLASIRDSPDAFVDQFINEVERVYTAGPNHEYARVLTKTTFTTPKSSFSLTKGQLVVVFTESINEDVTVWSNPTSFNPSRFENGTPEPYKFTSFNLLQLVNRAQGVREEFTKAVLRSNMLSLLTC

>CYP51F1(NP_011871.1)S. cerevisiae S288C

MSATKSIVGEALEYVNIGLSHFLALPLAQRISLIIIIPFIYNIVWQLLYSLRKDRPPLVFYWIPWVGSAVVYGMKPYEFFEECQKKYGDIFSFVLLGRVMTVYLGPKGHEFVFNAKLADVSAEAAYAHLTTPVFGKGVIYDCPNSRLMEQKKFVKGALTKEAFKSYVPLIAEEVYKYFRDSKNFRLNERTTGTIDVMVTQPEMTIFTASRSLLGKEMRAKLDTDFAYLYSDLDKGFTPINFVFPNLPLEHYRKRDHAQKAISGTYMSLIKERRKNNDIQDRDLIDSLMKNSTYKDGVKMTDQEIANLLIGVLMGGQHTSAATSAWILLHLAERPDVQQELYEEQMRVLDGGKKELTYDLLQEMPLLNQTIKETLRMHHPLHSLFRKVMKDMHVPNTSYVIPAGYHVLVSPGYTHLRDEYFPNAHQFNIHRWNKDSASSYSVGEEVDYGFGAISKGVSSPYLPFGGGRHRCIGEHFAYCQLGVLMSIFIRTLKWHYPEGKTVPPPDFTSMVTLPTGPAKIIWEKRNPEQKI

**Figure S1. PROMALS3D analysis of CYP5619 family.**

[**Colored**](http://prodata.swmed.edu/promals3d/info/promals_output.html)**PROMALS3D alignment (sequences in aligned order)**

Conservation: 95

CYP5619D3_AIG56338.1_Achl 1 MV-------------------------------------------------------------------- 2

CYP5619D2_SDRG_14279.1_Sa 1 MV-------------------------------------------------------------------- 2

CYP5619D1_SDRG_03324.1_Sa 1 MV-------------------------------------------------------------------- 2

CYP5619D1_XP_012194083.1_ 1 MV-------------------------------------------------------------------- 2

CYP5619D4_AIG56100.1_Achl 1 MV-------------------------------------------------------------------- 2

CYP5619D5_AIG56283.1_Achl 1 MV-------------------------------------------------------------------- 2

CYP5619A1_XP_012203945.1_ 1 MGNLS----------------------------------------------------------------- 5

CYP5619A1_SDRG_14280.1_Sa 1 MGNLT----------------------------------------------------------------- 5

CYP5619B2_SDRG_14277.1_Sa 1 MGNEA----------------------------------------------------------------- 5

CYP5619B4_XP_012203942.1_ 1 MGNEA----------------------------------------------------------------- 5

CYP5619B3_OQR84819.1_Achl 1 MGNDA----------------------------------------------------------------- 5

CYP5619C3_OQR84833.1a_Ach 1 MGNQP----------------------------------------------------------------- 5

CYP5619C2_OQR84821.1_Achl 1 MGNSH----------------------------------------------------------------- 5

CYP5619C1_XP_012203939.1_ 1 MGNQP----------------------------------------------------------------- 5

CYP5619C1_SDRG_14273.1_Sa 1 MGNQP----------------------------------------------------------------- 5

CYP5619B1_SDRG_14281.1_Sa 1 MGSQA----------------------------------------------------------------- 5

CYP5619B1_XP_012203946.1_ 1 MGSQT----------------------------------------------------------------- 5

CYP5619B2_OQR84828.1a_Ach 1 MGSAA----------------------------------------------------------------- 5

CYP5619F2_XP_009834503.1_ 1 MGAAA----------------------------------------------------------------- 5

CYP5619F1_XP_008879406.1_ 1 MGASA----------------------------------------------------------------- 5

CYP5619G1_XP_008878127.1_ 1 MGNVT----------------------------------------------------------------- 5

CYP5619A3_OQS07110.1_Thra 1 MGNLT----------------------------------------------------------------- 5

CYP5619A2_OQR84828.1b_Ach 1 MRPLTLENLKDFVAHGIFLVSSMHNHLGTIAEYVSDPAFCPSAWVEGELAGRPGTAVRLALIMTATGFTQ 70

[Consensus_aa:](http://prodata.swmed.edu/promals3d/info/consensus.html) **M**s....................................................................

[Consensus_ss:](http://prodata.swmed.edu/promals3d/info/consensus_ss.html) e

Conservation: 5

CYP5619D3_AIG56338.1_Achl 3 -------------------------------------------------------------------AVS 5

CYP5619D2_SDRG_14279.1_Sa 3 -------------------------------------------------------------------ALV 5

CYP5619D1_SDRG_03324.1_Sa 3 -------------------------------------------------------------------SLP 5

CYP5619D1_XP_012194083.1_ 3 -------------------------------------------------------------------SIP 5

CYP5619D4_AIG56100.1_Achl 3 -------------------------------------------------------------------SIT 5

CYP5619D5_AIG56283.1_Achl 3 -------------------------------------------------------------------SIT 5

CYP5619A1_XP_012203945.1_ 6 ---------------------STGAMHGDV---------------------------------HMDKMVM 21

CYP5619A1_SDRG_14280.1_Sa 6 ---------------------STGATHGDV---------------------------------HMDKMVM 21

CYP5619B2_SDRG_14277.1_Sa 6 ---------------------STVHADGAATDLPASHRAM-----------------------NILKMIE 31

CYP5619B4_XP_012203942.1_ 6 ---------------------STVHADGAATDLPASQRAM-----------------------NILKMIE 31

CYP5619B3_OQR84819.1_Achl 6 ---------------------S-VHAEGHAQALPSSNRAT-----------------------SLLKMIA 30

CYP5619C3_OQR84833.1a_Ach 6 ---------------------S-TET--GAPPLPDTKRAN-----------------------SIFSMLA 28

CYP5619C2_OQR84821.1_Achl 6 ---------------------S-VQT-SDAPPLPASKRSN-----------------------SIFSLLN 29

CYP5619C1_XP_012203939.1_ 6 ---------------------S-TEA--GAAPLPDSKRAN-----------------------SIFSLLA 28

CYP5619C1_SDRG_14273.1_Sa 6 ---------------------S-TEA--GVAPLPDSKRAN-----------------------SIFSLLA 28

CYP5619B1_SDRG_14281.1_Sa 6 ---------------------S-TPA-GAA---PSLRRAA-----------------------SLKKMIM 26

CYP5619B1_XP_012203946.1_ 6 ---------------------S-TPA-GAA---PSLRRAA-----------------------SLKKMIM 26

CYP5619B2_OQR84828.1a_Ach 6 ---------------------S-SSSTGEV---PCLRRAV-----------------------SLKKMIM 27

CYP5619F2_XP_009834503.1_ 6 ---------------------S-----NYV-----YNEAT-----------------------SLSTLIG 21

CYP5619F1_XP_008879406.1_ 6 ---------------------S-----SFV-----AKEST-----------------------SLSTLIA 21

CYP5619G1_XP_008878127.1_ 6 ---------------------------GHV-----QRERE-----------------------YVKTVIG 20

CYP5619A3_OQS07110.1_Thra 6 ---------------------STRPLGHDC---------------------------------ANHKMLL 21

CYP5619A2_OQR84828.1b_Ach 71 PAITEDFSHIMLDDAAKAVCHSFTKAVTDQIAVVDARNASRVQPFQSFNPKTMEMAVTSYGDAHMDRMVM 140

[Consensus_aa:](http://prodata.swmed.edu/promals3d/info/consensus.html) ...................................................................*hl*.

[Consensus_ss:](http://prodata.swmed.edu/promals3d/info/consensus_ss.html) e eee hhh

Conservation: 55 7 7 7 657 95679757 77 9

CYP5619D3_AIG56338.1_Achl 6 WL--WCFGPTLVAAEPQGLGSFFQGIISDIKHAVNDLTFRFEHLVDAEPVTGFCGPDALRAFDNYLATGA 73

CYP5619D2_SDRG_14279.1_Sa 6 PL--LLTAVGVVGTQENTLKGFFQGVISDIKHAVTDVRYTFESLVNAEPVTGFCSPDALRAFDDALSSGA 73

CYP5619D1_SDRG_03324.1_Sa 6 LL--VIVIVGQVAGAPQGLGSVLQGVINDVKHSVAGVRYVFESLVDAEPVTGFCSPEALRAFDDALASGA 73

CYP5619D1_XP_012194083.1_ 6 LL--LITIVGQAAGAPQGLGSVLQGVINDVKHSVAGVRYAFESLVDAEPVTGFCSPEALRAFDDALISGA 73

CYP5619D4_AIG56100.1_Achl 6 RLLHLSLAAATVAGAPQG---FFQDLISDIRHGIAETLFGLEQLVAAEPVVGFCSPEAIRAFDELIAAGA 72

CYP5619D5_AIG56283.1_Achl 6 RLLRLSLAAATVAGAPQG---FFQDLISDIRHGIAETLFGLEQLVAAEPVVGFCSPEAIRAFDELIAAGA 72

CYP5619A1_XP_012203945.1_ 22 YL--DSDRSAMM-D-----GD----------------LFVLEKALATEKVVGFCGPEALKVFDANLRNGT 67

CYP5619A1_SDRG_14280.1_Sa 22 YL--DGDRSAMM-D-----GD----------------LFVLEKALATEKVVGFCGPEALKVFDANLRDGT 67

CYP5619B2_SDRG_14277.1_Sa 32 FS--KDPRAGML-ESRDQFGD----------------LFLLESHLVSEKIAGFCGPELLAAFDDKLRDGS 82

CYP5619B4_XP_012203942.1_ 32 FS--KDPRAGML-ESRDQYGD----------------LFLLESHLVSEKIAGFCGPELLAAFDDKLRDGS 82

CYP5619B3_OQR84819.1_Achl 31 FS--KDPRAGML-DARDHYGD----------------LFLLESKVVSEKIAGFCGPELLEAFDSKLAAGE 81

CYP5619C3_OQR84833.1a_Ach 29 FA--KNPREAMA-ESRDTLGN----------------LFLIESAIVSEKIVGFCGPEMLAQYDAQVEAGG 79

CYP5619C2_OQR84821.1_Achl 30 FA--KNPNAAMA-QGRDTLGD----------------LFLLESAVLSEKIIGFCGPDMLAQYDGQVEAGG 80

CYP5619C1_XP_012203939.1_ 29 FA--KDPKAAMA-ESRDTLGN----------------LFLIESAVVSEKIAGFCGPEMLSQYDAHVAAGH 79

CYP5619C1_SDRG_14273.1_Sa 29 FA--KDPKAAMA-ESRDTLGN----------------LFLIESAVVSEKIAGFCGPEMLSQYDAHVAAGH 79

CYP5619B1_SDRG_14281.1_Sa 27 FM--KDPRTAMM-DCRDHYGD----------------VFLMESSLVNEKIMGFCGPEALLAYDTQVKEGK 77

CYP5619B1_XP_012203946.1_ 27 FM--KDPRTAMM-DCRDHYGD----------------VFLMESSLVNEKIMGFCGPEALLAYDTQVQAGK 77

CYP5619B2_OQR84828.1a_Ach 28 FM--KDPRTAMM-DCRDHYGD----------------FFLVESWLTDEKVMGFCGPEALRAFDAKVAEGL 78

CYP5619F2_XP_009834503.1_ 22 FS--KDPRTALL-NARDHYGD----------------IFLVESAFVSTKIAGLCGPEALKEFEAKLQDGS 72

CYP5619F1_XP_008879406.1_ 22 FS--KDPRTALL-SARDHFGD----------------IFLVESAFVTTRIAGLCGPEALQQFEEKMLEGA 72

CYP5619G1_XP_008878127.1_ 21 FM--KDPRTFMS-ASRNTYGD----------------VFLFQSSLVNQKIAGLSGPEALQAFEARLADGS 71

CYP5619A3_OQS07110.1_Thra 22 ----------------------------------------------------DLGPEALKEFDAKLQNGS 39

CYP5619A2_OQR84828.1b_Ach 141 YL--DADRGAMM------DGD----------------LFVLEKALATEKVVGFCGPEALKEFDAKVRDGS 186

[Consensus_aa:](http://prodata.swmed.edu/promals3d/info/consensus.html) *h*...bss.st*hh*..t.p.*h***G**s................*h***F***hh***E**p*hl*ss**E**.*lh***G***h***C**t**PE***h***L**..*@***D**s.*l*.s**G**.

[Consensus_ss:](http://prodata.swmed.edu/promals3d/info/consensus_ss.html) e hhhhhhhhhhhhhhhhhhhhhhh eeeeeee hhhhhhhhhhhhhhh

Conservation: 5 7 756 75 9 9 5 97 7 75 95 5 9 5 7 57 597 979596 96

CYP5619D3_AIG56338.1_Achl 74 LVRHDAYPKGVLDLVGSTLATLDGSAFATRQAAFLNALSPAAVQRYKSTVHNIVQADHATWAARGGTFSL 143

CYP5619D2_SDRG_14279.1_Sa 74 LSRHAAYPAGLLDLSGPTLSTLDGQAFAIRQESLLNALSGPSLAAYQPRIQQLIQDDHATWAARGGTFSL 143

CYP5619D1_SDRG_03324.1_Sa 74 LERRTAYPTGILELTGPTLSTIDGPAFLKRQDAFLNALSGAALSTYQPRIQRRIQEDHATWAARGSTFSL 143

CYP5619D1_XP_012194083.1_ 74 LERRTAYPAGILELSGPTLSTIDGPAFSKRQDAFLNALSGPALAAYQPRIQRRIQEDHAMWAARGSTFSL 143

CYP5619D4_AIG56100.1_Achl 73 LQRQSAYPKGVQNLVGSTTLTLDGPAFAARQAALLAALSPMAVQTYAPTIRAIVQADHATWAARGGLFSL 142

CYP5619D5_AIG56283.1_Achl 73 LQRQSAYRKGVQNLVGSTTLTLDGPAFAARQAALLAALSPAAVQTYAPTIRAIVQADHATWAARGGLFSL 142

CYP5619A1_XP_012203945.1_ 68 FVRHGALPSGLNELLGPVLPTTDGDAHARKKKLVLAAFSGAQLAAYSPLIRTTIQKEHAKWAAHGASMSL 137

CYP5619A1_SDRG_14280.1_Sa 68 FVRHGALPSGLNELLGAVLPTTDGDAHARKKKLVLAAFSGAQLAAYKPLIRTTIQNEHAKWAAHGASMSL 137

CYP5619B2_SDRG_14277.1_Sa 83 IVREGAFPPGVLALLGPIMSTIDGEEHDARKAAALEALTPARLDLYAPIIREIVEAEHASWAARGGAISL 152

CYP5619B4_XP_012203942.1_ 83 IVREGAFPPGIVALLGAIMPTIDGEEHHARKAAALEAFTPARLDLYAPLVREIVQAEHASWAARGGAISL 152

CYP5619B3_OQR84819.1_Achl 82 IVREGAFPAGILALLGPILSSLDGAAHTSRKAAVLEALSQAKLETYKPSIRAIVQTEHAAWAARGGAISL 151

CYP5619C3_OQR84833.1a_Ach 80 IVRDGAFPAGIAELLGPILPALDGEIHAARKAAVMTAFSKEQLALYVPLIFGITQKEHAAWAAHGGAISL 149

CYP5619C2_OQR84821.1_Achl 81 IVRAGALPSGIVELLGPILPVLDGNVHAIRKKFVMAAFTEDQLTAYAPTIFSIVQNEHAAWAAHGGSISL 150

CYP5619C1_XP_012203939.1_ 80 IVRENALPAGIVELLGPILATLDGEVHDSRKEAIMGAFSKDMLASYAPIVFGIVQKEHAAWAAHGGKISL 149

CYP5619C1_SDRG_14273.1_Sa 80 IVRENALPAGIVELLGPILATLDGDVHDSRKEAIMGAFSKEMLASYAPIVFEIVQKEHAAWAAHGGEISL 149

CYP5619B1_SDRG_14281.1_Sa 78 IVRASAFPTGILELLGAVVSTLDDDAHAKRKAALLVAFTPEKLDAYKPKIREIIQHDHAAWAARGGSLSL 147

CYP5619B1_XP_012203946.1_ 78 IVRAGAFPTGVLELLGSVIPTLDGDAHAKRKAALHVAFTPETLDTYKVKIREIIQHEHAAWAARGGSLSL 147

CYP5619B2_OQR84828.1a_Ach 79 IVRGGSFAPGVLELLGDILPTIDGEAHATRRASIDTAFASEKIGMYKPKIREIVQREHASWAAHGGSISL 148

CYP5619F2_XP_009834503.1_ 73 LVKQGAFPPSILALLGPILVTLDGDVHHAKKAALLKALSPAQLDVYKPIIRRIVQTEHSKWAAHGGAISF 142

CYP5619F1_XP_008879406.1_ 73 LVREGAFPPSILALLGPILVTMDGEVHRTKKNALLRAVSPVQLDVYKPIIRRIIQAEHSKWAAHGGAISF 142

CYP5619G1_XP_008878127.1_ 72 LVKTGALPSGVSDLLGPIMSVLDGEDHHRKKAGIMTAFTPQQLAKYLLVVRRIIQTEHARWAARGGVISI 141

CYP5619A3_OQS07110.1_Thra 40 FVRQGAFPQGLLDLMGPILPTLDGAAHHAKKAAILEALNGVQVEKYKPVIRSMVQKAHARWSAQGGAMSL 109

CYP5619A2_OQR84828.1b_Ach 187 FVRQGALPPGLLELLGPVLPTLDGTAHARKKAAVVAALSSSQLNKYKPLIRSVVQDEHARWAAHGASMSL 256

[Consensus_aa:](http://prodata.swmed.edu/promals3d/info/consensus.html) *l*s**R**ps**A***h***P**.**G***l*..**L***h***G**s*hh*s**T***l***DG**.*h@*..**+**p.t*hh*.**A***h*os..*l*..**Y**.**P**.*l*pp*hl***Q**.**-HA**.**WAA+GG**s*h***SL**

[Consensus_ss:](http://prodata.swmed.edu/promals3d/info/consensus_ss.html) h hhhhhhhh ee hhhhhhhhhhhhhh hhhhhhhhhhhhhhhhhhhhhhhh h

Conservation: 7 57 5 5 559 9 57 7 7 55 7 7 55 9

CYP5619D3_AIG56338.1_Achl 144 ANAAKVTTFKVVLAVVLGLDNPE------AYTGYRSQIDEYLALLAQTEWRAPADAVTIRSRLLAALIRP 207

CYP5619D2_SDRG_14279.1_Sa 144 ALQAKTTTFKVFLAVVYGVTQPD------EYVGYRAQLDEYLEYAKKTLSRAPSDAIKIRDRLLATLVRP 207

CYP5619D1_SDRG_03324.1_Sa 144 ALYAKTSTFKVFLDVVYGIDDPE------KYTGHRAQLDEYLFYLSKTSSRAPSDAAKIREHLLAAIVRP 207

CYP5619D1_XP_012194083.1_ 144 ALHAKTSVFKVFLDVVYGVSDPE------KYTGYRAQLDEYLFYVSKTSRRAPADATKIRERLLAAIVRP 207

CYP5619D4_AIG56100.1_Achl 143 DDAARTMTFKVFVAVVLGLESPE------RYTGYRAQLDDYLSYLRVTASVAPPEAVAIRKRLLDTLVRP 206

CYP5619D5_AIG56283.1_Achl 143 ADAARTMTFKVFVAVVLGLESPE------RYTGYRAQLDDYLSYLRVTASVAPPEAVAIRKRLLDTLVRP 206

CYP5619A1_XP_012203945.1_ 138 VANAKVLTYKLSLLLILGLED--------NYDNSRELLDTYMLALRNSVRRADPTGVRSRDELIRTMINP 199

CYP5619A1_SDRG_14280.1_Sa 138 VANAKVLVFKLSLLLILGLED--------NYDNSRELLDTYMLALRNSVRRADPAGVRSRDELIRTMINP 199

CYP5619B2_SDRG_14277.1_Sa 153 ACLTRDMVFRIFLKVLYGVER-------HDGNKFRVLLDDFIVSIRRSSKHADPHGVRCRTQILDELIRP 215

CYP5619B4_XP_012203942.1_ 153 ACLTREMVFRIFLKVLYGVER-------HDANKFR--------------KRPDPHGVSCRTQILDELIRP 201

CYP5619B3_OQR84819.1_Achl 152 ALLTRNLVFRIFLQVLYGVEM--------LDDRHRVALDEFIASIRRSSKAPDPHGVSCRTRILEELIRP 213

CYP5619C3_OQR84833.1a_Ach 150 ALLSKKLVFKVFLAVLYGIETDTPAEYEAKYDHFRDVVDGYIHAIPKSAKAPDADGLRYKARAIDELIAP 219

CYP5619C2_OQR84821.1_Achl 151 GLLSKKLVFKVFLAVLFGLTNIPPIEYDTKYDQYRDEVDGFIAGISKSATAPDAHAVACKQRLITELIGP 220

CYP5619C1_XP_012203939.1_ 150 ALSCKKTVFKVFLAILYGITDMTPAEYDATYDPFRDLLDGFIRAIPKSSRGADAEGLVCKQRLLDELVAP 219

CYP5619C1_SDRG_14273.1_Sa 150 ALSCKKTVFKVFLAILYGITNLTPAEYDAKFDPFRDLLDSFIRAIPKSSKGADAEGLVCKQRLLDELVAP 219

CYP5619B1_SDRG_14281.1_Sa 148 ALSCKKMVFHVFMATLLGLEN--------VDDEYRELVEAFVSSIRKSARKPDTTGMDARTQVVEELIRP 209

CYP5619B1_XP_012203946.1_ 148 ALSCKKLVFHVFSATLLGLEN--------VDDEYRELIETFVSSIRKSARKPDATGMDARTQVVEELIRP 209

CYP5619B2_OQR84828.1a_Ach 149 AQNSRRMVFDVFLAVLFGIEG--------SFDEHRDLLDTFVSAIRKSARKADAAGLEARRRIVEDLVRP 210

CYP5619F2_XP_009834503.1_ 143 AVNTKILVFKVLLAVLYGVEG--------EFDTYRRYVDDYVTAIKQSAKVTDEHGVTCRAKFIAEIIAP 204

CYP5619F1_XP_008879406.1_ 143 ALNTKILVFKILLAVLYGMEG--------EFDSFRTYIEDYVAAIKQSAKTTSAHGVTCRATFIAEILEP 204

CYP5619G1_XP_008878127.1_ 142 TASSKELVFKLLLAVLYGIEG--------DFDEYRPLVDEFVASIRKSAVKASPEGKAARDTIMNDLVIP 203

CYP5619A3_OQS07110.1_Thra 110 VANCKQLAFKLMLVVLLGLENDY----DDHRDDHRELLDTYILSLRDSTHRADPDGVRSRQHLLDDMINP 175

CYP5619A2_OQR84828.1b_Ach 257 VAYTKQLVFKLALLVLLGLED--------NYDHQREQLDTYMTALRNSTRRADPAGVTARAQLIAGLLNP 318

[Consensus_aa:](http://prodata.swmed.edu/promals3d/info/consensus.html) *hh*.*h***+**.*hh***F+***lh***L**.*llh***G***l***-**s........p.s.*@***R**.b*l***D**p*@l*..*l*pposp.ssspt*h*.*h***R**pp*ll*.p*ll*.**P**

[Consensus_ss:](http://prodata.swmed.edu/promals3d/info/consensus_ss.html) hhhhhhhhhhhhhhhh hh hhhhhhhhhhhhhhhhhhhhhhhhhhhhhhhhhhhhhhhhh

Conservation: 95 7 7 557 6 5 6 9 75 7 7 95 555 7 57 5

CYP5619D3_AIG56338.1_Achl 208 AVVAAHAR-------ATPKSCVVDALVEAGTVS-DEDLATELFQLLVHGIPGLEGLVVHSLTAIASVDGV 269

CYP5619D2_SDRG_14279.1_Sa 208 AIAASHARV----RAGAAPTCVLDALVAQNTMS-DSDLATEGFQLMAMGLLGLEGLVVHTITAMVSVDGV 272

CYP5619D1_SDRG_03324.1_Sa 208 AIASSLARV----RSGAPLTCVLDTVVAQGTVS-EADLALESFQLLAMGLPGLEGLVVHTITAMVSLDDV 272

CYP5619D1_XP_012194083.1_ 208 AIASSVARV----RSGASTTCVLDAVVAQGSVS-EADLVVESFQLLAMGLPGLEGLVVHTITAMVSHDDV 272

CYP5619D4_AIG56100.1_Achl 207 AITAARAR-------STPKPSVVDILVAMGSVA-DADLADEIFALLANGLPGLEGLVVHTLSTMASVEGV 268

CYP5619D5_AIG56283.1_Achl 207 AITAARAR-------STPKPSVVDSLVAMGSVA-EADLADEIFALLANGLPGLEGLVVHTLTTMASVEGV 268

CYP5619A1_XP_012203945.1_ 200 ALATSHDRV----HAGTPKPCALDYLVAAGCLS-DDDLRTELFHLLCMSLGGLECWVANCITAAASSADV 264

CYP5619A1_SDRG_14280.1_Sa 200 ALATSHDRV----HTGKPKPCALDHLVAAGVLS-DDDLRAELFHLLCMSLGGLECWVANCITAAASSTDV 264

CYP5619B2_SDRG_14277.1_Sa 216 AIANAQARA----SNKTPVPSVIDCLVANGKMT-PDVLETEAFHFLFAGFGGVACLATNILTAVATHPSA 280

CYP5619B4_XP_012203942.1_ 202 AIADARARA----ATKTPAPSVIDCLVTNGKMA-SDVLETEAFHFLFAGFGGVACLATNILTAVATHPSA 266

CYP5619B3_OQR84819.1_Achl 214 AIVKARARI----AADAPAPCVLDNLITAAKLD-ADALEVEAFHFLFAGFGGVACLATNVLTACATHPGV 278

CYP5619C3_OQR84833.1a_Ach 220 ALAASQARI----EAGTPRPCVLDYWVQHSGMQ-PDDICLEAFHALFAGLGGVQCLVVNTITAMATNPGA 284

CYP5619C2_OQR84821.1_Achl 221 AIVASQARV----KAGAPRPCVLDALVAGDGLS-DAQLRLEGLHMLFAGLGGVQCLVVNSLTVMAKFPDI 285

CYP5619C1_XP_012203939.1_ 220 ALAASQARV----AAKTPVPCFLDYMLGQTELT-PDVVHLEAFHALFAGLGGTQCLVVNTITALAQYPTV 284

CYP5619C1_SDRG_14273.1_Sa 220 ALAASQARV----EAKAPVPCFLDYMLGQTELT-PDVVHLEAFHALFAGLGGTQCLVVNTITALAQYPTV 284

CYP5619B1_SDRG_14281.1_Sa 210 AVREAKARV----AAQKPLPTVVEVLVADGRLS-DEELNLELFHALFAGLGGVTCLVINAVTACIELPAI 274

CYP5619B1_XP_012203946.1_ 210 AIREAKARV----AAEKPLPTVVDVLVADGRLS-DEELGLELFHALFAGLGGVTCLAINSITVCIELPAI 274

CYP5619B2_OQR84828.1a_Ach 211 AIRDARGRA----SVGEAKPVAIDALIADGKLG-DNELELELFHALFAGFSGVACLVVNSITAAIEWPEA 275

CYP5619F2_XP_009834503.1_ 205 AIAAAKANQT--KRQQQPLNSVLDVLVATGDLTDDDDLQNEMFHFMFAGFGGVSAAATNLITAVCVFPDI 272

CYP5619F1_XP_008879406.1_ 205 AIAAAKARQQVNATSSGPLESVLDVLVASGELN-DDDLKNEGFHIMFAGFGGLSAAATNLITAAVVFPEI 273

CYP5619G1_XP_008878127.1_ 204 AIEAAKVRV----AGGTPSPSALDHLVGLNQLA-DDDLGVEMFHVLFAGFGGLSCLATNLVTPLVTMPDV 268

CYP5619A3_OQS07110.1_Thra 176 ALQTSHERL----NQNSLKPCVLDFLVSQKKLS-DADLRIELFHLLTMGVGGLECWLANCITAAASSPDV 240

CYP5619A2_OQR84828.1b_Ach 319 ALATAHDRV----AAHAPKACVLDFLVGQGQLS-DDDLRVELFHMLCMSLGGLECWATNCITAAASNPAV 383

[Consensus_aa:](http://prodata.swmed.edu/promals3d/info/consensus.html) **A***l*.stps**R***h*.....s..s.st*hl***D***h***LV**t.sp*l*s.sss**L**.*h***E**.**F***hh***L***hh***G***h*s**G***l*pt*hhh*p*hl***T***hhh*p.ss*l*

[Consensus_ss:](http://prodata.swmed.edu/promals3d/info/consensus_ss.html) hhhhhhhhh hhhhhhhh hhhhhhhhhhhhh hhhhhhhhhhhhhhhh hhh

Conservation: 95 7 55 6 65 766 5 75 9979 5 959 77755 7 5

CYP5619D3_AIG56338.1_Achl 270 RAHLASARDVYMAKYYG-AAR-WDHFDDLGYGNQFLLEVQRTYTASPRQEYARATVDLKVLTPT-GTTIV 336

CYP5619D2_SDRG_14279.1_Sa 273 RGQLGSARDAYVSKYPN-GAH-WRHLDDLSVVNAYVNEVQRVYNASPRHTFARATKDFVVTNSS----SV 336

CYP5619D1_SDRG_03324.1_Sa 273 RGQMATARDAYTAKYPG-GAF-WSHLDDLDAVNQYVNEVQRVCGASPRHTFARATKDFSVPSGSGATVAV 340

CYP5619D1_XP_012194083.1_ 273 RGQMATARDAYTAQYPN-GAH-WSHLEDLDAVNQYVNEVQRVYGASPRHTFARATKDLTVPDGSGAMVAV 340

CYP5619D4_AIG56100.1_Achl 269 VANLATARDVYLAKYPG-AAR-WQHLDELGYANQFLLEVQRTYGAKPSHVFARATKELSV-----AGVKV 331

CYP5619D5_AIG56283.1_Achl 269 VANLATARDVYLAKYPG-AAR-WQHLDELGYANQFLLEVQRTYGAKPSHAFARATKELSV-----AGAKV 331

CYP5619A1_XP_012203945.1_ 265 LAQLTAGRDAFMTKYPAEADR-WSHLSDLGYVNSYIQEVKRTYVAGPSHMYARATKETDVHTSE-GTFRV 332

CYP5619A1_SDRG_14280.1_Sa 265 LAQLTAGRDAFITKYPAEADR-WSHLGDLGYVNNYIQEVKRTYVAGPSHMYARATKDTDVRTSE-GTFHV 332

CYP5619B2_SDRG_14277.1_Sa 281 RKDLLDARAEYVTKYDG-DAR-WAHFHDLGYVNLFILEVKRFYVAGPTAVFGRTKTDLEIPTKN-GVYKL 347

CYP5619B4_XP_012203942.1_ 267 RKDLLDARAEYVTKYDG-EAR-WAHFHDLGYVNLFILEVKRFYVAGPTAVFGRAKTDLEIPTKN-GVYKL 333

CYP5619B3_OQR84819.1_Achl 279 LPKLLEARAEFVTRYPTEDAR-FAHLDDLGYVNDFLLEVKRYYVAGPTTVFGRAAVDLEVKTSN-GVYHL 346

CYP5619C3_OQR84833.1a_Ach 285 AEKLHHTRAEYVLKYHSAEDR-SSHFDQLGYANQFLLEVKRFYMAGPSQLFGRTTAELTFQTPD-GTYSV 352

CYP5619C2_OQR84821.1_Achl 286 SEKLQEARAAFVTRCPTPADR-WRHFDQLGYANQFLLEVKRFYTAGPTQLFGRTATELTFQTPD-GTYSV 353

CYP5619C1_XP_012203939.1_ 285 AEKVHASRAKFVTKYH--EDR-WRHFDNLGYCNRFLLEVKRFYNAGPAQLFGRTTQELTFTTPD-GEFAI 350

CYP5619C1_SDRG_14273.1_Sa 285 AEKVHASRAKFVIKYH--DDR-WRHFDNLGYCNRFLLEVKRFYSAGPAQLFGRTTQELTFTTPD-GEFAI 350

CYP5619B1_SDRG_14281.1_Sa 275 REKVSAAREAFLAKYPNEDDR-WSHFADLGYMHHFILEVKRFYVAGPTQLYGRATDDLEISTAN-GSFKV 342

CYP5619B1_XP_012203946.1_ 275 REKVSAAREAYLTKYPNEDDR-WRHFADLGYMQHFLLEVKRFYVAGPTQLYGRATDDLEISTAD-GSFKV 342

CYP5619B2_OQR84828.1a_Ach 276 RARVFEARDAFFAKYPTEDDR-WSAV-ELGYIDMYLLEVKRFYVAGPTQIYGRAKEEVALTTAE-GTFTI 342

CYP5619F2_XP_009834503.1_ 273 RAKVLRARDDFLRQYDGRDESPWNHLDEMGYLNLFVLEVKRYFVAGPTQVYAKAARDLDLVTST-GVFRI 341

CYP5619F1_XP_008879406.1_ 274 RAQVFAARDKYLSKFG--DDR-WGHLDDLGYLNKYILEVKRFFLAGPTQVYAKAARDVDLVTSK-GVFHL 339

CYP5619G1_XP_008878127.1_ 269 REKILDARDQFLSKYTG-DTK-WDHLEDLGYINQYILEVKRFFVAGPTQSFAKAAVAFDVVTSK-GTFHI 335

CYP5619A3_OQS07110.1_Thra 241 LKQLTVARDTYLKKYSNEEDR-WRRFDDLGYVNWYIQEVKRVYIAGPSHVYARSTAQVDIVTSD-GTFRV 308

CYP5619A2_OQR84828.1b_Ach 384 LKQLTAARDAFMTKHPTEDAR-WAHFQDLGYVNRYISEVKRVYVAGPSHLYARAAKTTDVHTSE-GAFTV 451

[Consensus_aa:](http://prodata.swmed.edu/promals3d/info/consensus.html) ..p*lh*.**AR**s.*@h*s**KY**ss.ss**+**.**W**.**H***h*c**-LGY***h***N**.*@l*b**EV**p**R***h***Y**s**A**t**P**sp*h@*t**R**t*h*.**-***h*p*l*.**T**sp.**G**s*h*.*l*

[Consensus_ss:](http://prodata.swmed.edu/promals3d/info/consensus_ss.html) hhhhhhhhhhhh hhhhh hhhhhhhhhhhh hhh eeeeeeeeeee eee

Conservation: 665 9 6 95 5 57 59 9 957 99 5 779

CYP5619D3_AIG56338.1_Achl 337 PKNRLKVGVLECLNKDAKRWPNPTSFDPTRFA------------------------SANTS---AYAFAP 379

CYP5619D2_SDRG_14279.1_Sa 337 PKHSLTAALLDCLNYNAARWPSPAQFQVARFA------------------------GANPS---AYEFAP 379

CYP5619D1_SDRG_03324.1_Sa 341 PKNRLTVVLLDCINNDPKRWPSPEQFQPARFA------------------------AANTS---AYGFAP 383

CYP5619D1_XP_012194083.1_ 341 PKNRLTVALLDCINHDPKRWPSPEQFQPARFA------------------------TANTS---AYGFAP 383

CYP5619D4_AIG56100.1_Achl 332 PKNRLTAVLLECLNQDPGRWPEPARFDPSRFA------------------------VANTS---AYEFAP 374

CYP5619D5_AIG56283.1_Achl 332 PKNRLTAVLLECLNQDPGRWPEPGRFDPSRFA------------------------IANTS---AYAFAP 374

CYP5619A1_XP_012203945.1_ 333 PKGCLVAAALDGTNKHPSVWANPTKFDPSRFS--------------------------STKVDMAFGFCP 376

CYP5619A1_SDRG_14280.1_Sa 333 PKGCLVAAALDGTNKHPSVWANPTKFDPSRFS--------------------------TAKVDMAFGFCP 376

CYP5619B2_SDRG_14277.1_Sa 348 PKGCLAAAGLEATNRHPDVWTDPNLFNPNRFRD-----------------------LGHVRTTKPHAFCP 394

CYP5619B4_XP_012203942.1_ 334 PKGCLAAAGLEATNRHPDVWTDPNLFNPNRFRD-----------------------LGHVRTTKPHAFCP 380

CYP5619B3_OQR84819.1_Achl 347 PKGCLAAAGLEATNKHPDVWANPHDFNPDRF-------------------------KDLDMASHAHRFCP 391

CYP5619C3_OQR84833.1a_Ach 353 PKGALAVAGLNATNKHPEVWADPRVFNPDRF-------------------------ADFDADADLYKLCP 397

CYP5619C2_OQR84821.1_Achl 354 PKGALAVAGLNATNKHPEVWADPSVFNPDRF-------------------------ANFDTTTDLYTLCP 398

CYP5619C1_XP_012203939.1_ 351 PKGVLAVAGLDATNRHPDVWTDPSVFNPDRFD------------------------NGFNEATDLYKLCP 396

CYP5619C1_SDRG_14273.1_Sa 351 PKGVLAVAGLDATNRHPDVWTDPSVFNPDRFD------------------------NGFSEASDLYKLCP 396

CYP5619B1_SDRG_14281.1_Sa 343 PKGCLATAGLEVTSKHPDVWSDPHTFNPDRFAPQDASTT---PVDPDAFKDGA---RDVTAPDMMYKFCP 406

CYP5619B1_XP_012203946.1_ 343 PKGCLATAGLEATSKHPDVWSDPHTFNPDRFAPQDASAQPSGSVDPDELKDGA---RDVTAPGLMYKFCP 409

CYP5619B2_OQR84828.1a_Ach 343 PKGCLATAGLETTNKHPGVWADPHVFNPDRFAADK----------TAVAKDGT---IDVTADEMAYKFCP 399

CYP5619F2_XP_009834503.1_ 342 PEGALVMAGLEATNRDPDTWPSPDSFDPTRF------------------TQAD---VDGMHMTRPFSFCP 390

CYP5619F1_XP_008879406.1_ 340 SKGSLVMAGLEATNHDPDVWAAPNTFDPSRF------------------NDADIDAAHGVKNTRKYSFCP 391

CYP5619G1_XP_008878127.1_ 336 PKGCLVAAGLETTAFDAEVWPNPDNFDPSRFD------------------------NNDDLSALQFKLCP 381

CYP5619A3_OQS07110.1_Thra 309 PKGALVAAALDTTNKHPKVWTNPGEFNPNRF-------------------------NKWDETKGMYTFCP 353

CYP5619A2_OQR84828.1b_Ach 452 HAGVLVAAALDGTDKHPSVWPDPTKFNPDRF---------------------------GAKVDMSYAFCP 494

[Consensus_aa:](http://prodata.swmed.edu/promals3d/info/consensus.html) **PK**s.**L***hh***A**.**L-***hh***N**pc**P**p.**W**ss**P**..**F**s**P**s**RF**..........................s.s.....*@*.**F**t**P**

[Consensus_ss:](http://prodata.swmed.edu/promals3d/info/consensus_ss.html) eeee hhh hhh hhh hhh eee

Conservation: 55 5 9 7 76 95 759 997 99 7 6 9 9 5 7 57 9 7 75 9

CYP5619D3_AIG56338.1_Achl 380 YAMNLLA-DRRRGVGEALSQLVLQTHLVSLWDFAWTMAPRQSYALADSP-NPSPVDALTTDGFFVA---- 443

CYP5619D2_SDRG_14279.1_Sa 380 FALNDLV-DRRAGRREGLSRLILQTHVVSLLDFAAVMAPLQSYALDDGL-NPLPVDLLTTVGFHYA---- 443

CYP5619D1_SDRG_03324.1_Sa 384 FAIDDLV-HRAEGRREGLSRLILQSHVVSLLDFVAVMAPLQSFALGDGV-NPLPIDLLTTVSFRYV---- 447

CYP5619D1_XP_012194083.1_ 384 FAIDDLV-HRVEGRREGLSRLILQSHVVSLLDFVAVMAPLQSFALGDGV-NPLPIDLLTTVSFRYA---- 447

CYP5619D4_AIG56100.1_Achl 375 FAMNLLT-DRPHGIREALTTTVLQTHVVSLFDFVWSMAPHQNYTVEAGV-NAGPVDGLMTVSFRAA---- 438

CYP5619D5_AIG56283.1_Achl 375 FAMNLLT-DRPHGMREALTTTVLQTHVVSLFDFVWSMAPHQNYTVEAGV-NAGPVDGLMTVGFRAA---- 438

CYP5619A1_XP_012203945.1_ 377 HAIGAVA-DRR-CAGEELSTLILQSFMVSLFDFMWKMLPHQDYTLDTTLVNPMPKGGLMVVGFHRR---- 440

CYP5619A1_SDRG_14280.1_Sa 377 HAIG--A-DRR-CAGEELSTLILQSFMVSLFDFMWKMLPHQDYTLDTTLVNPMPKGGLMVVGFHRR---- 438

CYP5619B2_SDRG_14277.1_Sa 395 HAFGESS-HRR-CAGEDLTTLILQSTVVSLYDFVWQMVPNQDYKLAVGSSTPTPVGQLMAVGFHRR---- 458

CYP5619B4_XP_012203942.1_ 381 HAFGALS-HRR-CAGEDLTTLILQSTIVSLFDFVWQMVPNQDYKLAVGVSTPTPVGQLMAVGFHRR---- 444

CYP5619B3_OQR84819.1_Achl 392 HAFGEAS-HRR-CAGETLTTVILQTIVVSLFDFVWQMVPGQNYALQEGVATPTPVDQLMAVGFHRR---- 455

CYP5619C3_OQR84833.1a_Ach 398 HAIGKTNGGRR-CAGQDLATAVMQASLVSLFDFKWTFVPGQDFTLETGKSTPMPVGNIMVTAFQHR---- 462

CYP5619C2_OQR84821.1_Achl 399 HSIGKMVGGRR-CAGQDLATAVMQASLVSLFDFKWTFAPGQDFTLETGKSTPMPVGNIMVTAFQHR---- 463

CYP5619C1_XP_012203939.1_ 397 HAIGKTTGGRK-CAGRDLATLVLQASLVSLFDFKWTLVPNQDLSLEEGKSTPMPKGLLMASAFTHR---- 461

CYP5619C1_SDRG_14273.1_Sa 397 HAIGKTTGGRK-CAGRDLATLVLQASLVSLFDFKWTLVPNQDLSLEEGKSTPMPKGLLMASSFTHR---- 461

CYP5619B1_SDRG_14281.1_Sa 407 HSIGIA---RR-CAGEGLTTLVLQCFVVSLFDFIWQMVPGQNYQLEEKSSTPTPIGQLMAVGFHRR---- 468

CYP5619B1_XP_012203946.1_ 410 HSIGTA---RR-CTGEGLTTLVLQCFVVSLFDFIWQMVPGQNYQLEEKSSTPTPVGQLMAVGFHRR---- 471

CYP5619B2_OQR84828.1a_Ach 400 HSIGSA---RR-CLGEGLTSLVLQCAFVSLLDFVWQMVPDQSYALDEKSATPTPTGQLMAVGFRRR---- 461

CYP5619F2_XP_009834503.1_ 391 HGFGS---HRR-CAGEQLTTVIMQSVLVSLFDFTWKMIPGQEYALQPHSVTAVPIGQLMGVNFHRRLNED 456

CYP5619F1_XP_008879406.1_ 392 HGFGDVR-NRR-CAGEELSTIVMQSLLVSYFDFTWKMVPGQNYTLQPHSVTAVPIGLLMAMGFQRQQ--- 456

CYP5619G1_XP_008878127.1_ 382 HGIGSTS-NRR-CAGETLTTLVCQALVVSLFDFTWNMVPGQDYELDENTSIPTPRGGLKAVGFRRR---- 445

CYP5619A3_OQS07110.1_Thra 354 HSVGS---DRR-CPGEQLSTVVLQSFMVSLFDFMWKMIPKQDYSLDTKAVNPMPRGGLMVVGFHRR---- 415

CYP5619A2_OQR84828.1b_Ach 495 HAVGAVA-NRR-CPGEELSTLVLQSFLVSLFDFMWKMVPTQDYTLDTTLVNPMPKGGLMVVGFHRR---- 558

[Consensus_aa:](http://prodata.swmed.edu/promals3d/info/consensus.html) *@*t*h*s.*h*s.p**R+**.t..**E**.**L**oo*llh***Q**s*hl***VSL***@***DF***h***W**p**M***h***P**.**Q**s*@*.**L**.....s**P***h***P**.s.**L***hhh*t**F**p......

[Consensus_ss:](http://prodata.swmed.edu/promals3d/info/consensus_ss.html) hhhhhhhhhhhhhhhhhhhhee eeee eeeeeee

Conservation: 59 59 9 9 979559576777 575 9

CYP5619D3_AIG56338.1_Achl 444 --------PGAVVDT----EAWRRLHQPDVQLYNASIENPLL---AAGDKRLDFFTHSAIQLFNTRYNLW 498

CYP5619D2_SDRG_14279.1_Sa 444 --------PGVAHSSNAYDDAWRRLRQPSAKLYNSSIESPL-----SSDKRLDFLTHSMIQLLNVRFATW 500

CYP5619D1_SDRG_03324.1_Sa 448 --------PGVVQGDI---DAWRRLHHPSAKLYNGSLENPLL---AASDKRLDFWTHSMIQLFNVRFETW 503

CYP5619D1_XP_012194083.1_ 448 --------PGVVQQDV---DAWRRLHHPNAKLYNGSLENPLL---AASDKRLDFWTHSMIQLFNVRFETW 503

CYP5619D4_AIG56100.1_Achl 439 --------PGAVVDT----EAWRRLTRPYPEAFNSSLDNPL-----AADPRLDFLTHSLIQLGNTRFTLW 491

CYP5619D5_AIG56283.1_Achl 439 --------PGAVVDT----EAWRRLTRPYPEAFNSSLDNPL-----AADPRLDFLTHSLIQLINTRFNLW 491

CYP5619A1_XP_012203945.1_ 441 --TDLSASMVEVAGSE---EDWKFLSLPDAKVYRDDKETLHD---MFADERLDLWTHLMLKLLGKKQSMW 502

CYP5619A1_SDRG_14280.1_Sa 439 --TDLSASMVEVAGSE---EDWKFLSLPEAKVYRDDKEALHD---MFADERLDLWTHLMLKLLAKKQSMW 500

CYP5619B2_SDRG_14277.1_Sa 459 --TDDAVEIIGTVGSK---ADWKFLNLPEAKELVGTAMD------LYDDARLDLWTRLMIKLIGKKQAVW 517

CYP5619B4_XP_012203942.1_ 445 --TDDAAEIIGTVGSN---ADWKFLNLPESKEL----------------------------------ALW 475

CYP5619B3_OQR84819.1_Achl 456 --TDDAVE-FGVAGSQ---GDWKFLNLPEAKALVGGASD------LYDDARLDLWTRLMIKLIGKKQAAW 513

CYP5619C3_OQR84833.1a_Ach 463 -------HEVGEDGCD--VANWHLLNMPEAKALAGVAAQVSD---DEDDARLDLWTRLMIKLIGKKQSRW 520

CYP5619C2_OQR84821.1_Achl 464 -------HEVGEDGCD--VANWHLLNMPEAKALAGVAAEVSE---DEDDARLDLWTRLMIKLIGKKQSRW 521

CYP5619C1_XP_012203939.1_ 462 -------HSADETECD--VSDWHLLNLPEAKALVGIAGTVSD---DEDDARLDLWTRLMIKLIAKKQARW 519

CYP5619C1_SDRG_14273.1_Sa 462 -------HSESETECD--VADWHLLNLPEAKALVGIAGTVSD---DEDDARLDLWTRLMIKLIAKKQARW 519

CYP5619B1_SDRG_14281.1_Sa 469 --TLDDVVTFGTAGSD---EDWHFLSLPQAKELVGSGTAD-----LYDDARMDLWTRLMIKLIGKKQATW 528

CYP5619B1_XP_012203946.1_ 472 --ALDRVVTFGTAGSD---EDWHFLSLPQARELVGCGAAD-----LYDDARMDLWTRLMIKLIGKKQAAW 531

CYP5619B2_OQR84828.1a_Ach 462 ----SPGTEHSVAGSE---ADWKFLRCPEAEALVGGATGD-----LFGDARLDLWTRLMIKIIGKKQATW 519

CYP5619F2_XP_009834503.1_ 457 DPSTPEVETYGIVGTQ---DDWKFLRRPDVQELTGVNAAE-----YFDDSRLDLWTRLMIQLISKKQTLW 518

CYP5619F1_XP_008879406.1_ 457 -DDGSGNLDYGVVGSH---ADWKFLRRPDVQELTGHNAAE-----YFDDSRLDLWTRLMIKLISKKQSVW 517

CYP5619G1_XP_008878127.1_ 446 ----DAVTSYGVAGTD---DDWTFLKLPEAKAIVSVHGGWGDSDGLFADPRLDLWTELMIKLIGKKQAKW 508

CYP5619A3_OQS07110.1_Thra 416 --TAASDDMVQVAGSE---ADWKFLSLPEAKVYADSKETMYE---MFSDERLDVWTHLMIQLLSKKQERW 477

CYP5619A2_OQR84828.1b_Ach 559 --TDLSASMVEVAGSE---ADWHFLSLPEASVYRNSSETLHD---VFADERLDVWTHLMLKLVAKKQSKW 620

[Consensus_aa:](http://prodata.swmed.edu/promals3d/info/consensus.html) ..........s*hh*ssp....s**W+**.**L**p.**P**p*h*c.*h*sts..s......*h*.s**D**.**RLD***h***WT+**.**MI**p**L***l*sp**+**bs.**W**

[Consensus_ss:](http://prodata.swmed.edu/promals3d/info/consensus_ss.html) hhhhh eeee hhh hhhhhh hhhhhhhhhh hhhhhhhhhhh

Conservation: 5 9 7 7 9 7 9 9 79 77 7577659997 77 5 57779 796 979 7 9799

CYP5619D3_AIG56338.1_Achl 499 VKPSASA-ITVPKVQKVLPKRKLYGTAIQIPTEDEDVDI-PKALLEAAKLIQDTAPFVDNFDAKWLPGED 566

CYP5619D2_SDRG_14279.1_Sa 501 VTPTAAASITVPKSQKPLAKQTLHGTSIQIPVDDEDVSI-PKVLLDGAKLLQDTAPFVDNFDDSWVPGED 569

CYP5619D1_SDRG_03324.1_Sa 504 VTPTAAASIKVPTTQKNLPKRTLYGTSIQIPTEDEDVAI-PKVILESAKLLQDTAPFVDNFDAKWAPGED 572

CYP5619D1_XP_012194083.1_ 504 VTPTAAASIKVPTTQKTLPKRTLYGTSIQIPTEDEDVAI-PKVVLESAKLLQDSAPFVDNFDAKWAPGED 572

CYP5619D4_AIG56100.1_Achl 492 VKPSAATAITIPTTQGVLPKRTLYGTTIQIPTVDEDVKI-PKELLEAVKLLQDTAPFVDNFDATWRPGED 560

CYP5619D5_AIG56283.1_Achl 492 VKPSAATAITIPTTQGVLPKRTLYGTTIEIPTVDEDVTI-PKALLEAGKLLQDTAPFVDNFDAKWRPGED 560

CYP5619A1_XP_012203945.1_ 503 NRPFANQAITVPKYQKTLPKITLYGLKIQIPTEDEDWPADPWNEVAMVKFLRDSCPLGDDFEHTWLPGED 572

CYP5619A1_SDRG_14280.1_Sa 501 NKPFANQAITAPKYQKTLPKITLYGLKIQIPTEDEDWPSDPWNEVATVKFLRDSCPLGDDFEHTWLPGED 570

CYP5619B2_SDRG_14277.1_Sa 518 DRPYANQILRIPQHQKPLPKITLIQTNIDIATEDEDWPNQPWLEIQQSNFLRDHAPFVDNFEHTWLPGED 587

CYP5619B4_XP_012203942.1_ 476 DRPYANQILSIPQHQKTLPKITLIQTHIEIATEDEDWPSQPWIEIQQSNFLRDYAPFVDNFELTWLPGED 545

CYP5619B3_OQR84819.1_Achl 514 DRPYADQILSIPKFQKVLPKITLIQTNIEIATEDEDWPNQPWIEIQQSNFLRDHAPFVDNFNAKWVPGED 583

CYP5619C3_OQR84833.1a_Ach 521 NKPVANEVLTIPKSQVTLPKITLIQTDIQVATEDEDWPNQPWLEIQQSNFLRDYAPFVDNFEHTWLPGED 590

CYP5619C2_OQR84821.1_Achl 522 NKPVANEVLTIPKSQVTLPKITLIQTDIQVATEDEDWPNQPWLEIQQSNFLRDYAPFVDNFEHTWLPGED 591

CYP5619C1_XP_012203939.1_ 520 NKPAANEVLTVPRFQRELPKMTLIQTNIQVATEDEDWPNQPWLEIQQSNFLRDYAPLVDDFEHTWLPGED 589

CYP5619C1_SDRG_14273.1_Sa 520 NKPVANEVLTVPQFQKELPKMTLIQTNIQVATEDEDWPNQPWLEIQQSNFLRDYAPFVDNFEHTWLPGED 589

CYP5619B1_SDRG_14281.1_Sa 529 DRPFVESCLTIPKHQKVLPKLTLIQTSIEIPTEDEDWPKQPWLEIKQSNFLRDHAPFIDDFKHTWLPGED 598

CYP5619B1_XP_012203946.1_ 532 DRPFVDSCLKIPKHQKVLPKLTLIQTSIEIPTEDEDWPKQPWIEIKQSNFLRDHAPFIDDFTHTWLPGED 601

CYP5619B2_OQR84828.1a_Ach 520 NCPPVNSLLTVPKHQTTLPKITLIQTEIEIPTEDEDWPHQSWFEVQQSNFLRDHAPFIDDFVHKWLPGED 589

CYP5619F2_XP_009834503.1_ 519 NRPYATTALSVPQHQQVLDKITLIQTNIQIPIVDEDWPCQPWLEIQQTNLLRDHAPFVDDFSHLWLPAED 588

CYP5619F1_XP_008879406.1_ 518 NRPYAQSALSLPMEQVVLDKITLIQTQIEIPTVDEDWPSQPWLEIQQSNLLRDHAPFVDDFDHPWLPAED 587

CYP5619G1_XP_008878127.1_ 509 NRPYADTALMLPKNKQPLVKLTLAQTSIQVPTEDEDWPTQSWVEVKQANFLRDHAPFKDDFVHKFLPGED 578

CYP5619A3_OQS07110.1_Thra 478 NRPYANISIKVPQKQVKLKKVTLDGTKVEIPTEDEDWPSDPWFEVKTVEFLRDSCPMDDDFKYQWVPGED 547

CYP5619A2_OQR84828.1b_Ach 621 NRPFANSSITIPKYQKELPKITLFGLKIQVPTEDEDWPADPWVEVAMVKFLRDSCPFVDNFTDTWLPGED 690

[Consensus_aa:](http://prodata.swmed.edu/promals3d/info/consensus.html) s**+P***h***A**s.s*l*p*l***P**p.**Q**.s**L**s**K**.**TL***h*.**T**p**I**p*l*s**T-DED***h*s..**P**b*h*b*l*p.sp*h***L**p**D**p**APF***l***D**s**F**p*h*p**W***l***PGED**

[Consensus_ss:](http://prodata.swmed.edu/promals3d/info/consensus_ss.html) hh eeehhhhhh hhhhhh hhhhhhhhhhhhhhheeee hhhhhhh

Conservation: 59 7979979 9997959997999999969965959 97977599 7 999995 7 65 7799

CYP5619D3_AIG56338.1_Achl 567 MEDYVLSKVGHMWPRVRVHWDDRYSDRALELLVFHGLGQHLVTKLP-HAHDDGSYYTVALDFLGALEVRS 635

CYP5619D2_SDRG_14279.1_Sa 570 MEGYVLSKVGRMWPRVRVHWDDRYSDRALELFVFNGLGQHMVTKLS-AAHSDGSYYTATTSFLETLDVRP 638

CYP5619D1_SDRG_03324.1_Sa 573 MEGCVLSKVGRMWPRVRVHWDDRYSDRALELLVFNGLGQHMVQKLA-TAHDDGSYYTVATNYLASIEVRT 641

CYP5619D1_XP_012194083.1_ 573 MEGYVLSKVGRMWPRVRVHWDDRYSDRALELLVFHGLGQHMVQKLA-TAHDDGSYYTVATNFLASIEVRA 641

CYP5619D4_AIG56100.1_Achl 561 MEAYVLSKVGRMWPQVRVHWDDRYSDRALELLVFHGIGQHMVTKLP-QPHADGSYYTVALNFMDALEVRA 629

CYP5619D5_AIG56283.1_Achl 561 MEAYVLSKVGHMWPQVRVHWDDRYSDRALELLVFQGLGQHMVTKLP-QPHADGSYYTVALNFMDALEVRA 629

CYP5619A1_XP_012203945.1_ 573 MERYVMSKVGSMWPRVNVHWNDRYSDRALELLVFNGLGQHLVTKLP-TAHDDGSYYGICLDFMQALDVRP 641

CYP5619A1_SDRG_14280.1_Sa 571 MERYVMSKVGSMWPRVNVHWNDRYSDRALELLVFNGLGQHLVTKLR-TAHDDGSYYGICLDFMQALDVRP 639

CYP5619B2_SDRG_14277.1_Sa 588 MERYVMSKVGSMWPRVNVHWNDRYSDRALELLAFNGFGQHLLTKLP-EAHDDGSYYGICLNFMKSLEVRP 656

CYP5619B4_XP_012203942.1_ 546 MERYVMSKVGHMWPRVNVHWNDRYSDRALELLAFNGFGQHLLMKLP-EAHDDGSYYGICLGFVKGLEVRP 614

CYP5619B3_OQR84819.1_Achl 584 MERYVLSKVGHMWPRVNVHWNDRYSDRALELLAFNGLGQHLLQKLP-EAHSDGSYYGIELDFMQVLEVRP 652

CYP5619C3_OQR84833.1a_Ach 591 MERYVMSKLGHMWPRVNVHWNDRYSDRALELLAFHGFGQHLLQKLP-ESHDDGSYYGIELDFMRTLEVRP 659

CYP5619C2_OQR84821.1_Achl 592 MERYVMSKVGKMWPRVNVHWNDRYSDRAVELIAFNGFGQHLLTKLP-EAHDDGSYYGIELNFMRTLEVRP 660

CYP5619C1_XP_012203939.1_ 590 MERYVMSKVGHMWPRVNVHWNDRYSDRALELLAFNGFGQHLLMKLP-EAHDDGSYYGICLGFMKGLEVRP 658

CYP5619C1_SDRG_14273.1_Sa 590 MERYVMSKVGSMWPRVNVHWNDRYSDRALELLAFNGFGQHLLTKLP-EAHDDGSYYGICLNFLKGLEVRP 658

CYP5619B1_SDRG_14281.1_Sa 599 MERYVMSKLGHMWPRVNVHWNDRYSDRALELLAFNGLGQHLLMKLP-EAHDDGSYYGICLDFMNVLEVRP 667

CYP5619B1_XP_012203946.1_ 602 MERYVMSKLGHMWPRVNVHWNDRYSDRALELLAFNGFGQHLLMKLP-EAHDDGSYYGICLDFMSVLEVRP 670

CYP5619B2_OQR84828.1a_Ach 590 MERYVLSKVGHMWPRVNVHWNDRYSDRALELLAFNGLGQHLLQKLP-EAHSDGSYYGIELDFMQVLEVRP 658

CYP5619F2_XP_009834503.1_ 589 GERYVMSKVGHMWPRVNVHWNDRYSDRALELLVFHGLGSHLVQKLP-QAHADGSYYGVLLNVMQGLEVRP 657

CYP5619F1_XP_008879406.1_ 588 GERYVMSKVGHMWPRVNVHWNDRYSDRALELLVFHGLGSHLVQKLP-QEHDDGSYYGLLLNVMQGLEVRP 656

CYP5619G1_XP_008878127.1_ 579 GERYVMSKVGHMWPRVNVHWNDRYSDRALELLVFNGLGSHLVQKLPTEDPTDGSYYGVLLNFMQVLDVRP 648

CYP5619A3_OQS07110.1_Thra 548 KERYVMSKVGHMWPRVLVHWNDRYSDRALELLAFNGMGQHLVQKLE-KAHDDGSYYSITLEFMQGIEVRP 616

CYP5619A2_OQR84828.1b_Ach 691 MERYVMSKVGHMWPRVNVHWNDRYSDRAFELLGFHGLGQHMLTKLP-AAHADGSYYTIGLDFMQVLEVRP 759

[Consensus_aa:](http://prodata.swmed.edu/promals3d/info/consensus.html) b**E**.**YV***h***SKVG+MWPRV**p**VHW**s**DRYSDRALELL***h***F**p**G***h***GQH***hl*p**KL**s.pt**H**s**DGSYY**s*l*.**L**s**F***h*ps**LEVR**s

[Consensus_ss:](http://prodata.swmed.edu/promals3d/info/consensus_ss.html) hh eee hhhhhhhh hhhhhhhhhh eehhhhhhh hhhhhhhh

Conservation: 959 996679 966699 9 7 7 5 979 97699777 779979995959 9779 77 5

CYP5619D3_AIG56338.1_Achl 636 GFAKLGADGFFTKDGKVTKIVRQGVTYLPGAAKWEYAKLCFRGSLNAKITAVDHLIGLHVTVGNYMTTAT 705

CYP5619D2_SDRG_14279.1_Sa 639 GYAVTGADAYFDKNGKVTKIVRLGKTFRPADAQWEYVKMCFRSSVANKVTAVDHLIGLHVTVGNYMTTAS 708

CYP5619D1_SDRG_03324.1_Sa 642 GYAITGADAFFDKNGKVTKIVRLGKTIRPIDASWEYVKMCFRSSLVSKITAVDHLIGLHVTVGNYMTTGS 711

CYP5619D1_XP_012194083.1_ 642 GYAITGADAFFDAKGKVTKIVRLGKTIRPTDAAWEYAKMCFRSSLVSKITAVDHLMGLHVTVGNYMTTAS 711

CYP5619D4_AIG56100.1_Achl 630 GYAKAGADAFFTSKGKVTKIVRQGVTYVPGDAGWEYAKLCFRGSVIIKITAVDHLIGLHVTAGNYLTTAS 699

CYP5619D5_AIG56283.1_Achl 630 GYAKAGADAFFTSKGKVTKIVRQGVTYVPGDAGWEYAKLCFRGSVNIKITAVDHLIGLHVTAGNYLTTAS 699

CYP5619A1_XP_012203945.1_ 642 GYAKYGADAYFNAKGKVTKIIRLGKTVHPGDDDWEYAKLCFRGSLQTKVTALDHLLGIHITVANGLVTST 711

CYP5619A1_SDRG_14280.1_Sa 640 GYAKYGADAYFNAKGKVTKIVRLGKTVHPGDEDWEYAKLCFRGSLQTKVTALDHLLGIHITVANGLVTST 709

CYP5619B2_SDRG_14277.1_Sa 657 GYAKYGADAFFTSKGKVTKIIRGDIASRPGDSGWEYAKLCFRGSLQTKVTAVDHLLGIHATVANIMVVAN 726

CYP5619B4_XP_012203942.1_ 615 GYAKYGADVYFTAKGNVTKIVRGDITSRPGDAGWEYAKLCFRGSLQTKVTAVDHLLGIHATVANIMVIAN 684

CYP5619B3_OQR84819.1_Achl 653 GYAKYGASAYFNQKGKVTKIIRAGVTSHPGDKDWEYFKLAFRGSLQTKVTAVDHLLGIHATVANIMVIAN 722

CYP5619C3_OQR84833.1a_Ach 660 GFAKYGADAYFNENGKVTKIVRGGVTSRPGDATWEYAKLCFRGSLQTKITAVDHLLGVHATVANIMVMAN 729

CYP5619C2_OQR84821.1_Achl 661 GFAKYGANAYFNKKGKVTKIVRGGVTSRPGDATWEYAKLCFRGSLQTKITAVDHLLGIHATVANIMVIAN 730

CYP5619C1_XP_012203939.1_ 659 GYAKYGADAYFNAEGKVTKIVRGDITARPGDDSWAYAKLCFRGSLQTKITAVDHLLGVHATVANIMVIAN 728

CYP5619C1_SDRG_14273.1_Sa 659 GYAKYGADAFFSAEGKVTKIVRGDVTVRPGDDNWAYAKLCFRGSLQTKITAVDHLLGVHATVANIMVIAN 728

CYP5619B1_SDRG_14281.1_Sa 668 GYAKYGADAYFTAKGKVTKIIRGGVTSRPGEDGWEYAKLCFRGSLQTKVTAVDHLLGIHATVANYMVTSI 737

CYP5619B1_XP_012203946.1_ 671 GYAKYGADAYFNAKGKATKIVRGGVTSRPGEDGWEYAKLCFRGSLQTKVTAVDHLLGIHATVANYMVTSI 740

CYP5619B2_OQR84828.1a_Ach 659 GYAKYGASAYFNQKGKVTKIVRGGSTFVPGDAGWEYAKLCFRGSLQTKVTAVDHLLGIHVTVANYMVTSA 728

CYP5619F2_XP_009834503.1_ 658 GFAKYGADAFFNKHGKLVKIQRGDKTYTNTHDDWAYIKMTFRGTLMTKVTAVDHLLGVHVTAANYLVTAS 727

CYP5619F1_XP_008879406.1_ 657 GFAKYGADAFFDKHGHVVKIKRGDQTYTKTDAAWEYVKMCFRGSLQTKVTAVDHLLGVHATAANYLVTSS 726

CYP5619G1_XP_008878127.1_ 649 GFAKYGADAFFDKQGKLIKIIRGDKTYTKTDVEWEYVKMCFRGSLQTKVTAVDHLLGIHVTVANYLVTAS 718

CYP5619A3_OQS07110.1_Thra 617 GYATYGADAFFNSKGKVTKIIRKGVTYRPKDDGWEYAKLCFRGSLNTRVTAVDHLLGIHLTVANYLVTSS 686

CYP5619A2_OQR84828.1b_Ach 760 GLAKYGADAFFDRNGKVTKIVRHGTTSRPGDDNWEYFKLCFRGSLQTKVTALDHLLGIHITVANQLVTST 829

[Consensus_aa:](http://prodata.swmed.edu/promals3d/info/consensus.html) **G***@***A**p*h***GADA***@***F**s.p**GKVTKI***l***R**.s.**T**.p**P**s**D**ss**WEY***h***K***h***CFRGSL**.o**K***l***TAVDHL***l***G***l***H***h***TV**t**N***hhhh*ts

[Consensus_ss:](http://prodata.swmed.edu/promals3d/info/consensus_ss.html) eeeee eeeeeee hhhhhhhhhhhhhhhhhhhhhhhhhhhhhhhhhhhhhhh

Conservation: 997977 9979975999799565797 95 975956 979955 5 96 69 77 5 77799

CYP5619D3_AIG56338.1_Achl 706 REQLPPKHPVRRLLKPFTFRAVAINYEASNVLFAPKGLLQRAFPLTEKGMAQTWVTALKDLKLETFPQHI 775

CYP5619D2_SDRG_14279.1_Sa 709 REQLPPTHPLRRLIKPFTFRAVAINYEASKLLFAPKGILHRAHPYSEKGLKDTWAMALQSLKLEPFPVHM 778

CYP5619D1_SDRG_03324.1_Sa 712 REQLPPTHPLRRLIKPFTFRAVAINYDASIALFAPKGMLHRAFPYTEKGLKDTWAMALKSLTLEPFPVHL 781

CYP5619D1_XP_012194083.1_ 712 REQLPPAHPLRRLIKPFTFRAVAINYDASIALFAPKGMLHRAFPFTEKGLKDTWAMALKSLTLEPFPVHL 781

CYP5619D4_AIG56100.1_Achl 700 REQLPPAHPLRRLLKPFTFRAAAINYDASSALFAPKGILHRAFALSEKGMAQTWAAAQTMIRLETFPQHI 769

CYP5619D5_AIG56283.1_Achl 700 REQLPPAHPLRRLLKPFTFRAAAINYEASNSLFAPKSVLHRAFAFSEKGMAQAWAAAQSMIRLETFPQHI 769

CYP5619A1_XP_012203945.1_ 712 REQLPPTHPLRRLLKPFTFRSVIINYNASYALFWPKGMLHRAFSLSVEGMQQTWELGLANFKYETFPEHK 781

CYP5619A1_SDRG_14280.1_Sa 710 REQLPPTHPLRRLLKPFTFRSVIINYNASYALFWPKGMLHRAFSLSVEGMQQTWELGLANFKYETFPEHK 779

CYP5619B2_SDRG_14277.1_Sa 727 REQLPPTHPLRRLIKPFTFRSVAINYGAGRALFWPKGMLQRAYALTDKGMKQT----------------- 779

CYP5619B4_XP_012203942.1_ 685 REKLPPTHPLRRLIKPFTFRSVAINYGAGRALFWPKGMLQRAYALTDKGMKQTWDFGLANFKYETFPEHK 754

CYP5619B3_OQR84819.1_Achl 723 REQLPPTHPLRRLIKPFTFRSVAINYGAGRALFWPKGMLQRAYALTSNGMKQTWEYGLSHFKYETFPERR 792

CYP5619C3_OQR84833.1a_Ach 730 REQLPPTHPLRRLIKPFTFRSIAINYGAGRALFWPKGMLQRAYALTDKGMKQTWDFGLSHFKYETFPEHI 799

CYP5619C2_OQR84821.1_Achl 731 REQLPPTHPLRRLIKPFTFRSVAINYGAGRALFWPKGMLQRAYALSTLGMKQTWDYGLSHFKYETFPERR 800

CYP5619C1_XP_012203939.1_ 729 REQLPPTHPLRRLIKPFTFRSIAINYGAGRALFWPKGMLQRAYALTDKGMKQTWDIGLANFKYETFPEQI 798

CYP5619C1_SDRG_14273.1_Sa 729 REQLPPTHPLRRLIKPFTFRSIAINYGAGRALFWPKGMLQRAYALTDKGMKQTWDIGLANFKYETFPEHI 798

CYP5619B1_SDRG_14281.1_Sa 738 REQLPPAHPVRRLLKPFTFRSVAINFGAGRSLFWPKGMLQRAYALTDKGMKQTWEYGLANFKYETFPERK 807

CYP5619B1_XP_012203946.1_ 741 REQLPPAHPVRRLLKPFTFRSVAINFGAGRSLFWPKGMLQRAYALTDKGMKQTWEYGLANFKYETFPERK 810

CYP5619B2_OQR84828.1a_Ach 729 REQLAPAHPLRRLLKP------------PRA-FRPKGMLQRAYALTTDGMKQTWEYGLSHFKYETFPEHR 785

CYP5619F2_XP_009834503.1_ 728 REKLPVRHPLRRLLKPFTFRSVSINYGAGRALFWPNGMLQRAFALTTAGMKQTWEFGLTQFEYATFPETM 797

CYP5619F1_XP_008879406.1_ 727 REKLPVNHPLRRLIKPFVFRSVAINYSAGRALFWPNGMLQRAYALTTAGMKSTWEFGLSQFEYATFPDRI 796

CYP5619G1_XP_008878127.1_ 719 REQLAVNHPLRRLFKPFTFRTVSINFSAGRALFWPNGMLQRAYALTNSGMKQTWEYGLSHFVYAPFPDRV 788

CYP5619A3_OQS07110.1_Thra 687 REQLPPNHPLRRLIKPFTFRSVIVNFAASWGLIWPRAMLQRAFAVSEKGIDTLWKTGLASFKYEPFPEHM 756

CYP5619A2_OQR84828.1b_Ach 830 REQLPPTHPLRRILKPFTFRSVIINYNASYALFWPKGMLHRAYSLNEKGMQQTWDFGLANFKYETFPEHK 899

[Consensus_aa:](http://prodata.swmed.edu/promals3d/info/consensus.html) **REQLPP**s**HPLRRL***l***KPFTFR**t**V**t**INY**s**A**t.t**LF***h***P+GML**p**RA***@*t*l*opp**G***h*pp**TW**.*h*t**L**.p*h*c*h***E**s**FP**p**+**b

[Consensus_ss:](http://prodata.swmed.edu/promals3d/info/consensus_ss.html) h hhhhhhhhhhhhhhhhhhhhhhhh eeeeee hhhhhhhhhhhh hhhhhhhh

Conservation: 69 797 5959 77 579 95 95 955997 5 9 79 79 5 7 5

CYP5619D3_AIG56338.1_Achl 776 ARQQVDTMTLPFHHDGTDYWNIVRRFTSNYLDLYYKDDTAVTSDASLQSFWRTLSAQLP-MPLPPLGLAV 844

CYP5619D2_SDRG_14279.1_Sa 779 ARQNIDTLKLPFHEDGMDFWTIVRGFTGEYLNLYYESDEDVTRDASTQAFWAFLDKQLP-TPLGALSLES 847

CYP5619D1_SDRG_03324.1_Sa 782 ARQQVDTITLPYHEDGADYWEIVRTFVSEYLDLYYTSNDDVTHDVSIQALWTFLNKQLP-TPLGVLSLDN 850

CYP5619D1_XP_012194083.1_ 782 ARQQVDTITLPYHEDGADYWKIVRTFVSEYLDLYYKSDDDVTRDASIQALWAFLNKQLP-TPLGVLSLEN 850

CYP5619D4_AIG56100.1_Achl 770 ARQGVDSLSLPFHEDGLAYWDIVHSFASDYLGLYFPSDAAVTGDASVVAFWKALAAV---TPLPALSRTA 836

CYP5619D5_AIG56283.1_Achl 770 ARQGVDSLSLPFHEDGLAYWDIVHSFASDYLGLYFPSDAAVTGDASVVAFWKALAAV---TPLPALSRTA 836

CYP5619A1_XP_012203945.1_ 782 ARQNIDTTTLPYHEDGMDFWLIVRGFVGSYIDLYYPCDESLTQDTAVQAFWSYLKATLPPNSIRPLSKDN 851

CYP5619A1_SDRG_14280.1_Sa 780 ARQNIDTTTLPYHEDGMDFWLIVRGFVGSYIDLYYPCDESLTQDTAVQAFWSYLKTTLPPNSIRPLSKDN 849

CYP5619B2_SDRG_14277.1_Sa 780 ------TQDAPAHRHNDA---------------------------------------------------- 791

CYP5619B4_XP_012203942.1_ 755 ARQNIDTTTLPFHEDGMDYWQICRSFVSNYVDLYFKSEDALQNDTD-----------------------N 801

CYP5619B3_OQR84819.1_Achl 793 ARQNIDTTTLPFHEDGMDYWNIVRTFVNDYLDLYFKTDANVGGDANVVQFWGFLRSKLPADAMRELTLEN 862

CYP5619C3_OQR84833.1a_Ach 800 ARQNIDTTTLPFHEDGMDYWTIVRTFVSNYVDLYYKAEADVENDADLHAFWSYIGSMLPVP-MRKLTLEN 868

CYP5619C2_OQR84821.1_Achl 801 VRQNIDTVTLPFHEDGMDYWNIVRTFVSNYVDLYYKADSAIANDEHVRKFWSFLDDKLPFD-MRPLTLEN 869

CYP5619C1_XP_012203939.1_ 799 ARQNIDTATLPFHEDGMDYWHICRSFVSNYVDLYFKSEDALQSDTDVHAFWTFLSTKLPVP-MRTLTLEN 867

CYP5619C1_SDRG_14273.1_Sa 799 ARQNIDTTTLPFHEDGMDYWHICRSFVSNYVDLYYKSEDALQNDTDVHAFWTFLSTKLPVP-MRTLTLEN 867

CYP5619B1_SDRG_14281.1_Sa 808 ARQSIDTVTLPFHEDGIEYWQICRTFANDYVDLYYKSEDATSADADLKRFWTFLDEKLPFT-MRPLNLEN 876

CYP5619B1_XP_012203946.1_ 811 ARQNIDTLTLPFHEDGIEYWQICRTFANDYINLYYKSEDAISADADLKRFWTFLDEKLPFA-MRPLNLEN 879

CYP5619B2_OQR84828.1a_Ach 786 ARQNIDTTTLPFHEDGMDYWNIVRTFVNDYLDLYFKTDTDVTGDVHVNKFWSFLNDKLPFD--------- 846

CYP5619F2_XP_009834503.1_ 798 AKQEIDTLTLPFHQDGLDYWHIVYKFVANYVDLYYPSDDDVAMDVDVGKFWRYMGELSPAP-LPDLTKSH 866

CYP5619F1_XP_008879406.1_ 797 ARQQIDTLTIPFHEDGLDYWNIMIKFVSSYVDLYYPDDASIQHDDDVVAFWSNLTAVSPAP-LPDLNKSN 865

CYP5619G1_XP_008878127.1_ 789 KAQQIDTFTLPFHQDGLDYWAIVFSFVSKYIDLYFADDAAIAGDTDVVNFWTYVTSVSPVP-LPPVSKAS 857

CYP5619A3_OQS07110.1_Thra 757 ERQKVDTISMPFHEDGLDYWYICHTFVSDYLNLYYANDEALTQDTAVRAFWNFLNEKLPTG-VRPLSLAN 825

CYP5619A2_OQR84828.1b_Ach 900 ARQNIDTLTLPYHEDGMDYWTIVRKFVSNYLDLYYKCDESLTQDTAVQAFWSYLKSTLPTGAVRPLNREN 969

[Consensus_aa:](http://prodata.swmed.edu/promals3d/info/consensus.html) **ARQ**p*l***DT***h*o**LP***@***H**c**DG***h***-YW**p**I***h***+**p**F***h*tp**Y***l*s**LY***@*.s**-**.s*l*p.**D***h*s*l*..**FW**p*h***L**s..**LP**.s.*h*.s**L**sb.s

[Consensus_ss:](http://prodata.swmed.edu/promals3d/info/consensus_ss.html) hh hhhhhhhhhhhhhhhhhhhhhhh hhhhh hhhhhhhhhhhhh hhh

Conservation: 779 6 7799779999997799977999999 799997 77797 97 967957997 79 7 9977

CYP5619D3_AIG56338.1_Achl 845 LKDTTAIGIFLVTAMHNHLGGIAEYVSDPAFCPTAWVEGEIAGRPGSCVCAAVLMAGTGYLQPNVMEDFS 914

CYP5619D2_SDRG_14279.1_Sa 848 LKDVVAHGIFLVTAMHNHLGGIAEYVSDPAFCPVSWVEGELAGRPGAAVRTALIMSGTGYPQPSILEDFS 917

CYP5619D1_SDRG_03324.1_Sa 851 LKDVVAHSIFLVTAMHNHLGGIAEYVSDPAFCPVSWVEGELSGRPGNAVRAALIMSGTGFPQPNILEDFS 920

CYP5619D1_XP_012194083.1_ 851 LKDVVAHSIFLVTAMHNHLGGIAEYVSDPAFCPVSWVEGELSGRPGNAVRAALIMSGTGFPQPNILEDFS 920

CYP5619D4_AIG56100.1_Achl 837 LVDATATAIFLVTAMHNHLGGIAEYVSDPAFCPAAWVEGELAGRPGTSVRSAIIMSGTGYLQPNVMEDFT 906

CYP5619D5_AIG56283.1_Achl 837 LVDATATAIFLVTAMHNHLGGIAEYASDPAFCPTAWVEGELAGRPGTSVRSAIIMAGTGYLQPNVMEDFT 906

CYP5619A1_XP_012203945.1_ 852 IKDFVAHAIFLVSSMHNHLGTIAEYVSDPAFCPSAWVEGELAGRPGPCVRGALIMAATGFTQPSIKEDFS 921

CYP5619A1_SDRG_14280.1_Sa 850 IKDFVAHAIFLVSSMHNHLGTIAEYVSDPAFCPSAWVEGELAGRPGPCVRGALIMAATGFVQPSIKEDFS 919

CYP5619B2_SDRG_14277.1_Sa 792 ------------------------------------------------------------AVP------- 794

CYP5619B4_XP_012203942.1_ 802 LKDFVAHFIFLVSSMHNHLGTIAEYVSDPAFCPSAWVEGELAGRPGTGVRLALIMTATGFAQPAITEDFS 871

CYP5619B3_OQR84819.1_Achl 863 LKDFVAHFIFLVSSMHNHLGTIAEYVSDPAFCPSAWVEGELAGRPGTAVRLALIMTATGFTQPAITEDFS 932

CYP5619C3_OQR84833.1a_Ach 869 LKDFVAHFIFLVSSMHNHLGTIAEYVSDPAFCPSSWVEGELAGRPGTAVRLALIMTATGFAQPAITEDFS 938

CYP5619C2_OQR84821.1_Achl 870 LKDFVAHGIFLVSSMHNHLGTIAEYVSDPAFCPSAWVEGELAGRPGNAVRLALIMTATGFAQPAITEDFS 939

CYP5619C1_XP_012203939.1_ 868 LKDFVAHFIFLVSSMHNHLGTIAEYVSDPAFCPSAWVEGELAGRPGTGVRLALIMTATGFAQPAITEDFS 937

CYP5619C1_SDRG_14273.1_Sa 868 LKDFVAHFIFLVSSMHNHLGTIAEYVSDPAFCPSAWVEGELAGRPSTGVRLALIMTATGFAQPAITEDFS 937

CYP5619B1_SDRG_14281.1_Sa 877 LKDFLAHGIFLVSSMHNHLGTIAEYVSDPAFCPSAWVEGELAGRPGTGVRLALIMTATGFTQPDITEDFS 946

CYP5619B1_XP_012203946.1_ 880 LKDFLAHGIFLVSSMHNHLGTIAEYVSDPAFCPSAWVEGELAGRPGTGVRLALIMTATGFTQPAITEDFS 949

CYP5619B2_OQR84828.1a_Ach ----------------------------------------------------------------------

CYP5619F2_XP_009834503.1_ 867 LKDFMSQGIFLVSSMHNHLGTIAEYVSDPAFCPSAWVEGELSARPGNAVRLALIMSATGFTQPSITEDFS 936

CYP5619F1_XP_008879406.1_ 866 LKDFLAEGFFLVSSMHNHLGTIAEYVSDPAFCPSAWVEGELSARPGNAVRLALIMTATGFTQPSITEDFS 935

CYP5619G1_XP_008878127.1_ 858 LKDFIAQGIFLVSSMHNHLGTIAEYVSDPAFCPSAWVEGDHAAPPGNAVRLALIMTATGFTQPAITEDFS 927

CYP5619A3_OQS07110.1_Thra 826 LKDFITHAIVLVSAMHNHLGTLAEYVPDPAFCPSSWVEGEMAGRPGTSVRAALLMAATGFTQPAITEDIS 895

CYP5619A2_OQR84828.1b_Ach 970 LKDFVAHAIFLVSSMHNHLGTIAEYVSDPAFCPSSWVEGELAGRPGTGVRLALIMTATGFTQPAITEDFS 1039

[Consensus_aa:](http://prodata.swmed.edu/promals3d/info/consensus.html) **LKD***hh***A***h*.**IFLV**ot**MHNHLG**s**IAEYVSDPAFCP**ot**WVEGEL**t**GRPG**st**VR***h***ALIM**st**TG***@h***QP**s**I***h***EDFS**

[Consensus_ss:](http://prodata.swmed.edu/promals3d/info/consensus_ss.html) hhhhhhhhhhh hhhhh hh hhhhhhhhhhhhhhh hh

Conservation: 657996 77555 97 57 77 99 9 55775777 7799756

CYP5619D3_AIG56338.1_Achl 915 HVLLDDAAKAVARNFTTSLQAFTDIVRSRNAQRLLAYRAFDATIMDMAIGI----------- 965

CYP5619D2_SDRG_14279.1_Sa 918 HVLLDDAAKAVAHRFTTSLQSFVMVVEARNAQRVLPYQGFNPAVMDMAIGI----------- 968

CYP5619D1_SDRG_03324.1_Sa 921 HVLLDDAAKAVAHRFTASLQAFVHVVEARNAQRIHPYQAFNPAVMDMAIGI----------- 971

CYP5619D1_XP_012194083.1_ 921 HVLLDDAAKAVAHRFTASLQAFVQVVEARNAQRILPYQAFNPLVMDMAIGI----------- 971

CYP5619D4_AIG56100.1_Achl 907 HVLLDDKAKAVARRFTAALRGLVGVVQSRNAKRVLPYRGFDPEIIDMAIGI----------- 957

CYP5619D5_AIG56283.1_Achl 907 HVLLDDKAKAVARRFTAALRSLVGVVQSRNAKRVLPYRGFDPEIIDMAIGI----------- 957

CYP5619A1_XP_012203945.1_ 922 HIMLDENAKAVCRKFTADVCAYAAVVEARNSKRQHPYQAFNPNTMEMAVSI----------- 972

CYP5619A1_SDRG_14280.1_Sa 920 HIMLDDAAKAVCRKFTADVCAYAAVVEGRNTKRQHPYQAFNPNTMEMAVSI----------- 970

CYP5619B2_SDRG_14277.1_Sa --------------------------------------------------------------

CYP5619B4_XP_012203942.1_ 872 HIMLDDAGKAVCQAFTAAVTAQIAVVDARNATRVQPYQSFNPKTMEMAVTVAVDATGSSKRR 933

CYP5619B3_OQR84819.1_Achl 933 HVMLDDAAKKVAKQFTKAVTEQIAVVDARNASRVQPFQSFNPKTMEMAVSI----------- 983

CYP5619C3_OQR84833.1a_Ach 939 HIMLDDAAKAVCLAFTKAVTDQIAVVDARNASRVQPFQSFNPN------------------- 981

CYP5619C2_OQR84821.1_Achl 940 QIMLDDAAKAVCKKFTADVTAFIDVVDTRNLSRPQAYQSFNPKTMEMAVSI----------- 990

CYP5619C1_XP_012203939.1_ 938 HIMLDDDAKAVCQAFTAAVTAQIAVVDARNATRVQPFQSFNPKTMEMAVSM----------- 988

CYP5619C1_SDRG_14273.1_Sa 938 HIMLDDAAKAVCQAFTAAVTAQIAVVDARNATRVQPFQSFNPKTMEMAVSI----------- 988

CYP5619B1_SDRG_14281.1_Sa 947 HLMLDDAAKAVCKAFTAAVIAQIAVVDARNATRVQPFQSFNPKTMEMAVSI----------- 997

CYP5619B1_XP_012203946.1_ 950 HIMLDDDAEAVCQAFTAAVTAQIAVVDARNATRVQPFQSFNPKTMEMAVSI----------- 1000

CYP5619B2_OQR84828.1a_Ach --------------------------------------------------------------

CYP5619F2_XP_009834503.1_ 937 HIMLDDKAKALVKTFTADLYAQIKVVDARNANRVQPFQSFNPKAMEMAVSI----------- 987

CYP5619F1_XP_008879406.1_ 936 HVMLDDAAKAIVRTFTTDVKAQIKVVDARNATRVQPFQSFNPKTMEMAVSI----------- 986

CYP5619G1_XP_008878127.1_ 928 HVMLDNAAKDLVRTFTADLFKLIDVIDARNTTRVQPFQSFNPKTMEMAVSI----------- 978

CYP5619A3_OQS07110.1_Thra 896 GIMLDDKAKAVCKRFSEALTKQIDVVNERNKHRVQIYQSMNPAVMEMAVSI----------- 946

CYP5619A2_OQR84828.1b_Ach 1040 HVMLDDAAKKIAKQFTADVTDFIAVVDKRNASRPQAYQSFNPKTMEMAVSI----------- 1090

[Consensus_aa:](http://prodata.swmed.edu/promals3d/info/consensus.html) **H***lh***LDD**.**AKA***l*tp.**FT***h*s*l*.sb*l*.**VV**ct**RN***h*p**R***l*bs*@***Q**t**FNP**.*h***M-MA***l*t**I**...........

[Consensus_ss:](http://prodata.swmed.edu/promals3d/info/consensus_ss.html) hh hhhhhhhhhhhhhhhhhhhhhhhhh eee

**Table S4: Protein sequences used to create EXXR and CXG motif logos.** The EXXR and CXG motif amino acid patterns of 23 CYP5619 P450s are presented below.

**EXXR motifs sequences**

EVKR

EVKR

EVKR

EVKR

EVKR

EVKR

EVKR

EVKR

EVKR

EVKR

EVKR

EVKR

EVKR

EVKR

EVQR

EVQR

EVQR

EVQR

EVKR

EVKR

EVKR

EVQR

EVQR

**CXG motif sequences**

---APFAIDDLVHRVEGRR

---APFAIDDLVHRAEGRR

---APFALNDLVDRRAGRR

---APYAMNLLADRRRGVG

---APFAMNLLTDRPHGIR

---APFAMNLLTDRPHGMR

---CPHGFG-DVRNRRCAG

---CPHAFG-ALSHRRCAG

---CPHSIGKMVGGRRCAG

---CPHAIGKTTGGRKCAG

---CPHAIGKTTGGRKCAG

---CPHAIGKTNGGRRCAG

YTFCPHSVG---SDRRCPG

YKFCPHSIG---SARRCLG

YKFCPHSIG---TARRCTG

YKFCPHSIG---IARRCAG

FGFCPHAIG---ADRRCAG

FSFCPHGFG---SHRRCAG

--LCPHGIG-STSNRRCAG

--FCPHAFG-EASHRRCAG

--FCPHAFG-ESSHRRCAG

--FCPHAIG-AVADRRCAG

--FCPHAVG-AVANRRCPG

**Figure S2.** **Z-score estimation for CYP5619A1 refined model on the ProSA-WEB server.** The z-score of the modeled protein (-7.61) is represented as a black dot. The score is represented by a dot displayed in a plot that contains the z-scores of all experimentally determined protein chains in the current PDB. In the plot, groups of structures from X-ray crystallography and NMR are displayed in dark and light blue respectively and serve as a basis for comparison with the modeled protein.


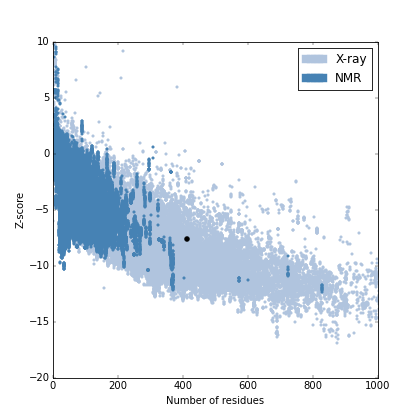


**Figure S3. ERRAT result for CYP5619A1 refined model.**

**
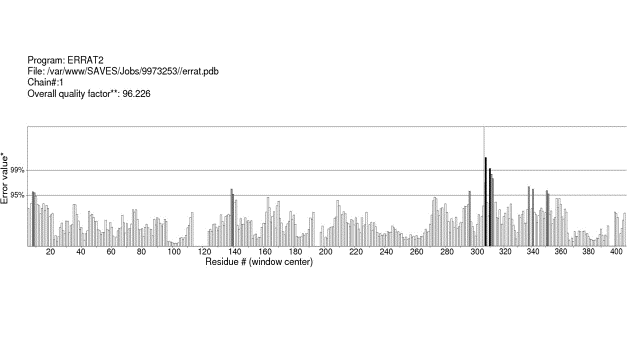
**

*On the error axis, two lines are drawn to indicate the confidence with which it is possible to reject regions that exceed that the error value.

**Expressed as the percentage of the protein for which the calculated error value falls below the 95% rejection limit. Good high-resolution structures generally produce values around 95% or higher. For lower resolutions (2.5 to 3 Å), the average overall quality factor is around 91%.

**Figure S4.** **Verify 3D result for CYP5619A1 refined model.** 86.36% of the residues had an averaged 3D-1D score ≥ 0.2. Pass: At least 80% of the amino acids scored ≥ 0.2 in the 3D/1D profile.


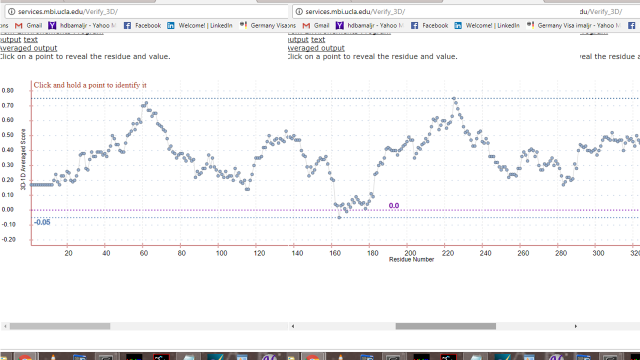


**Figure S5. RMSD analysis of CYP5619A1 model.** (A) The superimposed structures (CYP5619A and CYP120A1) are colored by RMSD, with the green color indicating close proximity between α-carbons, while lime-green and white colors indicate a wide gap and a total mismatch between the query sequence and the template, respectively. (B) RSMD calculation; the overall RMSD is 0.951 Å. (C) RMSD plot of all aligned residues showing only a few aligned residues with RMSD over 2 Å.

**
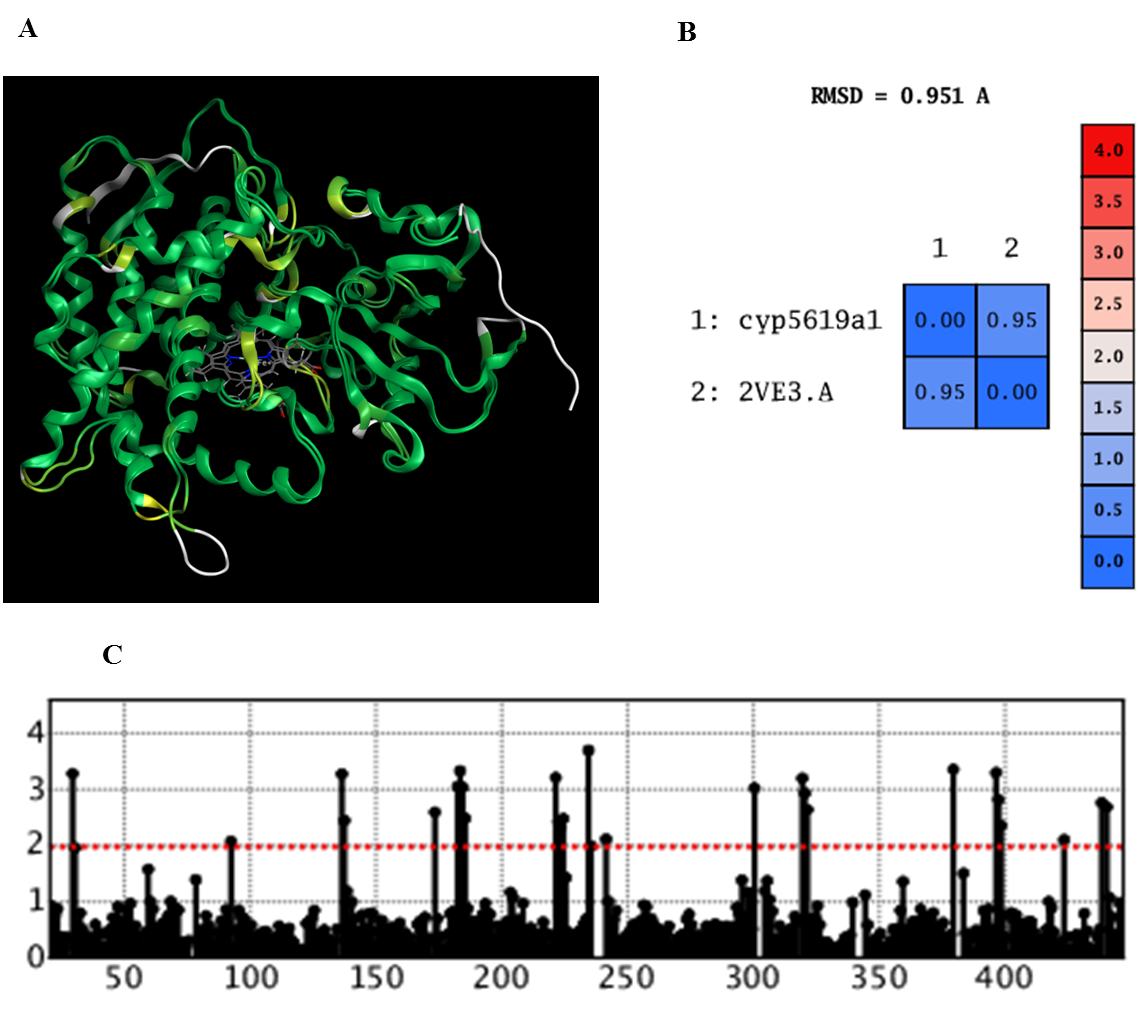
**

**Table S5. List of amino acids that are part of the CYP5619A1 active site.**

Tyr9; Gly12; Asp13; Arg14; Ser15; Ala16; Met18; Asp19; Phe23; Val24; Leu25; Glu26; Phe37; Cys38; Gly39; Pro40; Leu61; Leu65; Leu69; Pro74; Met158; Leu161; Arg162; His225; Cys228; Met229; Gly232; Gly233; Trp237; Arg291; Thr292; Tyr293; Val294; Ala295; Gly296; Pro297; Ser298; His299; Met300; Tyr301; Ala328; Asp330; Gly331; Asn333; Lys334; His335; Pro336; Leu404; Asp405; TThr406; Thr407; Leu408; Val409

**Figure S6. Graphic comparison of binding energies for each conformation of the best two ligands.** The binding energy for each of the 10 conformations of linoleic acid is plotted in orange. The binding energy for each of the 10 conformations of malachite green is plotted in blue. The trend for each ligand is plotted as a dashed line of the same color.

**Table S6. Amino acids interacting with ligands (fatty acids and malachite green).**

| **Amino acid** | **Ligands** | **Interactions with total number of ligands** |
| --- | --- | --- |
| Tyr9 | ACD | 1 |
| Arg14 | ACD | 1 |
| Leu61 | PLM, STE, DCR, OLA, LNL, ACD, EPA, MGR | 8 |
| Leu65 | PLM, STE, OLA, EPA, MGR | 5 |
| Leu69 | PAM, EIC | 2 |
| Pro74 | MYZ, PAM, EPA, MGR | 4 |
| Met158 | PLM | 1 |
| Leu161 | EIC | 1 |
| Arg162 | MYR, PLM, STE, DCR, MYZ, PAM, OLA, EIC, LNL, EPA | 10 |
| His225 | PLM, MYZ, PAM, OLA, EIC, EPA | 6 |
| Cys228 | MYR, PLM, STE, MYZ, PAM, OLA, EIC, LNL, EPA | 9 |
| Met229 | MYR, PLM, STE, DCR, MYZ, PAM, OLA, EIC, LNL, ACD, EPA, MGR | 12 |
| Gly232 | MYR, STE, DCR, MYZ, PAM, OLA, EIC, LNL, EPA, MGR | 10 |
| Gly233 | STE, DCR, MYZ, PAM, OLA, EIC, LNL, ACD, EPA, MGR | 10 |
| Trp237 | STE, DCR, EIC, LNL, ACD, MGR | 6 |
| Pro297 | MYR, PLM, STE, DCR, MYZ, PAM, OLA, EIC, LNL, ACD, EPA, MGR | 12 |
| His299 | MYR, PLM, DCR, OLA, LNL, ACD, MGR | 7 |
| Met300 | MYR, OLA, LNL | 3 |
| Tyr301 | PLM, STE, OLA, LNL, MGR | 5 |
| Thr407 | DCR, EPA | 2 |
| Leu408 | EIC | 1 |

**Abbreviations:** MYR: myristic acid; PLM: palmitic acid; STE: stearic acid; DCR: icosanoic acid; MYZ: myristoleic acid; PAM: palmitoleic acid; OLE: oleic acid; EIC: linoleic acid; LNL: alpha-linolenic acid; ACD: arachidonic acid; EPA: eicosapentaenoic acid; MGR: malachite green.
